# Neurological and Head/Eyes/Ears/Nose/Throat Manifestations of COVID-19: A Systematic Review and Meta-Analysis

**DOI:** 10.1017/cjn.2021.180

**Published:** 2021-07-21

**Authors:** Aravind Ganesh, Isabella R. Reis, Malavika Varma, David G. Patry, Lara J. Cooke

**Affiliations:** Department of Clinical Neurosciences, University of Calgary Cumming School of Medicine, Calgary, Alberta, Canada; Department of Medicine, Faculty of Health Sciences, McMaster University, Hamilton, Ontario, Canada; Department of Emergency Medicine, University of Calgary Cumming School of Medicine, Calgary, Alberta, Canada

**Keywords:** COVID-19, Stroke, Sensory systems, Neurology – General, Guillain–Barre, Peripheral Neuropathy

## Abstract

**Background/Objective:**

Coronavirus disease 2019 (COVID-19) has been associated with various neurological and atypical head/eyes/ears/nose/throat (HEENT) manifestations. We sought to review the evidence for these manifestations.

**Methods:**

In this systematic review and meta-analysis, we compiled studies published until March 31, 2021 that examined non-respiratory HEENT, central, and peripheral nervous system presentations in COVID-19 patients. We included 477 studies for qualitative synthesis and 59 studies for meta-analyses.

**Results:**

Anosmia, ageusia, and conjunctivitis may precede typical upper/lower respiratory symptoms. Central nervous system (CNS) manifestations include stroke and encephalopathy, potentially with brainstem or cranial nerve involvement. MRI studies support CNS para-/postinfectious etiologies, but direct neuroinvasion seems very rare, with few cases detecting Severe Acute Respiratory Syndrome Coronavirus 2 (SARS-CoV-2) in the CNS. Peripheral nervous system (PNS) manifestations include muscle damage, Guillain–Barre syndrome (GBS), and its variants. There was moderate-to-high study heterogeneity and risk of bias. In random-effects meta-analyses, anosmia/ageusia was estimated to occur in 56% of COVID-19 patients (95% CI: 0.41–0.71, I2:99.9%), more commonly than in patients without COVID-19 (OR: 14.28, 95% CI: 8.39–24.29, I2: 49.0%). Neurological symptoms were estimated to occur in 36% of hospitalized patients (95% CI: 0.31–0.42, I2: 99.8%); ischemic stroke in 3% (95% CI: 0.03–0.04, I2: 99.2%), and GBS in 0.04% (0.033%–0.047%), more commonly than in patients without COVID-19 (OR[stroke]: 2.53, 95% CI: 1.16–5.50, I2: 76.4%; OR[GBS]: 3.43,1.15–10.25, I2: 89.1%).

**Conclusions:**

Current evidence is mostly from retrospective cohorts or series, largely in hospitalized or critically ill patients, not representative of typical community-dwelling patients. There remains a paucity of systematically gathered prospective data on neurological manifestations. Nevertheless, these findings support a high index of suspicion to identify HEENT/neurological presentations in patients with known COVID-19, and to test for COVID-19 in patients with such presentations at risk of infection.

## RÉSUMÉ

Manifestations neurologiques et atypiques dans des cas d’infection à la COVID-19 : un examen systématique et une méta-analyse.

**Contexte/Objectif:**

Les infections à la COVID-19 ont été associées à de multiples manifestations neurologiques mais aussi à des atteintes atypiques à la tête, aux yeux, aux oreilles, aux voies nasales et à la gorge (TYOVG). Nous avons ainsi cherché à examiner les preuves de ces manifestations et de ces atteintes.

**Méthodes:**

Dans le cadre de cet examen systématique et de cette méta-analyse, nous avons compilé des études parues jusqu’au 31 mars 2021. Ces études s’étaient penchées sur des atteintes non-respiratoires de type TYOVG ainsi que sur des atteintes au système nerveux central (SNC) et au système périphérique (SP) chez des patients atteints d’une infection à la COVID-19. Pour ce faire, nous avons inclus 477 études de synthèse qualitatives de même que 59 études constituant des méta-analyses.

**Résultats:**

Il est donc possible que des manifestations d’anosmie, d’agueusie et de conjonctivite précèdent les symptômes typiques qui affectent les voies respiratoires supérieures et inférieures. Quant aux atteintes du SNC, elles ont inclus des AVC et des cas d’encéphalopathie comportant potentiellement une atteinte du tronc cérébral ou des nerfs crâniens. Des études basées sur des examens d’IRM ont certes confirmé des étiologies para et post-infectieuses du SNC ; toutefois, une neuro-invasion directe semble être largement inusitée car seulement quelques cas de coronavirus 2 (syndrome respiratoire aigu sévère) ont été détectés dans le SNC. Des atteintes au SP ont inclus par ailleurs des dommages musculaires en plus de l’apparition du syndrome de Guillain-Barré (SGB) et de ses variantes. Soulignons aussi que l’hétérogénéité des études et le risque de biais étaient modérés à élevés. Dans des méta-analyses à effets aléatoires, il a été estimé que l’anosmie et l’agueusie survenaient chez 56 % des patients atteints d’une infection à la COVID-19 (IC 95 % : 0,41–0,71, I^2^ : 99,9 %), ce qui est plus fréquent que chez des patients n’en étant pas atteints (RC : 14,28 ; IC 95 % : 8,39–24,29 ; I^2^ : 49,0 %). On estime que des symptômes de nature neurologique sont survenus chez 36 % des patients hospitalisés (IC 95 % : 0,31–0,42 ; I^2^ : 99,8 %) ; des AVC ischémiques chez 3 % d’entre eux (IC 95 % : 0,03–0,04 ; I^2^ : 99,2 %) ; et des manifestations du SGB chez 0,04 % d’entre eux (0,033–0,047 %). Encore une fois, cela est plus fréquent que chez des patients n’étant pas atteints d’une infection à la COVID-19 (RC [AVC] : 2,53 ; IC 95 % : 1,16–5,50 et I^2^ : 76,4 % ; RC [SGB] : 3,43 ; IC 95 % : 1,15–10,25 et I^2^ : 89,1 %).

**Conclusions:**

Les données actuelles proviennent essentiellement de cohortes ou de séries rétrospectives, principalement des patients hospitalisés ou gravement malades. Elles ne sont donc pas représentatives de patients typiques vivant en communauté. Qui plus est, les données prospectives recueillies systématiquement au sujet des manifestations neurologiques restent rares. Néanmoins, nos constatations nous portent à rester éminemment prudents dans l’identification de manifestations neurologiques et d’atteintes de type TYOVG chez des patients dont une infection à la COVID-19 est connue et en ce qui regarde la nécessité de passer un test de dépistage.

## Introduction

There is a growing appreciation that various manifestations affecting the head/eyes/ears/nose/throat, (HEENT) central and peripheral nervous systems (CNS and PNS) may be seen in patients with coronavirus disease 2019 (COVID-19), the pandemic caused by the Severe Acute Respiratory Syndrome Coronavirus 2 (SARS-CoV-2).^1^

**Figure 1:**
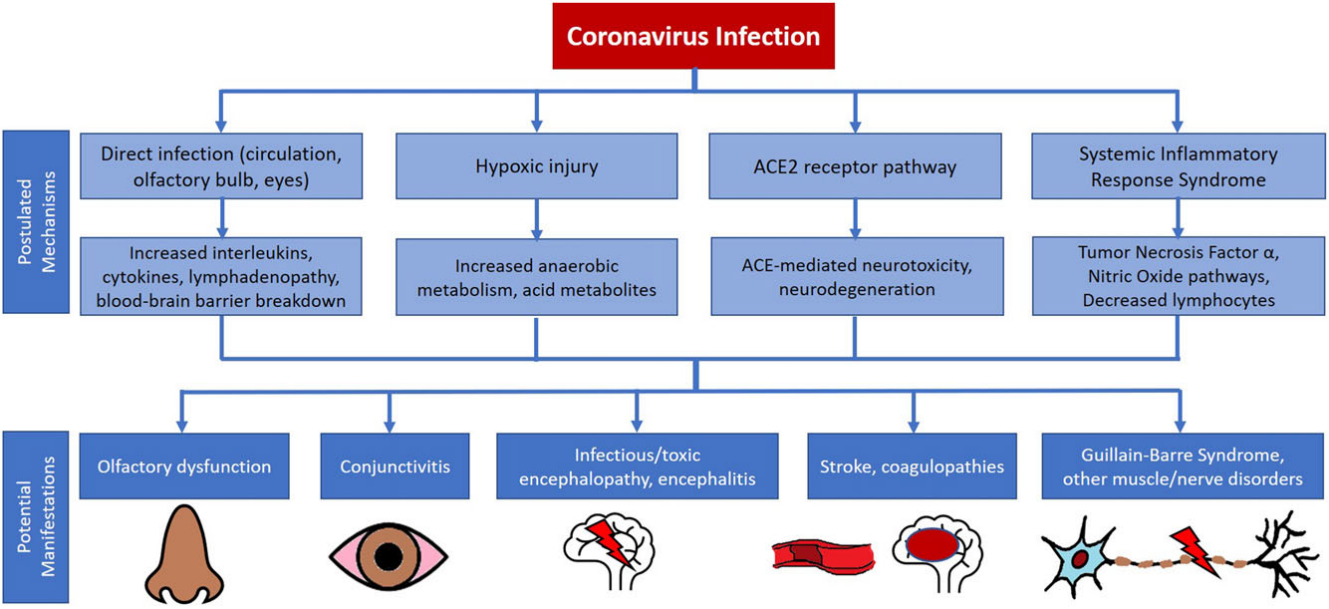
Summary of postulated mechanisms and consequent neurological and HEENT manifestations for coronavirus infections like COVID-19. The mechanisms summarized in this original figure were adapted from a figure by Wu and colleagues.^6^ Note that not all mechanisms feed into all the manifestations illustrated in this figure – for example, hypoxic injury is less likely to be a key mechanism in peripheral nerve disorders.

Prior outbreaks of the Severe Acute Respiratory Syndrome caused by SARS-CoV-1 and of the Middle East Respiratory Syndrome caused by MERS-CoV implicated coronaviruses in various neurological presentations, but mostly in small case reports or series. Reported manifestations included stroke, neuropsychiatric symptoms, seizures, polyradiculoneuropathy, and myopathies (Supplementary Table 1). Muscle symptoms were especially common with SARS; approximately one-third of patients manifested myalgias, elevated creatine kinase (CK),^2,3^ and rhabdomyolysis in some series.^4,5^ In parallel with these clinical reports, basic and translational science studies have indicated how coronaviruses like SARS-CoV-1, MERS-CoV, and the new SARS-CoV-2 may damage the HEENT and nervous systems **(**Figure 1 **)**.^6^ These include direct infection via the circulation or through a neuronal pathway (such as olfactory nerve/bulb invasion), hypoxic injury, modulation of angiotensin-converting enzyme type 2 (ACE2) receptors,^7,8^ and immune-mediated injury.^9^ However, with prior outbreaks, there was little evidence that these viruses are actually neuroinvasive, with just one report of SARS-CoV-1 detected in the brain tissue of a patient with neurological symptoms.^10^ The eyes may be another route of entry; coronaviruses can cause conjunctivitis, anterior uveitis, retinitis, and even optic neuritis in feline and murine models.^11^ The systemic inflammatory response syndrome (SIRS) precipitated by coronaviruses may also drive neurological/HEENT symptoms through the action of tumor necrosis factor α^12^ and nitric oxide, which may induce apoptotic responses, local demyelination, and axonal degeneration.^13^

While we are still beginning to understand these manifestations, 1 year after the onset of the pandemic, there is an urgent need for physicians to recognize these presentations as they may be the heralding or most prominent symptoms of COVID-19 in some patients. For neurologists, this includes recognizing not only symptoms directly relating to the CNS or PNS, but also HEENT symptoms relating particularly to altered vision, hearing, smell, or taste, which may not necessarily be neurological in origin but may nevertheless come to their attention. In this systematic review, we summarize the current evidence on the occurrence, range, and implications of such HEENT and neurological presentations in patients with COVID-19, drawing on the best available evidence. We also seek to estimate the frequency with which some of these presentations occur using meta-analysis, hypothesizing that they occur more commonly in patients with COVID-19 than those without.

## Methods

The systematic review was carried out according to the Meta-Analysis of Observational Studies in Epidemiology^14^ and the Preferred Reporting Items for Systematic Reviews and Meta-Analyses^15^ statements. The review has been registered with PROSPERO (CRD42021260869; available from: https://www.crd.york.ac.uk/prospero/display_record.php?ID=CRD42021260869). The authors AG (neurologist) and IR (health sciences undergraduate) searched PubMed (1946–March 31, 2021), Embase and Embase Classic (1947–March 31, 2021), and Google Scholar (since March 2020 for unindexed new papers) using the terms “coronavirus” or “COVID” or “SARS” or “MERS” combined using the AND function with “neurology”, “neurological”, “eye”, “ocular”, “ophthalmological”, “ear”, “nose”, or “ENT” (detailed strategy in the **Supplement**). Whereas the focus of our review was on the association of COVID-19 with HEENT/neurological symptoms, we went back as far as the index dates in PubMed and Embase to help contextualize our findings within the framework of what we know from prior coronavirus outbreaks like SARS and MERS (these older results are informed Supplementary Table 1 and Figure 1). We limited our exploration of HEENT symptoms to those that were not part of the typical or obvious symptom complex for upper or lower respiratory tract infections, such as sore throat or nasal congestion. Consequently, we did not include terms like “throat”, “pharynx”, or “mouth” and instead focused on symptoms relating to altered taste, smell, hearing, or vision that are more relevant to neurologists, or likely to be encountered by them, even if not necessarily neurological in origin.

We had two aims. First, we sought to generate a qualitative synthesis of the literature, including any insights from prior coronavirus outbreaks. Second, we sought to perform a systematic review and meta-analysis to estimate the frequency of neurological or HEENT manifestations in patients with COVID-19, and how this may differ from patients without COVID-19. Only English-language results were included. Hand searching was done with reference lists of obtained articles.

Given the broad objectives for the qualitative synthesis portion of the review, we first included any papers reporting on neurological or HEENT manifestations in human populations, capturing not only case series, case–control, and cohort studies but also case reports. AG and IR screened the eligibility of the search results based on the relevance of the title and abstract to the broad review topic. For in-depth systematic review and meta-analysis, we included only those studies that (a) included at least 100 patients with COVID-19 and (b) used a prospective or retrospective cohort, cross-sectional, or case–control design for identifying these manifestations of interest. Studies that passed initial screening were judged suitable for a detailed quality appraisal if they fulfilled these criteria. Any disagreements were to be resolved by consensus between AG and IR, with MV (physician) included if no agreement could be reached.

Data for papers included in the qualitative synthesis were abstracted by AG and IR independently into tables noting the study locations (region/country), study design, number of participants, and key findings. Studies were organized by topic as relating to either (a) HEENT manifestations (including anosmia or dysgeusia), (b) general neurological manifestations (studies that did not focus on a single specific manifestation but rather examined a mix of various symptoms, which varied from nonspecific symptoms like fatigue to headache, weakness, dizziness, or neuropsychiatric symptoms like depression); (c) specific CNS manifestations (such as stroke, encephalopathy, cerebral venous sinus thrombosis, demyelination, seizures, movement disorders, or brain neuroimaging abnormalities); and (d) specific PNS manifestations (such as Guillain–Barre Syndrome (GBS), other cranial or peripheral neuropathies, neuromuscular junction disorders, or myopathy).

The quality of studies deemed acceptable for inclusion in the meta-analysis was assessed by AG and IR using the Quality in Prognosis Studies (QUIPS) tool, which evaluates studies as having low, moderate, or high risk of bias on six domains: study participation, study attrition, prognostic factor measurement, outcome measurement, study confounding, statistical analysis and reporting.^16^

Pooling of studies was done only if more than two cohorts or case–control studies were available, and the pooling was organized by the study topic (e.g. general neurological manifestations, anosmia, etc). An inverse variance weighted method was used to obtain summary proportions or odds-ratios (if control group available) with 95% confidence intervals, using random-effects models with forest plots, and assessment of heterogeneity using I2.^17^ In addition to calculating overall pooled estimates from all available studies that met our criteria for pooling described above, we also grouped studies according to their study design (prospective cohort, retrospective cohort, cross-sectional, case–control) to derive summary estimates from similarly-designed studies. For sets of 10 or more studies of key manifestations comparing patients with COVID-19 to those without COVID-19, we planned to examine for publication bias using funnel plots and the Harbord test (however, as noted below, this threshold was never met). Analyses were performed with STATA-MP 16.1. We used the metaprop_one command to calculate summary proportions of COVID-19 patients with the different manifestations of interest, obtaining the pooled estimate as a weighted average, specifying a random-effects model using the method of DerSimonian and Laird, and obtaining the confidence intervals based on exact binomial procedures.^18^ We used the meta size and meta forest plot commands to obtain odds ratios for comparisons with control patients.

## Results

**Figure 2:**
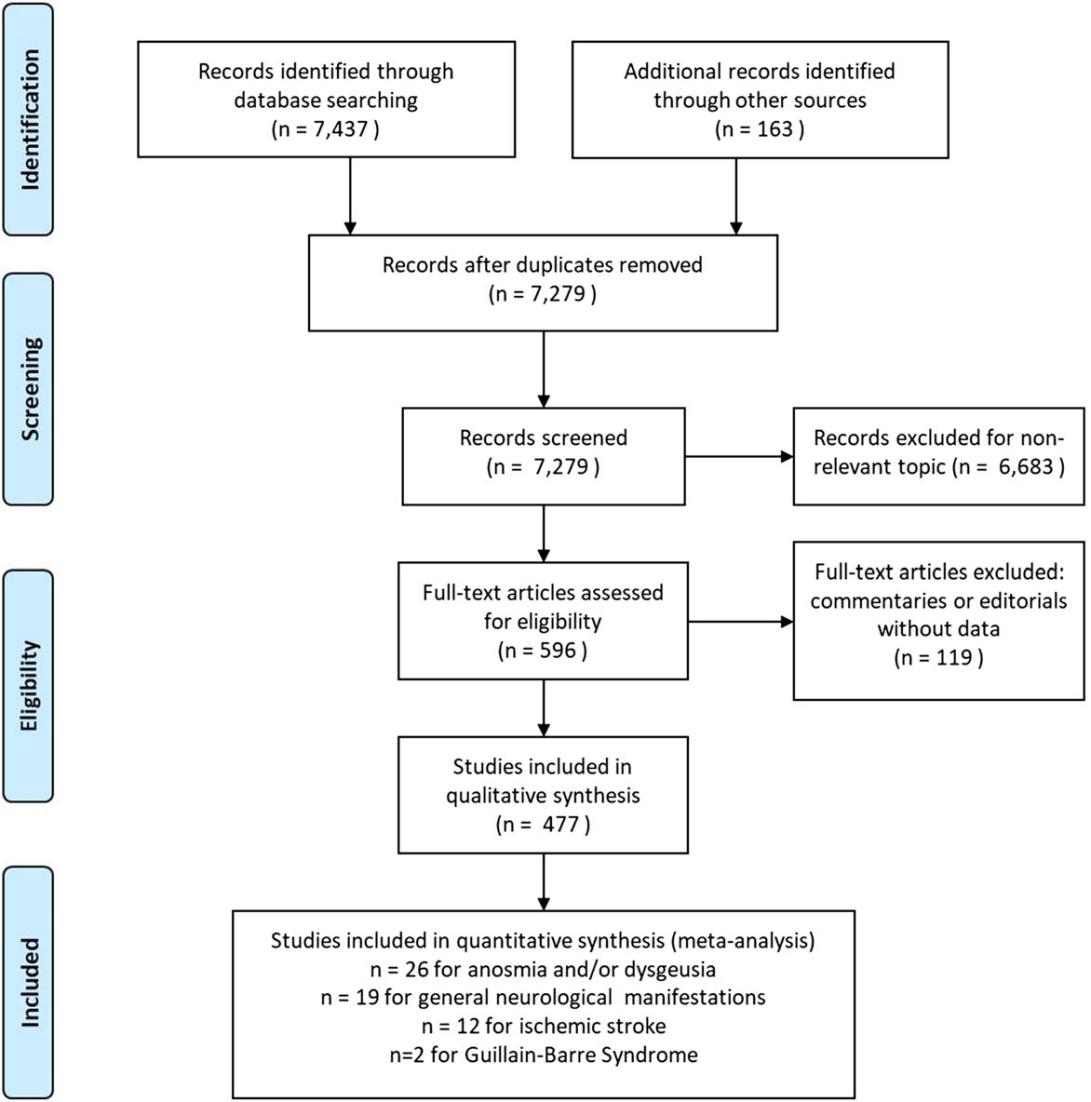
PRISMA flow diagram for our systematic review of neurological and HEENT manifestations in patients with COVID-19.

**Figure 3.**
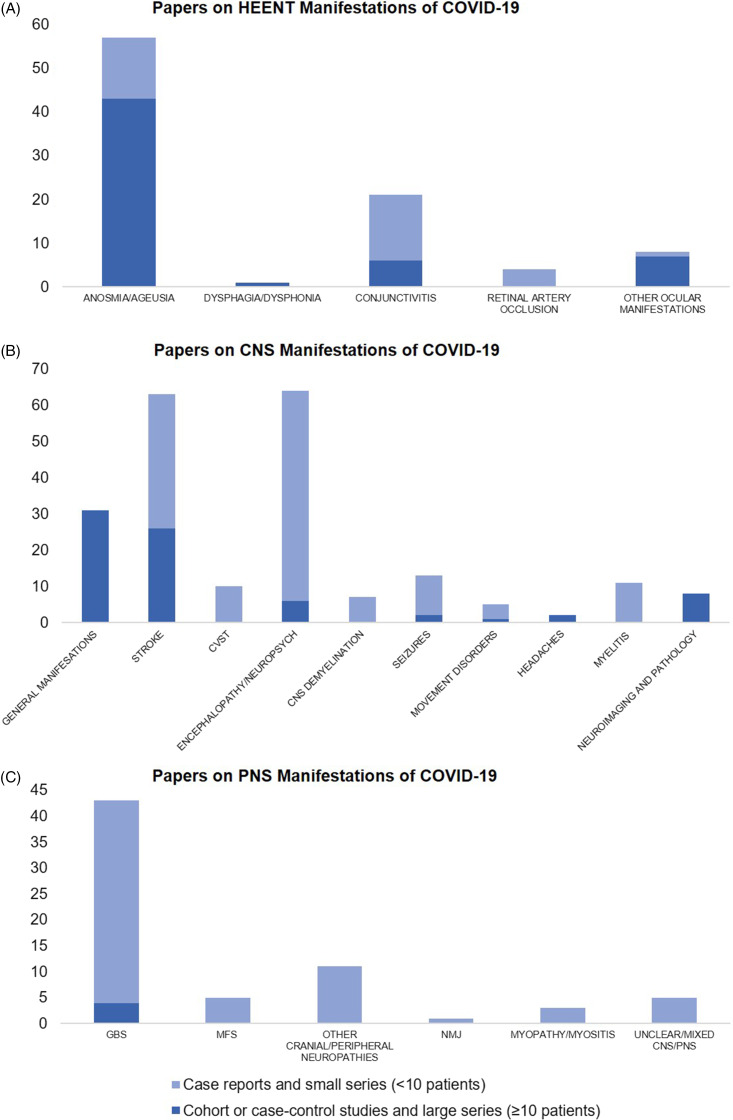
Number of published papers as of March 31, 2021 on (A) head–eyes–ears–nose–throat (HEENT), (B) central nervous system (CNS), and (C) peripheral nervous system (PNS) manifestations of COVID-19. CVST = Cerebral Venous Sinus Thrombosis, GBS = Guillain–Barre Syndrome, MFS = Miller–Fisher Syndrome, NMJ = Neuromuscular Junction disorders like myasthenia gravis.

Our search strategy identified 7279 papers, of which 477 were retained for qualitative synthesis and 59 for meta-analyses (Figure 2). The majority of the included studies (Figure 3) were from the USA (28.6%), China (19.1%), and Italy (12.9%). The mean age of the patients was crudely estimated to be 52.8 years (average of study-reported means). Women accounted for 50.4% of the patients in these studies.

### Potential HEENT Manifestations of COVID-19

We found 91 papers on HEENT manifestations, including 14 prospective cohorts, 7 retrospective cohorts, 5 case–control, and 23 cross-sectional studies, as well as 9 case series with ≥10 patients, the remainder being small case series and case reports (Figure 3A). In total, these publications have described 17,452 patients with COVID-19 and disorders of smell or taste, 188 with dysphagia or dysphonia, 63 with conjunctivitis, 4 with retinal artery occlusion, and 202 with other ocular symptoms like eye pain, photophobia, flashes/floaters, blurry vision, and red eyes (Supplementary Figure 1A). Most of these studies were from China (*n* = 12), Italy (*n* = 7), and France (*n* = 6).

#### Anosmia and Ageusia

There is a growing recognition of sudden anosmia or ageusia as important symptoms of COVID-19 (cohort studies and large series are summarized in Supplementary Table 2, small case series and reports in Supplementary Table 3). Olfactory dysfunction may in some cases be the only symptom of COVID-19.^19^ A cross-sectional nationwide Italian survey found that sudden olfactory loss was the only symptom in 19.2% of COVID-19 confirmed cases.^20^

Furthermore, olfactory dysfunction appeared before other COVID-19 symptoms in 11.8% of patients in one European multicenter study; among the 18.2% of patients without nasal obstruction or rhinorrhea, 79.7% still reported dysosmia.^21^ This suggests that some COVID-related dysosmia may be due to olfactory nerve or bulb dysfunction versus just obstructive symptoms. Nonetheless, upper respiratory tract infections account for 22%–36% of all olfactory loss (“conductive”).^22^ A relevant case report in this regard was that of a COVID-19 patient with new anosmia who was shown to have bilateral obstructive inflammation of the olfactory clefts, likely preventing odorant molecules from reaching the olfactory epithelium.^23^ In the aforementioned multicenter study,^21^ early olfactory recovery was reported by 44.0% of the patients; this is unexpected for olfactory nerve damage which tends to be more persistent.^21^ Additionally, mouse models indicate that SARS-CoV-2 infection of non-neuronal cells types may also result in anosmia and disturbances of odor perception.^24^

However, in a compelling case report, a 25-year-old woman with COVID-19 and severe anosmia and dysgeusia was found to have MRI signal alteration in the posterior gyrus rectus – compatible with a viral invasion of the olfactory cortex and adjacent regions – that then rapidly resolved over the course of a month along with recovery from anosmia.^25^ Although no cerebrospinal fluid (CSF) testing was reported, this case suggests that COVID-19-related anosmia may be associated with transient MRI changes and that rapid recovery does not rule out a neurological contribution. In addition, in an online observational study of patients with olfactory and gustatory alterations secondary to COVID-19 in Spain, 54.0% of patients did not report concomitant nasal congestion or excess mucus, suggesting a nonobstructive cause to their symptoms.^26^ One unifying conclusion from these disparate threads of evidence may be that anosmia in COVID-19 is a spectrum ranging from purely neurotropism-related to obstruction-related pathology, with many cases involving a mix of both.

It is important to note that the duration of olfactory loss varied markedly among studies. In a European multicenter study, 72.8% of patients recovered from olfactory loss after 8 days and 3.4% of patients recovered after 15 days or longer.^21,27^ Meanwhile, a Chinese multicenter study reported that olfactory loss may last up to 95 days or longer,^27^ and an Italian multicenter prospective study reported that 7.2% of patients still had severe dysfunction 60 days after symptom onset.^28^

**Figure 4.**
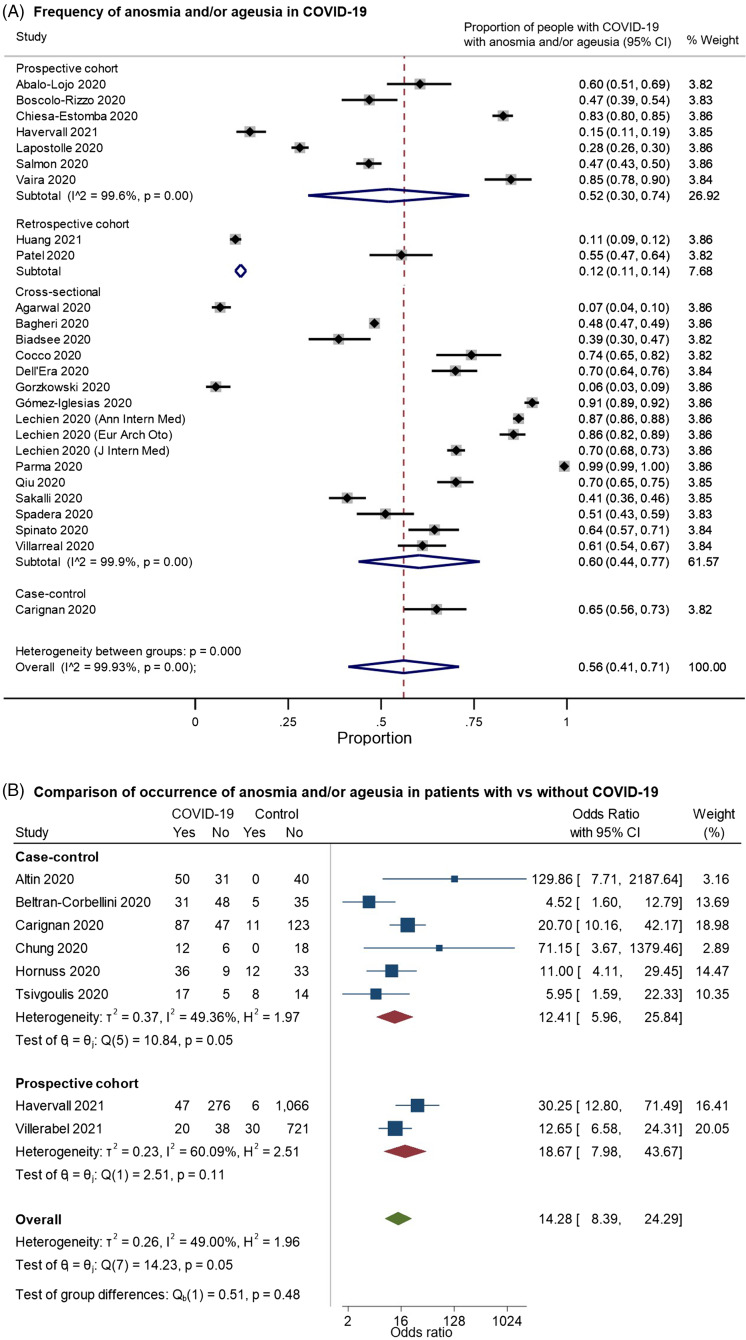
Forest plots from random-effects meta-analyses for studies examining (A) the proportion of patients with COVID-19 who have anosmia and/or dysgeusia (restricted to studies with at least 100 patients) and (B) comparing the occurrence of these symptoms with control groups without COVID-19. The studies are grouped by their design (cross-sectional, case–control, retrospective or prospective cohorts). They included a heterogeneous mix of hospitalized and community-dwelling patients. In Figure 4B, the “Yes” columns indicate the number of patients in a given group (COVID-19 patients or control) who had anosmia and/or ageusia, while “No” indicates the number of patients who did not have this symptom.

We identified 26 studies that provided data on the frequency of anosmia or ageusia among patients with COVID-19 and met our inclusion criteria for pooled analysis (Figure 4). On examining the quality of these studies (Supplementary Figure 2A), most of the studies had a moderate-to-high risk of bias. These largely related to: (a) selection bias in recruitment of either only hospitalized patients well enough to participate or outpatients agreeing to take surveys; (b) reliance on survey-based assessments of olfactory/gustatory complaints, rather than the direct assessment of function; and (c) potential confounding by iatrogenic factors or unmeasured comorbidities. In random-effects meta-analysis, anosmia/ageusia were estimated to occur in 56% of patients with COVID-19, but the estimate varied very widely by study design but also among studies in the same design classifications (overall pooled proportion 0.56, 95% CI: 0.41–0.71, *n* = 26 studies; prospective cohorts only: 0.52, 95% CI: 0.30–0.74, *n* = 7; retrospective cohorts: 0.12, 95% CI: 0.11–0.14, *n* = 2; cross-sectional: 0.60, 95% CI: 0.44–0.77, *n* = 16; case–control: 0.65, 95% CI: 0.56–0.73, *n* = 1; Figure 4A). Indeed, the studies were highly heterogeneous (I2: 99.9%). Eight studies included controls without COVID-19; on pooling these studies, anosmia/ageusia was more common in patients with COVID-19 (overall OR: 14.28, 95% CI: 8.39–24.29, *n* = 8; prospective cohorts: 18.67, 95% CI: 7.98–43.67, *n* = 2; case–control: 12.41, 95% CI: 5.96–25.84, *n* = 6; overall I2: 49.00%, moderate heterogeneity; Figure 4B). Noting that two studies^29,30^ reported no anosmia/ageusia among controls, we performed a sensitivity analysis excluding these studies (Supplementary Figure 3), giving a pooled OR of 12.65 (95% CI: 7.37–21.70, *n* = 6, I2: 54.92%). An important limitation here was the variability in the definition and assessment of control patients – for example, while some studies provided standardized assessments in-person using odor- or taste-based tests to participants without COVID-19,^31,32^ others used standardized questionnaires,^33,34^ while others reported using a mix of questionnaires and smell identification tests.^29,30^

Data are now emerging on longer-term consequences of olfactory/gustatory dysfunction, with some of these patients reporting unpleasant taste or odor (cacosmia or cacogeusia) as they recover from ansomia or ageusia, potentially related to functional alterations in special sensory processing pathways.^35,36^

#### Conjunctivitis and Other Ocular Manifestations

With the conjunctival epithelium being a potential portal of infection for SARS-CoV-2,^37^ conjunctivitis has been reported at variable frequencies of 0.8%–31.6% among hospitalized patients (Supplementary Tables 2–3). Importantly, there has been biological confirmation of conjunctival infection with SARS-CoV-2 RNA in at least nine cases to date – a single patient with conjunctivitis in a Zhejiang hospital-based series,^38^ 2/12 patients with conjunctivitis in a Hubei study,^39^ 1/56 COVID-19 patients in a Hong Kong study,^40^ three other cases in China,^41,42^ and single cases in Canada^43^ and Iran.^44^

It is also worth noting that acute painless monocular vision loss in keeping with ocular or retinal ischemia (retinal artery occlusion) has been reported in three case reports (Supplementary Table 3). Such reports are supportive of the general observation of increased risk of thromboembolic events with COVID-19, as discussed below in the context of stroke.

### Potential CNS Manifestations of COVID-19

In an online survey of 2343 worldwide physicians by the European Academy of Neurology core COVID-19 Task Force, the majority (67.0%) reported having evaluated fewer than 10 patients with neurological manifestations of COVID-19 as of the spring of 2020, with the most frequently reported neurological findings being headache (61.9%), myalgia (50.4%), anosmia (49.2%), ageusia (39.8%), impaired consciousness (29.3%) and psychomotor agitation (26.7%), as well as encephalopathy and acute cerebrovascular disorders (21.0%).^45^ Approaching the spring of 2021, we found 215 papers on various CNS manifestations, including 9 prospective cohorts, 34 retrospective cohorts, 1 case–control, and 13 cross-sectional studies, as well as 17 case series with ≥10 patients; the remainder were small series or case reports (Figure 3B). In total, these publications described 92,838 patients with COVID-19 and general neurological manifestations, 7910 with stroke, 57,721 with encephalopathy or other neuropsychiatric presentations, 283 with headaches (aside from those included in studies of general manifestations), 220 with various neuroimaging/neuropathological abnormalities, 321 with movement disorders (including Parkinsonism, ataxia, and myoclonus), 46 with cerebral venous sinus thrombosis (CVST), 32 with seizures, 11 with myelitis, and 7 with other CNS demyelination (Supplementary Figure 1B).

Neurological manifestations seem to be most commonly recognized in ill patients with multiple other symptoms but can occur at any time during infection. Indeed, many reported CNS manifestations in the literature include nonspecific symptoms seen with other viral infections; for instance, headaches were noted in 8%–70.3% of COVID-19 cases in studies in various countries (key studies summarized in Supplementary Table. 4).^46,47^ In a retrospective series of 217 hospitalized patients in Wuhan, neurological symptoms were reported in 36.4%, including dizziness, headache, impaired consciousness, stroke, ataxia, and seizures, more commonly among those with severe infections (45.5%).^48^ Those with CNS symptoms had lower peripheral blood lymphocyte counts. In a series of 58 consecutive patients with acute respiratory distress syndrome (ARDS) due to COVID-19 in Strasbourg, corticospinal tract findings such as hyperreflexia, clonus, and extensor plantar responses were seen in 67% of patients, highlighting the potential for neurological involvement even in the absence of patient-reported symptoms.^49^ That being said, it is unclear whether these patients were specifically asked about various neurological symptoms. In a study of 404 consecutive COVID-19 patients in Washington State, 208 (51.5%) were reported to present with CNS symptoms including altered mental status (21.3%), headache (20.3%), and dizziness (7.7%), with 57.0% of those with altered mental status having preexisting dementia.^50^ A 6-month follow-up study of patients with COVID-19 discharged from a hospital in Wuhan reported that around 63% of survivors were troubled by fatigue or muscle weakness (described in combination as a single symptom), sleep difficulties, and anxiety or depression (all fairly nonspecific findings). An initial analysis from TriNetX, a multinational collaborative research platform that published data on 40,469 patients with COVID-19, reported that 9086 (22.5%) had various neuropsychiatric manifestations.^51^ This was followed recently by another TriNetX analysis, this time of 236,379 patients – including 190,077 patients who were not hospitalized and 8945 patients who needed critical care – which is the largest dataset to date.^52^ This study was limited by its retrospective design and reliance on electronic medical record codes. However, the study had a large sample size, and included both hospitalized and nonhospitalized patients. There was a 6-month follow-up of survivors, and a robust matched control population of patients with influenza and other respiratory tract infections (*n* = 236,038). The estimated incidence of a neurological/psychiatric diagnosis in the 6 months after COVID-19 in this study was 33.6%, with 12.8% receiving their first such lifetime diagnosis; it is unclear to what extent the remaining patients had preexisting chronic neurological/psychiatric conditions versus previously resolved complaints. The incidence was higher in patients requiring critical care (46.4% for any manifestation). The leading manifestations overall were 2.10% for ischemic stroke (0.6% intracranial hemorrhage), 0.67% for dementia, 1.40% for psychosis, 0.11% for Parkinsonism, and 17.39% for anxiety disorder. The majority of these diagnoses were more common in patients with COVID-19 than in those with influenza (HR: 1.44, 95% CI: 1.40–1.47) or other respiratory tract infections (HR: 1.16, 95% CI: 1.14–1.17).

**Figure 5.**
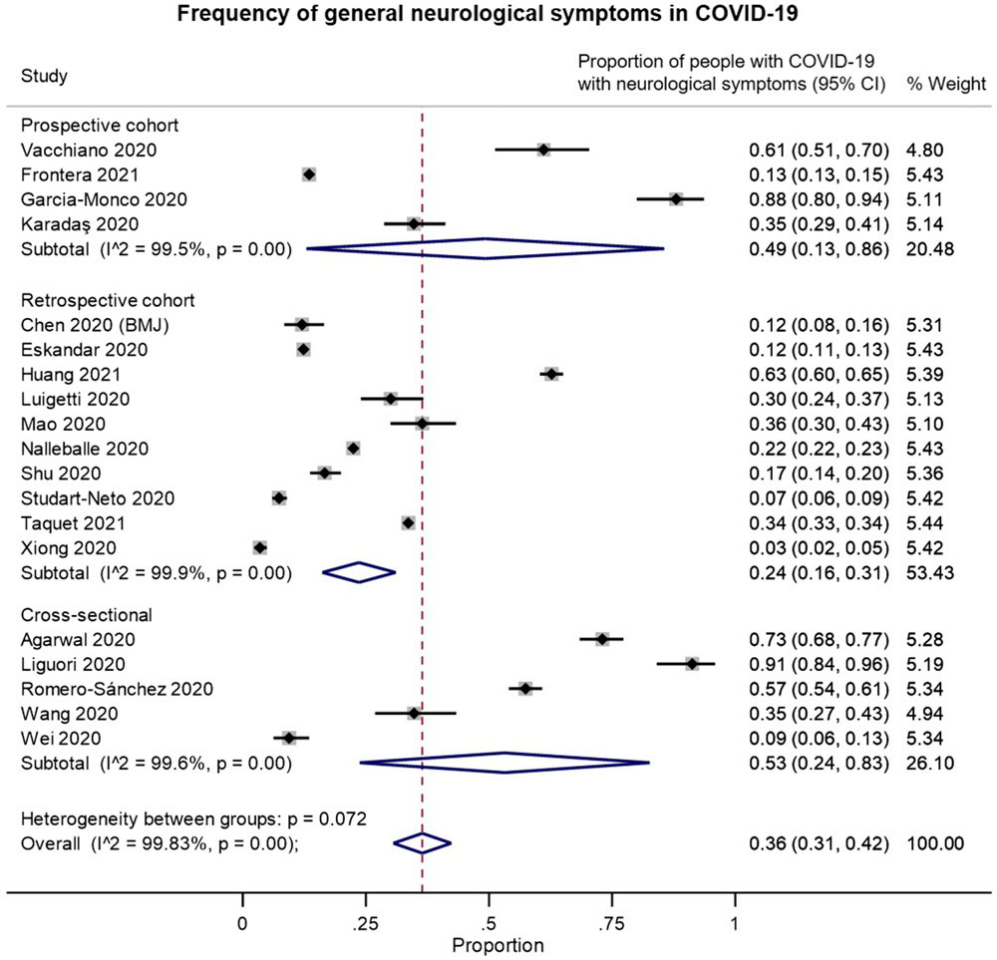
Forest plots from random-effects meta-analyses for studies examining the proportion of patients with COVID-19 who have general neurological symptoms that met inclusion criteria for pooled analysis, grouped by study design. These studies generally only included hospitalized patients.

We identified 19 studies that provided data on the frequency of neurological symptoms among patients with COVID-19 and met our inclusion criteria for pooled analysis. On examining the quality of these studies (Supplementary Figure 2B), most of the studies had a moderate-to-high risk of bias. These biases were largely related to: (a) selection bias in recruitment of only hospitalized or critically ill patients; (b) reliance on neurological diagnostic codes in electronic records or on the presence of neurological investigations or consultations to identify “positive” outcomes; and/or (c) potential confounding by iatrogenic factors or unmeasured comorbidities. In random-effects meta-analysis, neurological symptoms were estimated to occur in 36% of (hospitalized) patients with COVID-19 (overall pooled proportion: 0.36, 95% CI: 0.31–0.42, *n* = 19; prospective cohorts: 0.49, 95% CI: 0.13–0.86, *n* = 4; retrospective cohorts: 0.24, 95% CI: 0.16–0.31, *n* = 10; cross-sectional: 0.53, 95% CI: 0.24–0.83, *n* = 5; Figure 5). However, the studies were highly heterogeneous (I2: 99.8%).

In thinking about why CNS symptoms seem to occur in COVID-19 at a higher frequency than typical respiratory infections, it is worth noting that markers of astrocytic and neuronal injury have been found in patients with COVID-19. A study of plasma samples in patients with mild (*n* = 20), moderate (*n* = 9), or severe (*n* = 18) COVID-19, collected at presentation and again an average of 11.4 days later, found that patients with moderate and severe COVID-19 had higher plasma concentrations of GFAP (Glial Fibrillary Acidic Protein, a marker of astrocytic activation/injury) than age-matched controls, while NfL (neurofilament light chain, a marker of intra-axonal neuronal injury) was also increased with severe disease.^53^ Whereas GFAP showed an early peak in severe disease, NfL showed a sustained increase from the first to last follow-up, potentially reflecting a sequence of early astrocytic response versus more delayed axonal injury.

#### Stroke

Stroke, including large vessel involvement in young patients, has been reported in many studies as a presenting symptom of COVID-19 (Supplementary Table 5). In a study from Wuhan, China, stroke was reported in 5.7% of those with severe COVID-19 versus 0.8% with non-severe COVID-19, which is an unexpectedly high rate.^48^ In another study from Strasbourg, France, two asymptomatic small acute ischemic strokes and one subacute ischemic stroke were identified among 13 patients (15.4%) who underwent MRI for encephalopathic symptoms.^49^ However, subsequent cohorts and registries have reported far less impressive rates of stroke. For example, stroke occurred in only 3(0.7%) of 404 consecutive patients hospitalized with COVID-19 in Washington State, of which one was a hemorrhagic stroke.^50^ In a retrospective cohort study at two academic hospitals in New York City, 1.6% of 1916 patients with COVID-19 had an acute ischemic stroke, although this was much higher than the rate of 0.2% seen among 1486 patients in a comparison cohort with influenza (OR: 7.6, 95% CI: 2.3–25.2) even after adjustment for vascular risk factors, viral symptomatology, and critical care.^54^ Similarly, in the Society of Vascular and Interventional Neurology (SVIN) COVID-19 Multinational Registry, 1.1% of 14,483 patients with laboratory-confirmed COVID-19 had an acute ischemic stroke.^55^ A large multicenter, multi-national observational study with 26,175 hospitalized patients from 11 countries (USA, Canada, Brazil, Greece, Italy, Finland, Turkey, Lebanon, Iran, India, and New Zealand) found that 156(0.9%) had a stroke, with 79% of these being ischemic strokes.^56^ In a prediction model using 17,799 patients, the overall stroke risk was estimated to be 0.5% among all centers, with the need for mechanical ventilation and the presence of ischemic heart disease being predictive of stroke. These rates are quite similar to that expected in other causes of critical illness; for example, about 0.5% of patients hospitalized with sepsis have a stroke within 1 year,^57^ whereas 6% of those with severe sepsis have new-onset atrial fibrillation leading to in-hospital stroke in 2.6%.^58^ Indeed, the occurrence of stroke may best be interpreted as a marker of severe COVID-19; in-hospital mortality for COVID-associated stroke was 38.1% in the SVIN registry.^55^ However, in the largest dataset to date from the aforementioned TriNetX platform (236,379 COVID-19 survivors vs. 236,038 controls with other respiratory infections), including hospitalized and nonhospitalized patients, the 6-month incidence of ischemic stroke was 2.10% in COVID-19 patients, with a hazard ratio of 1.45 (95% CI:1.36–1.55) versus controls.^52^

**Figure 6.**
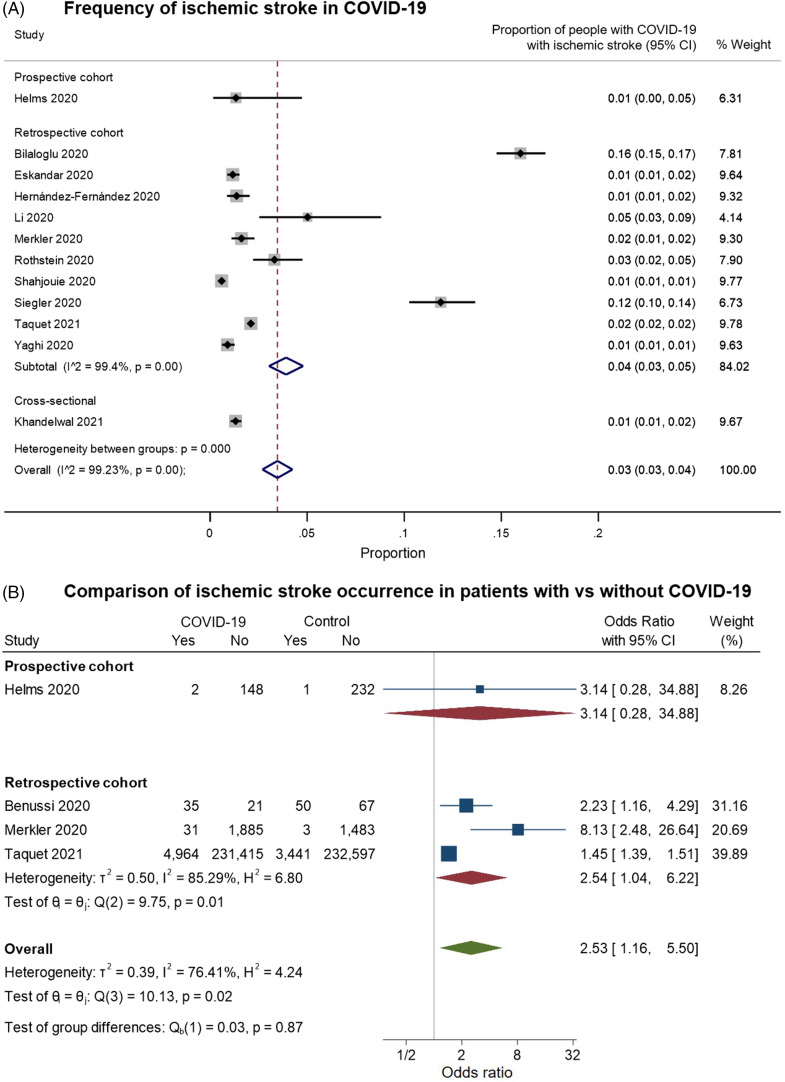
Forest plots from random-effects meta-analyses for studies examining (A) the proportion of patients with COVID-19 who have an ischemic stroke and (B) comparing the occurrence of ischemic stroke in COVID-19 with control groups without COVID-19. The studies are grouped by their design. These studies generally only included hospitalized patients. In Figure 6B, the “Yes” columns indicate the number of patients in a given group (COVID-19 patients or control) who had an ischemic stroke, while “No” indicates the number of patients who did not have this symptom.

We identified 12 studies providing data on the frequency of ischemic stroke among patients with COVID-19 and meeting our inclusion criteria for pooled analysis (Figure 6). On examining the quality of these studies (Supplementary Figure 2C), most of the studies had a moderate risk of bias. These were largely related to: (a) selection bias in the inclusion of only hospitalized or critically ill patients; (b) reliance on stroke diagnostic codes in electronic records or on the presence of neuroimaging investigations to identify “positive” outcomes; and/or (c) the absence of control groups of hospitalized COVID-free patients for comparison (with rare exceptions as noted above). In random-effects meta-analysis, ischemic stroke was estimated to occur in around 3% of (hospitalized) patients with COVID-19 (overall pooled proportion: 0.03, 95% CI: 0.03–0.04, *n* = 12; prospective cohort: 0.01, 95% CI: 0.00–0.05, *n* = 1; retrospective cohorts: 0.04, 95% CI: 0.03–0.05, *n* = 10; cross-sectional: 0.01, 95% CI: 0.01–0.02, *n* = 1; Figure 6A). Again, the studies were highly heterogeneous (I2: 99.2%). Four studies included controls without COVID-19; on pooling these, ischemic stroke was more common among patients with COVID-19 (overall OR: 2.53, 95% CI: 1.16–5.50, *n* = 4; prospective cohort: 3.14, 95% CI: 0.28–34.88, *n* = 1; retrospective cohorts: 2.54, 95% CI: 1.04–6.22, *n* = 3; I2: 76.41%, moderate heterogeneity, Figure 6B).

There is also an indication that stroke occurring with COVID-19 may more commonly involve the large vessels than other causes of stroke. Small case series from Spain and the USA have highlighted the occurrence of non-atherosclerotic large artery ischemic stroke in COVID-19,^59,60^ and a case–control study of 329 patients reported large vessel occlusions (LVOs) in 31.7% of patients with COVID-19-associated stroke versus only 13.8% of patients with stroke but without COVID-19.^61^ This observation was validated by an analysis of 41,971 patients with acute ischemic stroke in the Get With The Guidelines-Stroke database, which found that patients with COVID-19 were more likely to have LVOs and more severe stroke, in addition to being younger.^62^ The Black population seems to be disproportionately represented among patients with COVID-associated stroke in the USA;^62,63^ this community has also been disproportionately affected by the pandemic on all fronts.^64^

While few studies have reported the underlying etiologies of stroke in the setting of COVID-19 in any detail, two studies have suggested that cryptogenic etiologies may be more common in patients with ischemic stroke in the context of COVID-19 compared to stroke without COVID-19.^65,66^ Endothelial dysfunction is thought to underlie the heightened risk of stroke after recent viral or respiratory infections.^67^ Endothelial cell infection and endotheliitis have been demonstrated in different vascular beds in patients with COVID-19.^68^ There are also reports of stroke associated with coagulopathies, suggesting an additional underlying mechanism (Supplementary Table 6). About 20%–55% of patients with COVID-19 have laboratory evidence of coagulopathy, which generally appears prothrombotic, with elevated D-dimer concentration particularly associated with poor outcomes.^69^ Additionally, a single-center cohort study in New York found an increased incidence of lupus anticoagulant positivity as well as an association between lupus anticoagulant positivity and incidence of thrombosis in patients with COVID-19.^70^

#### Encephalopathy, Encephalitis, and Neuropsychiatric Presentations

Encephalopathy and/or encephalitis merit special mention. In the Wuhan series, the most common CNS symptom was loosely defined “impaired consciousness”, reported in 14.8% with severe COVID-19 versus 2.4% with non-severe COVID-19.^48^ In a retrospective report of 113 deceased patients in Wuhan, encephalopathy or altered level of consciousness lasting more than 24 h was recorded in one-fifth of the patients.^71^ Seizures may also occur rarely in some patients; for example, 2 (0.5%) of 404 consecutive patients hospitalized with COVID-19 in Washington State had seizures.^50,72–79^ In the Strasbourg series, agitation was reported in 69%, and confusion in 65% of 40 patients evaluated using the CAM-ICU (Confusion Assessment Method for the Intensive Care Unit).^49^ Among 13 of these patients who underwent brain MRI for “unexplained encephalopathy’”, leptomeningeal enhancement was seen in eight, and bilateral frontotemporal hypoperfusion was seen in all 11 who received perfusion imaging. Of eight patients who underwent electroencephalography (EEG), one showed diffuse bifrontal slowing. CSF was tested in seven patients: there were no cells in any of these patients, and reverse transcriptase polymerase chain reaction (RT-PCR) was negative for SARS-CoV-2, suggesting that these symptoms were not due to direct viral invasion of the CNS. There was also the intriguing finding of persistent neuropsychological impairment in the form of a “dysexecutive syndrome”, reported in 33% of discharged patients in this series, described as a combination of inattention, disorientation, and poorly organized movements to command.^49^

There have been a few other case reports of encephalopathy or encephalitis as an early clinical feature of COVID-19 (Supplementary Table 6), including at least one case of limbic encephalitis.^80^ Several cases have also been reported with concomitant demyelination-like changes on MRI in keeping with acute disseminated encephalomyelitis (ADEM).^81–84^ Particularly in the absence of CSF testing or neuroimaging findings, it can be challenging to decide whether a given patient has an encephalitis or just a delirium, the latter being common in critically ill patients. 73.6% of 243 consecutive critically ill patients with COVID-19 patients had delirium for a median of 5 days, with a median score of 6 on the CAM-ICU assessment in keeping with severe delirium.^85^ As in other situations, delirium in COVID-19 is a harbinger of mortality; in the aforementioned study, critically ill COVID-19 patients with delirium had a mortality of 26.4% versus 15.8% for those without delirium.^85^ Psychiatric presentations may also occur frequently in the context of COVID-19, with an association reported between baseline immune response and subsequent manifestations of depression and anxiety disorders in an Italian study.^86^

It is important to emphasize that most COVID-19 cases with CNS manifestations have not demonstrated CNS infection. A Swiss study did not find SARS-CoV-2 RNA in any of the CSF samples from 31 COVID-19 patients with such manifestations, but found signs of blood-brain barrier disruption, which could have been precipitated by SARS-CoV-2.^87^ However, there have been at least nine cases of encephalopathy/encephalitis or other CNS symptoms with evidence of SARS-CoV-2 infection in the CSF, including one from Beijing,^88^ one from Iran (with cerebellitis),^89^ one from Sweden,^90^ one from Spain,^91^ one from Italy (that was debatably diagnosed as ADEM),^92^ one from Germany (with meningitis),^93^ two from France (antibodies detected),^94^ and one patient from Brazil with white-matter imaging changes and sensorimotor symptoms.^95^ Importantly, the patient from Sweden, who had acute necrotizing encephalopathy, was found to have SARS-CoV-2 in the CSF 19 days after symptom onset and had tested negative twice, indicating the potential value of repeated CSF analysis in patients with neurological manifestations of COVID-19.^90^ In another series of six patients with COVID-19 in Gothenburg who had undergone lumbar punctures for neurological complaints (primarily encephalopathy), SARS-CoV-2 RNA was detected in the CSF at low levels in three patients in one but not in a second PCR assay, suggesting issues with the reproducibility of some of these findings.^96^ Furthermore, it is important to be vigilant for coinfections with other agents; for example, a case of COVID-19 with new tuberculosis meningitis has been reported.^97^ Furthermore, even if SARS-CoV-2 is found in the brain, that does not necessarily mean it caused neurological damage. For instance, the most compelling neuropathological study to date came from a postmortem case series in Hamburg,^98^ in which SARS-CoV-2 was detected in the brains of 21(53%) of 40 examined patients, with SARS-CoV-2 viral proteins found in cranial nerves originating from the lower brainstem and in isolated cells of the brainstem. However, the presence of SARS-CoV-2 in the CNS was not associated with the severity of neuropathological changes, with neuroinflammatory changes in the brainstem seen in most cases even without detected SARS-CoV-2. Consequently, the authors concluded that there was no evidence for CNS damage directly caused by SARS-CoV-2.

#### Neuroimaging Patterns

Neuroimaging patterns have been investigated in various case reports and series to better understand the neurotropic nature of COVID-19. Notably, a case series of 37 severe COVID-19 patients with neurological manifestations who underwent brain MRI found that 43% had signal abnormalities in the medial temporal lobe, 30% had non-confluent multifocal white matter hyperintense lesions on T2-weighted and diffusion sequences, and 24% had extensive and isolated white matter microbleeds.^99^ Multiple lobes may be affected; in a series of four patients in Milan with subacute encephalopathy, multifocal involvement was seen on MRI in the parietal, occipital and frontal lobes in all cases.^100^

Another series of four COVID-19 patients with abnormal mental status reported a common MRI pattern of multifocal subcortical/cortical petechial-type hemorrhages, suggesting a thrombotic microangiopathy.^101^ A series of 9 patients presenting with delayed recovery of consciousness or agitation also reported microbleeds, but with a specific predilection for the corpus callosum^102^. Of particular relevance is a retrospective chart review of 115 critically ill COVID-19 patients in New York City who had brain MRIs, which found that 30.4% had leukoencephalopathy and/or cerebral microbleeds.^103^ It appears that these neuroimaging findings may serve as a prognostic marker for severe COVID-19. For example, in the New York City study, patients with leukoencephalopathy and/or cerebral microbleeds faced worse complications like moderate-severe acute respiratory distress syndrome (88.6% vs. 23.8%), requiring longer ventilator support (34.6 vs. 9.1 days), with higher mortality (20% vs. 9%).^103^

### Potential PNS Manifestations of COVID-19

Nerve root, plexus, peripheral nerve (including cranial nerve), and muscle involvement have all been reported with COVID-19.^104^ We found 69 papers on PNS manifestations, four of which were retrospective cohort or registry studies, the rest being small case series or case reports of overall low methodological quality (Figure 3C). In total, these publications described 309 patients with GBS, 10 with Miller–Fisher Syndrome, 14 with other cranial or peripheral neuropathies, 3 with neuromuscular junction disorders, 8 with myopathy or myositis, and 9 with unclear or mixed CNS-PNS presentations (Supplementary. Figure. 1C).

Muscle involvement can be quite nonspecific in the form of myalgia or muscle fatigue, seen in 44%–70% of COVID-19 patients in various series and cohorts (Supplementary Tables 2–4), with increased CK levels seen in about a third of admitted patients.^46,105^ In Wuhan, symptoms attributed to “skeletal muscle injury” were reported in 19.3% of patients with severe COVID-19 versus 4.8% with non-severe COVID-19; patients with such symptoms had lower lymphocyte counts and higher CRP levels than those who did not.^48^

#### Guillain–Barré Syndrome

Acute inflammatory demyelinating polyradiculoneuropathy or GBS and its variants have now been described in association with COVID-19 in four retrospective studies (Supplementary Table 7) and several case reports (Supplementary Table 8). An increase in GBS has been reported in some centers during the pandemic;^106^ for example, hospitals in Lombardy and Veneto (northern Italy) reported a 2.6-fold increase in the incidence of GBS in March–April 2020 compared to March–April 2019.^107^ However, a national-level study in the United Kingdom (UK) found that the incidence of GBS during March–May 2020 had fallen compared to GBS cases reported during the same time-periods in 2016–2019.^108^

There is much speculation on the pathophysiologic mechanism of GBS and other PNS manifestations. In the aforementioned French center, the SARS-CoV-2 nasopharyngeal swab and serology were negative in six of the patients with GBS, casting doubt on the contributory role of COVID-19 to their apparent increase in cases.^106^ A case report from Italy also suggested that a para-infectious rather than postinfectious mechanism as the PNS and respiratory symptoms progressed in tandem with each other,^109^ whereas another patient from Switzerland had a positive nasopharyngeal swab for SARS-CoV-2 preceding the first signs of polyneuropathy.^110^ Among the cases reported from Madrid^54^, Italy,^109,111–115^, Morocco,^116^ Turkey,^117^ Spain,^118^ USA,^119,120^ the Netherlands,^121^ Switzerland,^122^ Iran,^123^ and Germany,^124^ none had detectable SARS-CoV-2 in their CSF, arguing against direct neuroinvasion as the underlying mechanism of these presentations, and overall favoring a postinfectious or immune-mediated process. Recent translational work suggests that molecular mimicry between the SARS-CoV-2 virus and human heat shock proteins 90 and 60 – associated autoimmune diseases including GBS – may be an important contributor to the pathophysiology.^122^ This is in contrast to a recent study that searched for homology between the SARS-CoV-2 and human genome and proteome, and concluded that SARS-CoV-2 contains no additional immunogenic material known or proven to drive GBS.^108^

It is worth noting that at the present time, the incidence of SARS-CoV-2-related GBS does not seem to be as high as that of well-known GBS-associated pathogens like *Campylobacter jejuni* (˜1 in 1000 cases) or the Zika virus (˜1 in 4000 cases).^125,126^ However, when compared to patients without COVID-19 seen during the pandemic, the frequency of GBS was considerably higher among patients with COVID-19 (0.15% vs. 0.02%, standardized incidence 9.44 vs. 0.69 cases/100,000 inhabitant years) in a large retrospective case–control study from Spain.^127^ Similar findings were reported in a recent large analysis of the TriNetX platform, with an estimated 6-month incidence of GBS of 0.08% in patients with COVID-19, with a hazard ratio of 2.06 (95% CI: 1.43–2.96) versus patients with other respiratory tract infections.^52^

**Figure 7.**
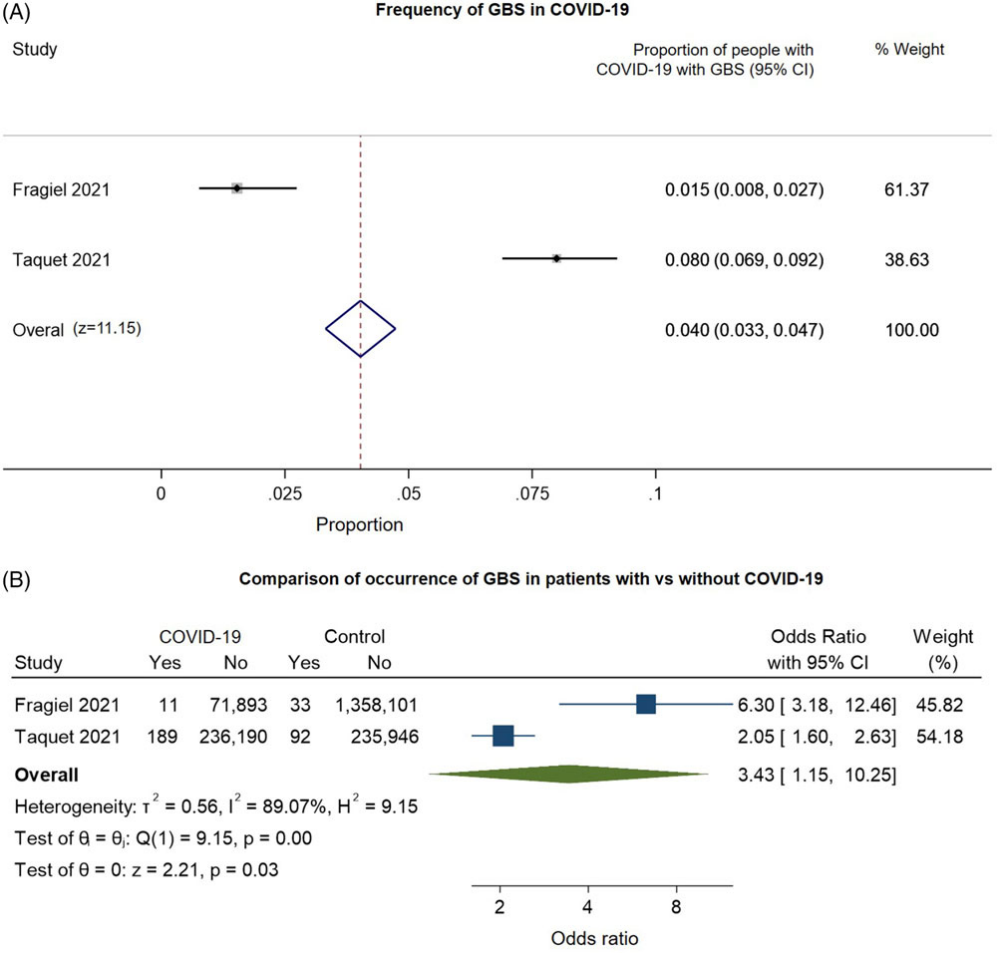
Forest plots from random-effects meta-analyses for two retrospective cohort studies examining (A) the proportion of patients with COVID-19 who develop Guillain–Barre Syndrome (GBS) and (B) comparing the occurrence of GBS in COVID-19 with control groups without COVID-19. In Figure 7B, the “Yes” columns indicate the number of patients in a given group (COVID-19 patients or control) who had GBS, while “No” indicates the number of patients who did not have this symptom.

Two retrospective cohort studies on the frequency of GBS met our inclusion criteria for pooled analysis (Figure 7). These studies had a moderate risk of bias on most domains (Supplementary Figure 2D). These largely related to: (a) concerns of selection bias and (b) reliance on diagnostic codes in electronic records to identify “positive” outcomes. In random-effects meta-analysis, GBS was estimated to occur in around 0.04% of patients with COVID-19 (95% CI: 0.033–0.047%, *n* = 2, Figure 7A). Both studies included controls without COVID-19; GBS was more common among patients with COVID-19 (OR: 3.43, 95% CI: 1.15–10.25, I2: 89.07%, substantial heterogeneity, Figure 7B). However, this calculation was based only on two studies and should be interpreted cautiously.

### Neurological and HEENT Manifestations in the Pediatric Population

There is a relative paucity of data about HEENT or neurological manifestations in children with COVID-19. We found 16 studies of such manifestations in children, including one retrospective cohort study and three case series with ≥10 patients; the remainder was small series and case reports (Supplementary. Figure. 4). In total, these publications described 845 patients with general neurological symptoms, eight with stroke and/or coagulopathy, four with seizures, two with movement disorders, and one with GBS (cohort and large case series are shown in Supplementary. Table. 9, small case series and case reports in Supplementary. Table. 10).

The most compelling HEENT data have come from a cross-sectional study of ocular manifestations in 216 pediatric patients in Wuhan, of whom 22.7% showed various ocular manifestations, mostly consisting of conjunctival involvement.^128^ Compelling neurological data came from a case series of 27 children in London who presented with new CNS and PNS symptoms and MRI changes in the splenium of the corpus callosum that resolved.^129^

Cytokine storms – known to cause acute necrotizing encephalitis of childhood – were reported in critically ill children with COVID-19 in Wuhan,^130^ raising a red flag for potential multisystem autoimmune manifestations. Subsequently, reports emerged of life-threatening neurological involvement in children who developed a rare, hyperinflammatory, severe illness temporally associated with COVID-19, thought to be postinfectious, termed multisystem inflammatory syndrome in children (MIS-C).^131^ This was followed recently by a large case series of 539 children with MIS-C and 577 with severe COVID-19 from 66 hospitals in the USA. Headache or altered mental status was seen at presentation in 40.4% of patients with MIS-C and 32.2% of the children with severe COVID-19. In a related analysis by the same group, 22% of 1695 hospitalized pediatric or adolescent patients with acute COVID-19 or MIS-C were found to have neurological involvement. These were transient in 88% of cases, but 12% of patients developed life-threatening neurological disorders, including severe encephalopathy with splenial lesions, stroke, CNS demyelination, acute fulminant cerebral edema, and GBS. 26% of the patients with life-threatening disorders died, and 40% survived with new neurological sequelae.^132^ There are no high-quality cohort studies of the long-term consequences of COVID-19 or associated neurological involvement in children, particularly with respect to neurodevelopmental outcomes.

## Discussion

In this systematic review, we found a wealth of literature that has emerged over just this past year on neurological and HEENT manifestations of COVID-19. In meta-analyses, we estimated that anosmia/ageusia occurs in around 56% of patients with COVID-19, with about 13-fold higher odds than in patients without COVID-19. We also estimated that neurological symptoms occur in 36% of hospitalized patients, and ischemic stroke in around 3%, the latter being about 2.5-fold more common in hospitalized patients with COVID-19 compared to those without COVID-19. We estimated that GBS occurs in around 0.04% of COVID-19 patients, about threefold more commonly than in patients without COVID-19, although this estimate was based only on two studies.

The accumulated evidence on these manifestations should alert clinicians on the frontlines to maintain a high index of suspicion for COVID-19 even when patients do not have other typical symptoms of the disease, particularly if they have a concerning exposure history, a positive close contact, or are otherwise at high epidemiological risk. We should test such patients for COVID-19, since the neurological or HEENT symptoms may be the very first symptoms. In addition, we should also be vigilant for the development of such presentations in patients with known COVID-19. While it is likely neither feasible nor advisable from a safety standpoint to perform a detailed HEENT or neurological evaluation in every patient with COVID-19,^133^ we should keep a low threshold to perform such evaluations when patients report relevant symptoms – a point worth emphasizing in this era of increasing virtual or telephone visits. This is particularly relevant for manifestations like ischemic stroke and GBS, which will need their own urgent, targeted workup and management independent of supportive care for their COVID-19. Furthermore, these atypical manifestations of COVID-19 underscore the importance of following contact and droplet precautions routinely when interacting with patients during the pandemic, regardless of their presenting complaints. An example of such a strategy is the implementation of “protected code stroke” protocols.^134^

Although various mechanisms have been proposed for the wide range of neurological and HEENT presentations reported with COVID-19, it is crucial to note that several potential explanations do not necessarily implicate the virus itself – for example, many symptoms may simply be consequences of critical illness or of a para- or postinfectious inflammatory response. Given that many of the reported “neurological” symptoms have actually been rather nonspecific, it is also important to emphasize the possibility that many such symptoms may not even be related to COVID-19. As discussed above, few cases have shown evidence of direct neuroinvasion of the virus, and even when it has been detected in brain tissue (as in the Hamburg postmortem series),^98^ it is not convincingly localized to regions of inflammation. The situation is further complicated by limitations of current testing methods – this is especially the case when relying on positive serum IgG or IgM antibody tests as opposed to actual detection of the virus, as has been the case in some studies of anosmia, for example.^135^ There are many more uncertainties with the sensitivity and specificity of antibody tests, increasing the chance of error when associating neurological manifestations with COVID-19.

Our review has important limitations. We only included English-language studies for convenience, given the wide scope of the review. As there were fewer than 10 studies in each pooled set of case–control comparisons, we could not reliably assess for publication bias. Our meta-analysis of GBS in the setting of COVID-19 was especially limited by the inclusion of only two studies; importantly, other studies have not found an increased incidence of GBS during the pandemic.^108^ There was moderate-to-high heterogeneity among the studies in our meta-analysis, and most of the studies in the meta-analysis had a moderate-to-high risk of bias, which means that our pooled estimates should be interpreted cautiously. Setting aside the uncertain origin of the reported symptoms, data to date have been mostly derived by retrospective chart review at best, and patients have not been systematically assessed or questioned about neurological symptoms. Selection bias is a major concern. Studies have generally only reported neurological findings in hospitalized patients; the prevalence of neurological symptoms among community-dwelling patients with milder COVID-19 is likely to be lower, and the spectrum of symptoms (when present) may be quite different. Whereas studies of olfactory or gustatory disorders have been more successful at sampling community-dwelling patients, most of them have relied on crude questionnaire-based assessments rather than directly administered tests of smell or taste. Studies of neurological symptoms have relied excessively on diagnostic codes in electronic medical records (which may be incomplete or inaccurate), or on the availability of neuroimaging or other neurological investigations, which has likely resulted in an underestimation of the frequency and range of such presentations even among hospitalized patients. However, the absence of precise case definitions in most of these studies also limits our ability to distinguish nonspecific complications of severe illness (like hypoxic encephalopathy or critical illness polyneuropathy) from those potentially caused directly or indirectly by SARS-CoV-2 (like encephalitis, hypercoagulable states, and GBS). The World Health Organization has proposed provisional case definitions for the association of COVID-19 with neurological disease (with cases classified as Confirmed, Probable, or Possible based on the strength of the clinical evidence).^136^ Unfortunately, most cases and cohorts to date have not used these criteria to adjudicate the classification of neurological manifestations. Importantly, control groups in these studies have been very variably defined and were not always examined or assessed in a standardized fashion, potentially resulting in inaccurate data. For example, controls who otherwise feel well may not self-report complaints like olfactory or gustatory dysfunction. This can certainly result in an underestimation of symptom occurrence in the control groups, falsely inflating odds ratios for the COVID-19 group.

Indeed, several unanswered questions remain (Box 1). It is unclear to what extent (and why) some manifestations differ in men and women; for instance, women seem to report olfactory dysfunction more often (53% vs. 25% in one study).^21,137^ Whereas several studies have examined the presence of individual HEENT symptoms like anosmia or dysgeusia, and individual neurological symptoms, the co-occurrence of these symptoms remains to be systematically studied. It should be noted that while olfaction may itself be considered a neurological symptom, we considered it a HEENT symptom in our paper, given that it may be explained by obstructive rather than neurological issues. Furthermore, the natural history of these symptoms is also unclear. Based on prior neurocritical literature, we may anticipate some neuropsychiatric symptoms persisting for several months or more.^138^ For example, meta-analyses of delirium among ICU patients with various conditions have reported persistent neurocognitive deficits up to 18 months post-discharge^139^ including mild cognitive impairment.^140^ The concept of “long COVID” has gained much attention, with some patients reporting persistent neurological manifestations ranging from headaches, hyposmia, hypogeusia, and fatigue to more severe manifestations like sleep disorders, pain, cognitive impairment, and sequelae of GBS.^141^ Retrospective cohort data on symptoms at 6 months post-COVID-19 have been provided by recent studies from Wuhan^142^ and the TriNetX database,^52^ but the frequencies and associated phenotypes remain to be elucidated.^141^ High-quality prospective cohorts with harmonized, interdisciplinary data collection are needed to address these enduring questions.

BOX 1. Unanswered Questions

- Is SARS-CoV-2 truly neurotropic or are the observed presentations simply the result of critical illness or other downstream events?
- What is the true incidence of neurological and HEENT presentations among all patients with COVID-19?
- How often do HEENT symptoms involving vision, hearing, smell, or taste co-occur with neurological symptoms?
- What are the long-term sequelae of these presentations, and how will they affect patients’ daily functioning, care needs, or quality of life?
- How frequently do neurological symptoms persist as part of “long COVID”? What are the different phenotypes and their natural history?
- To what extent do similar neurological and HEENT manifestations occur in children with COVID-19?
- Are there consistent sex differences (e.g. for anosmia), and if so why do they occur?
- Can any of these presentations be ameliorated by COVID-19-specific treatments?

In summary, a wide range of neurological and HEENT manifestations have already been reported with COVID-19, building on insights about such manifestations from prior coronavirus outbreaks. Whereas the overall proportion of infections leading to serious neurological disease will hopefully remain small, this number can still be staggering, considering the millions of people that have been infected worldwide. Even if a small fraction is left with enduring neurological sequelae, the associated health burden and costs might be large. Although we still have much to learn about the frequency, natural history, and underlying mechanisms of these manifestations, they should serve as further motivation for healthcare professionals to test for COVID-19, be on the lookout for atypical presentations, and appropriately protect themselves and their patients during routine evaluations.

## Supplementary material

For supplementary material accompanying this paper visit https://doi.org/10.1017/cjn.2021.180. click here to view supplementary material
