# Supplementary material for: Neurological and Head/Eyes/Ears/Nose/Throat Manifestations of COVID-19: A Systematic Review and Meta-Analysis
Source: Can J Neurol Sci. 2021 Jul 21:1–18. doi: 10.1017/cjn.2021.180 (PMC8460425; doi:10.1017/cjn.2021.180)
Supplement: Supplementary file 1 [file S0317167121001803sup001.docx]

**TABLE OF CONTENTS**

SEARCH STRATEGY…………...........…………………………………………………………………………….2

SUPPLEMENTARY TABLES……………………………………………………………………………………….3

SUPPLEMENTARY FIGURES……………………………………………………………………………………41

SUPPLEMENTARY REFERENCES……………………………………………………………………………..57

**SEARCH STRATEGY**

**PubMed/Medline:**

1 exp COVID-19/

2 exp Coronavirus/

3 exp SARS Virus/ or exp SARS-CoV-2/ or SARS.mp.

4 MERS.mp. or exp Middle East Respiratory Syndrome Coronavirus/

5 1 or 2 or 3 or 4

6 exp Neurology/

7 neurological.mp.

8 eye.mp. or exp Eye/

9 ocular.mp.

10 exp Eye Diseases/ or ophthalmological.mp.

11 exp Ear/ or ear.mp.

12 exp Nose/ or exp Otorhinolaryngologic Diseases/ or nose.mp.

13 6 or 7 or 8 or 9 or 10 or 11 or 12 or 13

14 5 and 13

**Embase/Embase Classic:**

1 exp coronavirus disease 2019/ and COVID.mp.

2 exp Coronavirinae/

3 SARS.mp. or exp severe acute respiratory syndrome/

4 MERS.mp.

5 1 or 2 or 3 or 4

6 exp neurology/

7 exp neurological complication/ or neurological.mp. or exp neurologic disease/

8 eye.mp. or exp eye/

9 ocular.mp.

10 ophthalmological.mp.

11 exp ophthalmology/

12 ear.mp. or exp ear/

13 exp ear nose throat disease/ or exp nose/ or nose.mp.

14 6 or 7 or 8 or 9 or 10 or 11 or 12 or 13

15 5 and 14

**Google Scholar** (since March 2020 for unindexed new papers):

(“coronavirus” OR “COVID” OR “SARS” OR “MERS”) AND (“neurology” OR “neurological” OR “eye” OR “ocular” OR “ophthalmological” OR “ear” OR “nose” OR “ENT”)

**SUPPLEMENTARY TABLES**

**Supplementary Table 1. Summary of key case series and reports on HEENT and neurological manifestations of SARS-CoV-1 and MERS**

| **Location** | **Outbreak** |  | **Total patients** | **Key finding or presentation** |
| --- | --- | --- | --- | --- |
| Taiwan^1^ | SARS |  | 1 | - Acute onset complete anosmia 3 weeks after onset of SARS symptoms - Normal MRI Brain - Anosmia persisted for over 2 years |
| **Central nervous system (CNS) manifestations** | | | | |
| Singapore^2^ | SARS |  | 206 | - 5 of 206 patients had large artery strokes - 3 of these 5 patients had been treated with intravenous immunoglobulin (IVIG) which can precipitate thromboembolism |
| Taiwan^3^ | SARS |  | 664 | 5 of 664 patients developed large artery strokes |
| Saudi Arabia^4^ | MERS |  | 70 | - Neuropsychiatric symptoms (including psychosis) in 25.7% - Seizures observed in 8.6% |
| **Peripheral nervous system (PNS) manifestations** | | | | |
| Taiwan^5^ | SARS |  | 1 | Polyradiculopathy with SARS-CoV-1 |
| Taiwan^6^ | SARS |  | 4 | - 3 patients had axonal polyneuropathy - One with myopathy - One with mixed electrophysiological findings |
| Hong Kong^7^ | SARS |  | 8 | - Eight patients underwent post-mortem skeletal muscle studies - Four were found to have focal myofiber necrosis with scant macrophage infiltration - Myofiber atrophy seen may have been explained by steroid use |
| Taiwan^3^ | SARS |  | 664 | - 3 of 664 patients had rhabdomyolysis - 3 patients had axonal polyneuropathy 3-4 weeks after onset of SARS, thought to be critical illness myopathy - 2 patients with myopathy, thought to be critical illness neuropathy |
| South Korea^8^ | MRS |  | 23 | - Four patients had neurological symptoms, with potential diagnoses including Bickerstaff’s encephalitis overlapping with Guillain-Barre syndrome (GBS), critical illness-associated weakness, or other toxic or infectious neuropathies - Symptoms generally delayed by 2-3 weeks |

**Supplementary Table 2. Key evidence on interesting HEENT manifestations of COVID-19 from cohort or case-control studies and large series (≥10 patients).** Studies are shown in descending order by sample size.

| **Location** | **Study Type** | **Number of Patients** | **Key findings** |
| --- | --- | --- | --- |
| **Anosmia or Ageusia** | | | |
| Iran^9^ | Cross-sectional, ecological study | 10,069 | - Participants completed an online checklist - 48.2% had dysosmia – sudden onset in 76.2% - 83.4% with anosmia also had dysgeusia - There was a significant correlation between the proportion of respondents reporting dysosmia in a given Iranian province and the reported number of COVID-19 cases in that province |
| Multi-national^10^ | Cross-sectional | 4,029 | - Questionnaire assessing self-reported quantity and quality of perception in smell, taste and chemesthesis before and during COVID-19 infection using a maximum possible change of ± 100 - Questionnaire revealed mean reduction of smell (-79.7), taste (-69.0), and chemesthetic (-37.3) function during COVID-19 infection - Perceived nasal obstruction did not account for smell loss |
| 18 European hospitals^11^ | Cross-sectional | 2,153 | - Consecutive ambulatory and hospitalized patients with positive RT-PCR, mild-to-moderate COVID-19 - Web-based olfactory and gustatory assessment questionnaire - 87% reported loss of smell, whereas 56% reported taste dysfunction. |
| Wuhan, China^12^ | Mixed prospective and retrospective cohort study | 1,733 | - 1,733 or 2,469 patients with COVID-19 discharged from a single hospital were followed - All patients were interviewed with a series of questionnaires for evaluation of symptoms and health-related quality of life, underwent physical examinations and a 6-min walking test, and received blood tests, with mean follow-up time after symptom onset of 186 days (~6 months) - Persistent smell disorder endorsed by 176 of 1,655 patients (6%), taste disorder by 120 (7%) |
| Greater Paris, France^13^ | Prospective observational study | 1,487 | - 57% had body aches/myalgia - 55% had headache - 28% had anosmia, 28% had ageusia - Females had increased prevalence of HEENT symptoms and headaches |
| Paris, France^14^ | Prospective multicentre cohort study | 55 and 1,824 | - Among 55 patients consulting primarily for loss of smell, 92.7% tested positive for COVID-19 - Anosmia in 80% (of 55 patients) was associated with a taste disorder - 72.9% of 55 patients partially recovered sense of smell within 15 days - In a population of 1,824 patients testing positive for COVID-19 the positive predictive value and the specificity of loss of smell was 78.5% - Positive predictive value and the specificity of loss of taste was 90.3% |
| France, Italy, Spain, Belgium, Switzerland^15^ | Cross-sectional | 1,420 | - COVID-19 positive patients completed questionnaire - 70.3% reported headache, 70.2% had loss of smell, 67.8% had nasal obstruction - 63.2% reported cough, 63.3% asthenia, 62.5% myalgia, 54.2% had gustatory dysfunction - Young patients more frequently had ear, nose and throat complaints - Loss of smell, headache, nasal obstruction and fatigue was more reported in females - Loss of smell was highly associated with mild-to-moderate COVID-19 and was not associated with nasal obstruction and rhinorrhea - Loss of smell persisted 7 days after the disease and resolved in 37.5% of patients |
| Spain^16^ | Cross-sectional | 909 | - Questionnaire to patients with SARS-CoV-2 infection and olfactory/gustatory alterations - 90.65% of patients reported both olfactory and gustatory involvement - Patient responses to questionnaire revealed 64.1% ageusia, 28.2% hypogeusia, 2.4% dysgeusia, 82.8% anosmia, 15.6% hyposmia, 0.9% dysosmia - 54.0% did not report concomitant nasal congestion or mucus |
| Montpellier, France^17^ | Prospective study | 809 (58 with COVID) | - Participants with no or mild symptoms evaluated with the Clinical Olfactory Dysfunction Assessment and standard olfactory testing prior to RT-PCR testing for SARS CoV2 - 35% of 58 with confirmed COVID-19 reported olfactory and/or gustatory dysfunction vs only 4% of 751 without COVID-19 |
| Europe^18^ | Prospective study | 751 | - Survey-based data collection and telemedicine follow-up - 621 (83%) reported a total loss of smell - 130 (17%) reported partial loss of smell - 277 (37%) patients still reported loss of smell, 107 (14%) reported partial recovery of smell, 367 (49%) reported complete recover of smell after mean follow up of 47 ± 7 days |
| Brazil^19^ | Cross-sectional | 725 | - Outpatients in Brazil that reported partial or total loss of smell completed online questionnaire - Total or partial sudden loss of the sense of smell demonstrated high positive predictive value for COVID-19 (88.8%) |
| Dresden, Germany^20^ | Cross-sectional | 500 | - 500 patients who presented with symptoms of a common cold to COVID-19 testing completed a standardized diagnostic questionnaire - 69/500 presented with olfactory loss - 22/69 tested positive for SARS-CoV-2 - 12 out of patients without olfactory loss tested positive for SARS-CoV-2 - Excluding patients with a blocked nose, “sudden smell loss” had high specificity (97%) and sensitivity of 65% with a positive predictive value of 63% and a negative predictive value of 97% for COVID-19 |
| 12 European hospitals^21^ | Cross-sectional | 417 | - Enrolled patients with mild-to-moderate COVID-19 who completed olfactory/gustatory questionnaires - 85.6% reported olfactory dysfunction - 88.0% reported gustatory dysfunction - 72.8% of patients recovered olfactory function within the first 8 days - 3.4% of patients recovered olfactory function after 15 days or longer |
| Washington State^22^ | Retrospective chart review | 404 | - 27(6.7%) of 404 consecutive patients with COVID-19 presented with impaired taste - 18(4.5%) presented with impaired smell |
| China, France, Germany^23^ | Multicenter case series | 394 | - Patients completed Questionnaire of Olfactory Disorders (QOD) and visual analog scale (VAS) - 161 (41%) had olfactory/gustatory disorders - 10% had only olfactory or gustatory symptoms - 19% had olfactory and/or gustatory complaints prior to other COVID-19 symptoms |
| Novara, Italy^24^ | Cross-sectional | 355 | - Patients answered a questionnaire at 14^th^ (or more) days after proven SARS-CoV-2 infection - Prevalence of both smell and taste disorders or one of the two was 70% - 8.7% of patients has smell and taste disorders as the first symptoms - 49.5% of cases fully recovered after 14 days, with median recovery time of 10 days |
| Stockholm, Sweden^25^ | Prospective cohort | 323 | - 2,149 healthcare professionals at a single centre were enrolled in the COMMUNITY (COVID-19 Biomarker and Immunity) study to investigate symptoms after mild COVID-19 - Questionnaires at baseline and follow-up were used to assess symptoms including anosmia/ageusia - Among 323 seropositive patients, 47 (14.6%) had anosmia and 25 (7.7%) had ageusia lasting at least two months, versus 0.6% each in the remaining seronegative patients |
| Spain^26^ | Cross-sectional | 230 | - Telephonic interview and questionnaire performed to healthcare workers tested positive for COVID-19 - 157 (68%) described olfactory alteration - 161 (70%) described gustatory alteration - Olfactory and gustatory malfunctions occurred before any other COVID-19 symptom - Duration of sensory deficits was average of 11 days, ranging from 2 to 45 |
| France^27^ | Cross-sectional | 229 | - COVID-19 patients interviewed by phone based on questionnaire - 140 patients reported sudden olfactory/gustatory disorders - 95.71% of patients reported to start an olfactory recovery at time of survey (administered 26 days of the mean time from anosmia onset) - Mean time from olfactory loss onset to recovery onset was 11.6 days - 51.43% of patients had complete olfactory recovery - Short time smell loss to olfactory recovery onset, absence of nasal obstruction and absence of sore/tingling feeling in nose were significantly related to the chance for complete olfactory recovery |
| Wuhan China^28^ | Retrospective case series | 217 | - 5.1% of hospitalized COVID-19 patients had dysosmia - 5.6% reported to have dysgeusia - Relied on records and patient complaints |
| Treviso, Italy^29^ | Case series, consecutive patients | 202 | - 64.4% of mildly symptomatic COVID-19 patients reported some dysosmia or dysgeusia - More often in women (72.4%) than men (55.7%) - Among these, 34.6% also had a blocked nose - Dysosmia/dysgeusia was the first symptom in 11.9% and simultaneous with others in 22.8% |
| Treviso, Italy^30^ | Prospective survey study | 202 | - Follow-up survey of patients above, 4-weeks from onset - 48.7% reported complete resolution of smell/taste impairment, 40.7% had improvement, 10.7% reported no change - Persistent loss of smell or taste was not associated with persistent SARS-CoV-2 infection |
| Italy^31^ | Cross-sectional | 180 | - A 15-item online questionnaire was administered to Italian general practitioners registered to a social media group - 46.7% of subjects presented sudden olfactory loss as an initial symptom - 19.2% of confirmed COVID-19 cases presented sudden olfactory loss as only symptom |
| Istanbul, Turkey^32^ | Cross-sectional | 172 | - Questionnaire given to patients with positive COVID-19 diagnosis - 51.2% of patients reported loss of sense of smell with mean recovery time of 8.02 ±6.41 days - 47.1% of patients reported loss of sense of taste mean recovery time of 8.20 ±7.07 days |
| China^33^ | Multicenter, prospective cohort study | 145 | - 145 patients with COVID-19 with dysosmia and 170 healthy subjects completed smell identification testing using Toyota-Takagi (T&T) olfactometry scores system - 16 (11%) of COVID-19 patients still had dysosmia 2-4 weeks after discharge; duration up to 95 days - Patients with COVID-19 with a smoking history were more likely to have long-term dysosmia |
| Missouri, USA^34^ | Retrospective observational analysis | 141 | - Symptomatology and duration extracted from collected clinical data and follow-up telephone consultations - 55% of COVID-19 patients reported anosmia and ageusia and ageusia - 9 patients reported only ageusia - 3 patients reported only anosmia - Median onset of anosmia in relation to onset of COVID-19 symptoms was 4 days and the median duration of anosmia was 8 days |
| Tel Aviv, Israel^35^ | Cross-sectional study | 140 | - Web-based questionnaire study of 140 ambulatory COVID-19 patients quarantined in a designated hotel - 58 men and 70 women: initial symptoms were cough (59.4%), weakness (47.7%), myalgia (46.9%), fever (42.2%), headache (40.6%), impaired sense of smell (38.3%), impaired sense of taste (32.8%), sore throat (26.6%), runny nose (26.6%), and nasal congestion (22.7%) - All symptoms were more frequent among women - 25.8% reported olfactory and taste dysfunction in the absence of other symptoms - 56% of patients reported xerostomia |
| Italy^36^ | Multicentre prospective study | 138 | - Olfactory and gustatory functions were prospectively evaluated for 60 days - 84.8% of patients had chemosensitive dysfunction within first 4 days - Significant increase in chemosensitive scores occurred in first 10 days for taste and between 10 and 20 days for smell - 7.2% of patients still had severe dysfunctions 60 days after symptom onset |
| Quebec, Canada^37^ | Age-matched case-control study | 134 | - 134 COVID-19 patients and 134 uninfected controls were given a standardized questionnaire - Out of identified 134 positive COVID-19 cases, 62.9% showed symptoms of anosmia, dysgeusia or both vs 8.2% of controls |
| Spain^38^ | Prospective | 131 | - 72 (55.0%) patients reported both taste dysfunction and anosmia - 52 (39.7%) patients reported neither taste dysfunction nor anosmia - 5 (3.8%) patients reported only anosmia - 2 (1.5%) patients reported only taste dysfunction - Taste disorders were found in 74 patients with 75.7% of those resulting in complete loss of taste, 10.9% metallic flavour, 8.1% bitter flavour, 4.0% salty flavour, and 1.3% loss of sweet flavour - Total recovery from anosmia occurred in 40.3% of cases and smell problems remained in 59.7% |
| [Italy](https://www.ncbi.nlm.nih.gov/pmc/articles/PMC7412289/)^39^ | Cross-sectional | 105 | - Patients completed survey - 78 (74.3%) patients reported altered sense of smell or taste - 88% of women had smell and taste disorders compared to 65% of women - 51.3% recovered smell and 60.3% recovered taste within 20 days from onset |
| Tehran, Iran^40^ | Prospective descriptive study | 92 | - 92 patients completed follow-up of mean 20.1 ± 7.42 days - 22 (23.91%) patients complained of olfactory loss - 6 (6.52%) patients had olfactory loss as first symptom - Olfactory loss was reported to be completely resolved in all but 1 patient - 39 (42.39%) patients had notable sinonasal symptoms - 15 (16.3%) patients had taste malfunction |
| Germany^41^ | Case-control study | 90 (45 with COVID-19) | - Subjects had to correctly identify 12 odours of Sniffin’ Sticks - 45 COVID-19 patients, 45 uninfected controls - COVID-19 patients on average could smell four fewer sticks than uninfected controls - 40% of COVID-19 patients diagnosed with anosmia and another 40% were diagnosed with hyposmia - No uninfected controls were diagnosed with anosmia and 27% were diagnosed with hyposmia - Not all patients returned to normal ability to smell 15 days after start of symptoms |
| Mons, Belgium^42^ | Case series | 86 | - Patient-reported outcome questionnaires followed by objective olfactory testing - 61.4% patients reported loss of smell - Objective olfactory testing identified 47.7% of patients to be anosmia, 14.0% to be hyposmia and 38.3% to be normosmic |
| Turkey^43^ | Prospective study | 81 | - 81 patients with COVID-19, 40 uninfected controls - Olfactory and gustatory testing completed - 61.7% COVID-19 patients had complaints related to olfaction (vs none in control group) - 27.2% of COVID-19 patients had loss of taste (vs none in control group) |
| Madrid, Spain^44^ | Case-control study | 79 | - 79 COVID-19 cases and 40 uninfected controls were examined using a self-reported questionnaire - Smell/taste disorders (new-onset) in 39.2% of cases versus 12.5% of controls - 70.9% of COVID-19 patients with dysosmia/dysgeusia described acute onset of the complaint |
| [Mons, Belgium](https://journals.sagepub.com/doi/10.1177/0145561320929169?url_ver=Z39.88-2003&rfr_id=ori:rid:crossref.org&rfr_dat=cr_pub%20%200www.ncbi.nlm.nih.gov#articleCitationDownloadContainer)^45^ | Prospective study | 78 | - Divided 46 patients that performed psychophysical olfactory evaluation into 2 groups based on olfactory dysfunction duration: group 1 for duration ≤ 12 days (group 1) and group 1 for > 12 days (group 2) - 52% of patients reported anosmia - 24% of patients reported hyposmia - 24% of patients were normosmic |
| Cologne, Germany^46^ | Retrospective study | 72 | - Patients were interviewed using a standardized 2-section questionnaire - 74% of COVID-19 patients had reduced olfaction - 69% of COVID-19 patients had reduced sense of taste - 68% of patients reported both reduced olfaction and sense of taste - 1% had only reduced sense of taste, 4% had only reduced olfaction - 54% of patients had nasal congestion |
| [Trevenans, France](https://pubmed.ncbi.nlm.nih.gov/32771635/)^47^ | Retrospective study | 70 | - 53% of patients had anosmia - 48% of patients had dysgeusia - Mean duration of anosmia was 7.4 (±5, [1-21]) days - 51% of patients recovered from anosmia before 28 days of evolution |
| Milan, Italy^48^ | Cross-sectional | 59 | - Among 59 interview-able patients admitted with COVID-19, 33.9% reported dysosmia/dysgeusia - 20.3% reported having these symptoms before hospitalization |
| Athens, Greece^49^ | Case-Control study | 22 | - 22 COVID-19 patients and 22 age/sex-matched controls completed a brief smell identification test and evaluated nasal congestion - Prevalence of normosmia was lower in cases (23%) than controls (64%) - Gustatory dysfunction was reported in 23% of cases - Among 16 cases with hyposomia and anosmia, nasal congestion was identified in 3 patients |
| [Hong Kong](https://www.ncbi.nlm.nih.gov/pmc/articles/PMC7284010/)^50^ | Case-Control study | 18 | - Olfactory function assessed through questionnaires and butanol threshold test (BTT) - 18 COVID-19 patients and 18 controls - 12 (67%) COVID-19 patients reported olfactory symptoms, olfactory dysfunction confirmed in 6 - Sinusitis and olfactory clef obstruction were absent in most patients - Olfactory dysfunction persisted in 2 patients despite clinical recovery |
| Germany, USA, Venezuela, Bolivia^51^ | Case series | 10 | - Seven patients presented overlapping anosmia/ageusia, while one patient presented only anosmia and another only ageusia - Postviral olfactory loss was more common in women with a female-to-male ratio of 2:1 |
| **Dysphagia or Dysphonia** | | | |
| 19 European Hospitals^52^ | Prospective cohort | 702 | - Clinical and epidemiological data of 702 patients with mild-to-moderate COVID-19 were collected - Dysphonia and otolaryngological symptoms were self-assessed through a 4-point - Outcomes were compared between dysphonic and nondysphonic patients - 188 (26.8%) patients were dysphonic, more often females and smokers. - There were significant associations between the severity of dysphonia, dysphagia, and cough. |
| **Conjunctivitis** | | | |
| China (30 provinces)^53^ | Retrospective cohort | 1,099 (552 hospitals) | - “Conjunctival congestion” reported in 9 (0.8%) hospitalized patients - Initial but vague signal of ocular manifestations of COVID-19 |
| New Delhi, India^54^ | Cross-sectional | 127 | - Of 12 (9.45%) patients who had ocular complaints, 11 (8.66%) had ocular manifestation after admission - Among 11 patients, 8 (6.29%) had conjunctival congestion - 3 patients had developed conjunctival congestion before manifestation of definite COVID-19 symptoms - 5 patients out of the 8 had no other associated ocular symptoms other than congestion - 6 patients out of the 8 had symptoms of upper respiratory tract infection |
| Wuhan, China^55^ | Cross-sectional | 121 | - 8 (6.6%) COVID-19 patients presented ocular symptoms; only 1 of them showed positive results for SARS-CoV-2 in the conjunctiva and also categorized as a severe/critical case - Ocular symptoms and abbreviated findings included: itching (5 [62.5%]), redness (3 [37.5%]), tearing (3 [37.5%]), discharge (2 [25%]), and foreign body sensation (2 [25%]) |
| Wuhan, China^56^ | Cross-sectional | 102 | - Patients with clinical diagnosis of COVID-19: 72 with laboratory confirmed RT-PCR assay - Only two patients (2.78%) with conjunctivitis were identified - SARS-CoV-2 RNA fragments found in ocular discharges in only one patient |
| Hubei, China^57^ | Case series | 38 | - 12 (31.6%) hospitalized patients had various combinations of conjunctivitis features including hyperemia, chemosis, epiphora, and secretions - One patient had epiphora as the first symptom - Patients with ocular symptoms had higher white blood cell and neutrophil counts and high levels of C-reactive protein (CRP), procalcitonin, and lactate dehydrogenase |
| Zhejiang, China^58^ | Case series | 30 | - Only one (3.3%) hospitalized patient had conjunctivitis; no other ocular symptoms |
| **General Ocular Manifestations** | | | |
| Wuhan, China^59^ | Cross -sectional study | 535 | - 27/535 (5%) presented with conjunctival congestion and 4 patients had conjunctival congestion as the initial symptom - Other ocular symptoms, including increased conjunctival secretion, ocular pain, photophobia, dry eye and tearing, were also found in patients with conjunctival congestion - Hand–eye contact was independently correlated with conjunctival congestion in COVID‐19 patients - Some COVID‐19 patients had chronic eye diseases, including conjunctivitis (33, 6.2%), xerophthalmia (24, 4.5%) and keratitis (14, 2.6%) |
| Diyarbakır, Turkey^60^ | Cross‐sectional | 359 | - 294/359 patients with SARS‐CoV‐2 were treated in the inpatient clinic and 65/359 (18.1%) patients were treated in the intensive care unit. - 16 (4.5%) of the patients presented various ocular diseases and the rate of ocular disease was 12/294 (4.1%) in patients followed up in the inpatient clinic and 4/65 (6.2%) in intensive care patients. |
| Nashville, Tennessee^61^ | Retrospective patient survey | 144 | - Out of all completed survey participants,144 (32.0%) were COVID-19 positive, and 306 (68.0%) were COVID-19 negative - 144/450 (32.0%) surveys were completed 1-4 weeks after positive results for COVID-19 - 68/144 (47%) of COVID-19–positive patients reported at least 1 overlapping eye-related symptom - Most commonly reported ocular symptoms were eye pain (19.4%), photophobia (13.9%), flashes or floaters (11.8%), blurry vision (11.1%), and red eyes (10.4%) - 20.6% (14/68) noted ocular symptoms before systemic symptoms - 26.5% (18/68) of respondents still experiencing persistent eye symptoms despite recovery from systemic illness |
| Northeast of Iran^62^ | Cross-sectional study | 142 | - 142 COVID-19 patients - 44 (31%) patients found to have conjunctival hyperemia , most prevalent ocular finding in all patients - 22 (15.5%) patients had chemosis, most common ocular manifestation in ICU admitted patients - Consecutive slit examination showed 11 (7.7%) cataract, and 9 (6.3%) diabetic retinopathy. - None of the patients reported ocular symptoms prior to systemic involvement. |
| Hong Kong, China^63^ | Prospective study | 56 | - 56 patients with SARS‐CoV‐2 recruited to evaluate ocular symptoms - 27% reported ocular symptoms in the course of COVID‐19, including sore eyes, itching, foreign body sensation, tearing, redness, dry eyes, eye secretions and floaters - 11% of those with ocular symptoms presented with ocular symptoms before onset of fever or respiratory symptoms, ranging from 1-7 days - Conjunctival swab sample from left eye of one patient showed positive SARS-CoV-2 RNA detection |
| Hubei, China^57^ | Case series | 38 | - 38 patients with COVID-19: 12 had ocular manifestations like epiphora, conjunctival congestion, or chemosis, and these commonly occurred in patients with more severe systemic manifestations - RT-PCR was positive for SARS-CoV-2 in 28 nasopharyngeal swabs and 2 conjunctival swabs, and more significant changes in blood test values appeared in patients with ocular abnormalities |
| São Paulo, Brazil^64^ | Case series | 12 | - 12 adults (6 men and 6 women, aged 25–69), examined 11–33 days after COVID-19 symptom onset. - All patients showed hyper-reflective lesions at the level of ganglion cell and inner plexiform layers more prominently at the papillomacular bundle in both eyes - First report of retinal findings possibly associated with COVID-19 infection in humans |

**Supplementary Table 3 – Relevant case reports and small series (<10 patients) on interesting HEENT manifestations of COVID-19.** Studies are shown in descending order by sample size.

| **Location** | **Study Type** | **Number of Patients** | **Key findings** |
| --- | --- | --- | --- |
| **Anosmia or Ageusia** | | | |
| Tehran, Iran^65^ | Case series | 8 | - Eight patients ranging from 22 to 44-years-old reported anosmia and sudden olfactory loss 2-4 days after onset of fever and other mild respiratory symptoms - 5 out of 8 patients were positive for COVID-19, the 3 others were not tested for COVID-19 |
| London, United Kingdom^66^ | Case series | 4 | - Four patients ranging from 28 to 37-years-old presented with severe loss of smell (two additional loss of taste) and no or mild nasal congestion, with COVID-19 antibody testing positive several weeks later in two patients but negative in two others |
| Italy^67^ | Case report | 3 | - A 25-year-old female presented with a mild dry cough lasting for 1 day, followed by persistent severe anosmia and dysgeusia - 3D and 2D fluid-attenuated inversion recovery images showed cortical hyperintensity in the right gyrus rectus and subtle hyperintensity in olfactory bulbs, tested positive for COVID-19 - Recovery of anosmia occurred after 28 days - No brain abnormalities were seen in 2 other patients with COVID-19 presenting anosmia |
| London, UK^68^ | Case series | 3 | - Three patients ranging from 37 to 53-years-old tested positive for COVID-19 and had initial onset of loss of smell 1 week prior, 5 days prior, and 2 days after symptoms |
| Paris, France^69^ | Case series | 3 | - Patients (23 to 57-years-old) with COVID-19 and loss of smell/taste, nasal obstruction, headache in addition to typical upper respiratory infection symptoms - All had MRI performed that showed cervical lymphadenopathy |
| Zurich, Switzerland^70^ | Case series | 2 | - Two cases of olfactory neuropathy with axonal damage, prominent leukocytic infiltrates in the lamina propria, and focal mucosal atrophy in the olfactory epithelium identified at autopsy - Both deceased patients had reported anosmia with COVID-19 |
| Korea^71^ | Case report | 1 | - 53-year-old female patient with ankylosing spondylitis treated with entanercept 1 month after being diagnosed with COVID-19 complained about hyposmia and hypogeusia |
| Australia^72^ | Case report | 1 | - 61-year-old woman presented with lethargy, headaches, hearing impairment, anosmia, ageusia and worsening dyspnoea three days prior to presentation - Relevant history included cruise ship travel with known SARS-CoV-2 cases - Hearing impairment, anosmia and ageusia completely resolved on day 4 |
| London, UK^73^ | Case report | 1 | - 48-year-old neurosurgeon presented with sudden onset of complete anosmia, with no other symptoms, and two days later confirmed SARS-CoV-2 infection |
| Marseille, France^74^ | Case report | 1 | - 27-year old man experienced complete anosmia and dysgeusia four days after COVID-19 diagnosis - After seven days, bilateral olfactory bulb edema showed on 1.5T MRI - Anosmia and dysgeusia resolved on day 14 and normalization of olfactory bulb signal and volumes occurred on day 24 |
| Paris, France^75^ | Case report | 1 | - A woman in her 40s presented with an acute loss of olfactory function without nasal obstruction a few days after experiencing a dry cough - Patient showed bilateral inflammatory obstruction of the olfactory clefts - Tested positive for COVID-19 |
| Philadelphia, USA^76^ | Case report | 1 | - 59-year-old woman with initial dysgeusia and anosmia followed by respiratory deterioration - Anosmia recovered by dysgeusia persisted at discharge 10 days later |
| Kuwait^77^ | Case report | 1 | - 25-year-old woman with COVID-19 presented with fever, body pain, dry cough, anosmia, ageusia - As anosmia and ageusia started to improve the following month, she experienced cacosmia and cacogeusia with stimulation of smell or taste, persisting for 3 months despite different therapies - MRI Brain was normal but task-based fMRI showed absent activation in the orbitofrontal cortex while the right uncus/piriform cortex showed strong BOLD signal |
| **Conjunctivitis (and other ocular inflammation)** | | | |
| Italy^78^ | Case series | 5 | - Four middle-aged males and one female presented with conjunctivitis with recent travel history abroad - Tested positive for COVID-19 with conjunctivitis as their presenting sign and symptom and remained the sole manifestation of COVID-19 |
| Wuhan, China^79^ | Case series | 2 | - Two cases of a 29-year-old male and 51-year-old female COVID-19 patients presenting with ocular manifestation of conjunctival congestion - SARS-CoV-2 was detected in both. Conjunctival samples |
| Chaco, Argentina^80^ | Case report | 1 | - 27-year-old male presented foreign body sensation and red left eye and no systemic symptoms - 3-Hours later presented severe headache and fever - 12-hours later developed cough and severe dyspnea impairing speech - Nasopharyngeal swabs resulted positive for SARS-CoV-2 |
| Clermont-Ferrand, France^81^ | Case report | 1 | - 63-year-old male COVID-19 patient admitted to intensive care unit (ICU), seven days after beginning of influenza-like symptoms - Day 19 patient showed pseudomembranous conjunctivitis - Conjunctival swabs and testing did not identify SARS-CoV-2 |
| Edmonton,  [Canada](https://www.ncbi.nlm.nih.gov/pmc/articles/PMC7124283/)^82^ | Case report | 1 | - 29-year-old female with travel history to the Philippines presented with 1-day history of right eye conjunctivitis, photophobia, and clear watery discharge from right eye - 5-days after initial visit, positively tested for COVID-19 - 3-days after initial visit, retrospective testing of eye swab originally submitted for gonorrhea/chlamydia was found to be weakly positive for SARS-CoV-2 |
| Istanbul, Turkey^83^ | Case report | 1 | - 32-year-old male nurse presented 1-day history of right eye conjunctivitis, photophobia, and clear watery discharge from right eye - 3-days after initial visit, nasopharyngeal RT-PCR test was applied to him and a positive result was reported for SARS-CoV-2. The patient had negative RT-PCR results for eye samples. Routine adenovirus tests also yielded a negative result. |
| Malaysia^84^ | Case report | 1 | - 54-year-old female presented with conjunctivitis lasting 4 days, starting on day 11 after travel abroad, with no other symptoms, tested positive for COVID-19 |
| Odisha, India^85^ | Case report | 1 | - 65-year-old known diabetic, hypertensive, and asthmatic patient COVID-19 positive by day 7 from the time of ventilation presented redness and severe injection of the conjunctiva in the right eye - Conjunctival swabs for bacteria and fungus were negative, patient diagnosed with acute follicular conjunctivitis |
| Rome, Italy^86^ | Case report | 1 | - 72-year-old male Italian asymptomatic COVID-19 patient, with type 2 diabetes mellitus, treated with metformin, and with high blood pressure treated with specific drugs - Only conjunctivitis had been diagnosed |
| Shenzhen, China^87^ | Case report | 1 | - 30-year-old male COVID-19 patient presented bilateral acute conjunctivitis on 13 days after illness onset. Transcription PCR (RT-PCR) detected SARS-CoV-2 virus in conjunctival swabs - The conjunctival swab specimens remained positive for SARS-CoV-2 on 14 and 17 days after onset - On day 19, RT-PCR result was negative for SARS-CoV-2 |
| Tabriz, Iran^88^ | Case report | 1 | - 65-years-old diabetic male presented with burning eye and discharge for 2-days - 2- Days later, he confirmed the diagnosis of COVID-19 - RT-PCR testing of the conjunctival secretion was positive for COVID-19 at two separate occasions |
| Zhejiang China^89^ | Case report | 1 | - 53-year-old male confirmed with COVID-19 developed symptoms of viral conjunctivitis in the left eye 10 days after the onset of COVID-19 - Symptoms relieved 6 days after treatment - Patient subsequently diagnosed with viral keratoconjunctivitis in both eyes 5 days after the symptoms in the left eye were relieved - SARS-CoV-2 could not be detected in conjunctival secretions |
| Jerusalem, Israel^90^ | Case report | 1 | - 54‐year‐old female admitted with multisystem inflammatory syndrome secondary to COVID‐19 infection - After two weeks, complained of blurry vision – found to have bilateral anterior uveitis |
| Barcelona, Spain^91^ | Case report | 1 | - 31‐year‐old female positive for COVID‐19 with no relevant history of ocular complications, presented 7-days after COVID-19 onset, red eye, foreign‐body sensation, epiphora, photophobia - Diagnosed with nodular episcleritis: resolved on sixth day with treatment of artificial tears on demand and fluorometholone five times a day for 3 days, tapered during the following weeks |
| **Retinal Artery Occlusion (and other ocular vascular events)** | | | |
| Staten Island, USA^92^ | Case report | 1 | - 60-year-old male with a history of hypertension, dyslipidemia, stable coronary artery disease and chronic obstructive pulmonary disease presented by hospitalization day 20 sudden onset of painless loss of vision in the right eye - Found to have Central Retinal Artery Occlusion (CRAO) |
| Los Angeles, USA^93^ | Case Report | 1 | - 48-year-old male was hospitalized with severe form of COVID‐19 - 3 weeks later pending hospital discharge, the anticoagulation was switched to oral apixaban - 37 hours after the last dose of enoxaparin and 24 hours after apixaban was started, the patient developed sudden-onset painless right eye vision loss. - Immediate ophthalmological examination showed visual acuity of no light perception in the right eye and 20/20 in the left eye. - Treatment was changed to enoxaparin and no thrombotic events were encountered to date |
| Providence, USA^94^ | Case report | 1 | - Female in her 50s, with a history of hypertension, hyperlipidemia and COVID-19 positive presented with dysosmia, dysgeusia, along with monocular peripheral vision loss - Patient vision spontaneously improved during her hospitalization, and she was discharged home on aspirin and atorvastatin |
| Madrid, Spain^95^ | Case report | 1 | - 40-year old male with COVID-19 and papillophlebitis causing dilated, tortuous retinal vessels, disc edema, and retinal hemorrhages – treated with dexamethasone implant with resolution |
| **Other Ocular Manifestations** | | | |
| London, UK^96^ | Case series | 2 | - 37-year-old female and 32-year-old male with COVID-19 presenting with new paracentral scotoma - Diagnosed as paracentral acute middle maculopathy and acute macular neuroretinopathy |

**Supplementary Table 4. Key evidence of general neurological manifestations of COVID-19 from cohort studies and large case series (>10 patients).** Studies are shown in descending order by sample size.

| **Location** | **Study Type** | **Number of Patients** | **Key findings** |
| --- | --- | --- | --- |
| Multi-national (but primarily USA)^97^ | Retrospective cohort study | 236,379 | - Analyzed COVID-19 adult patients (index event 20-Jan-2020 and alive on 13-Dec-2020) in TriNetX, a global health collaborative clinical research platform claiming to collect real-time electronic medical records data - Also had matched control cohorts of patients diagnosed with influenza and other respiratory tract infections (n=236,038) - Estimated 6-month incidence of 14 neurological and psychiatric outcomes: 33.62% for any symptom, 0.56% for intracranial hemorrhage, 2.10% (1·97–2·23) for ischemic stroke, 0.11% (0.08–0.14) for parkinsonism, 0.67% (0.59–0.75) for dementia, 17.39% (17.04–17.74) for anxiety disorder, and 1.40% (1.30–1.51) for psychotic disorder, among others. - In the subgroup with intensive care admission, estimated incidences were 2.66% (2.24–3.16) for intracranial hemorrhage, 6.92% (6.17–7.76) for ischemic stroke, 0.26% (0.15–0·45) for parkinsonism, 1.74% (1.31–2.30) for dementia, 19.15% (17.90–20.48) for anxiety disorder, and 2.77% (2.31–3.33) for psychotic disorder. - Most diagnostic categories were more common in patients who had COVID-19 than in those who had influenza (hazard ratio [HR] 1.44, 95% CI 1.40–1.47) and those who had other respiratory tract infections (1.16, 1.14–1.17) - HRs were higher in patients who had more severe COVID-19 |
| Multi-national (but primarily USA)^98^ | Retrospective analysis | 40,469 | - Analyzed COVID-19 adult patients in TriNetX - 9,086 (22.5%) patients had neuropsychiatric manifestations - Most common neurologic manifestations included headache (3.7%), sleep disorders (3.4%), and encephalopathy (2.3%) - Stroke and TIA in 1.0%, seizures in 0.6% - Psychiatric manifestations included anxiety and other related disorders (4.6%), mood disorders (3.8%); 0.2% had suicidal ideation |
| Bronx, USA^99^ | Retrospective cohort study | 4,711 | - Examined records of all COVID-19 patients admitted to their hospital system - 581 hospitalized patients with confirmed SARS-CoV-2 infection and neurologic involvement warranting neuroimaging (12% of total), were compared to 1,743 age-/severity-matched COVID-19 non-neurologic patients - Four patterns of neurologic manifestations were identified: acute stroke, new or recrudescent seizures, altered mentation with normal imaging, and “neuro-COVID-19” complex: normal orientation and arousal with signs and symptoms including headache, anosmia, ageusia, chemesthesis, vertigo, presyncope, paresthesias, cranial nerve abnormalities, ataxia, dysautonomia, and/or skeletal muscle injury - Patients with altered mentation or stroke had a higher risk of in-hospital mortality |
| New York City, USA^100^ | Prospective multi-centre cohort study | 4,491 | - Hospitalized patients with COVID-19 followed until discharge - 606 (13.5%) developed a new neurologic disorder in a median of 2 days from onset - Most common diagnoses were toxic/metabolic encephalopathy (6.8%), seizure (1.6%), stroke (1.9%), and hypoxic/ischemic injury (1.4%) - 18/18 CSF specimens were reverse transcriptase PCR negative for SARS-CoV-2 - Patients with neurologic disorders were more often older, male, white, hypertensive, diabetic, intubated, and had higher sequential organ failure assessment (SOFA) scores - Patients with neurologic disorders had increased in-hospital mortality and decreased likelihood of discharge home |
| Wuhan, China^12^ | Mixed prospective and retrospective cohort study | 1,733 | - 1,733 or 2,469 patients with COVID-19 discharged from a single hospital were followed - All patients were interviewed with a series of questionnaires for evaluation of symptoms and health-related quality of life, underwent physical examinations and a 6-min walking test, and received blood tests, with mean follow-up time after symptom onset of 186 days (~6 months) - Fatigue or muscle weakness (63%) and sleep difficulties (26%) were the most common persistent symptoms - Anxiety or depression was reported among 23%, headache in 2% |
| Sao Paulo, Brazil^101^ | Retrospective chart review | 1,208 | - Identified inpatient neurology consultations over two months among COVID-19 patients - Consultations were requested for 89/1,208 (7.4%) admissions - Main diagnoses included: encephalopathy (44.4%), stroke (16.7%), previous neurological diseases (9.0%), seizures (9.0%), neuromuscular disorders (5.6%), other acute brain lesions (3.4%), and other mild nonspecific symptoms (11.2%) |
| China^102^ | Retrospective multicenter cohort study | 917 | - Frequency of new onset critical neurologic events was 3.5% (32/917) overall and 9.4% (30/319) among those with severe/critical COVID-19 - Critical neurologic events highly associated with older age (above 60-years-old) and previous health history of neurologic conditions - Less than 1% (7/917) had non-critical neurologic events such as muscle cramp, headache, occipital neuralgia, tic and tremor |
| Castilla-La Mancha, Spain^103^ | Cross-sectional | 841 | - Systematically reviewed patients diagnosed with COVID-19 - 57.4% developed neurologic symptom - 14.1% had headache, 6.1% had dizziness, presented mostly in early stages of infection - 4.9% anosmia, 6.2% dysgeusia, presented mostly in early stages and frequent in less severe cases - 19.6% had disorders of consciousness, presented mostly in older patients in severe and advanced stages of infection - 3.1% had myopathy, 2,5% had dysautonomia, 1.7% had cerebrovascular diseases, 0.7% had seizures, 0.7% had movement disorders, reported but less frequent - Neurologic complications were cause of death in 4.1% of deceased study participants |
| China^104^ | Retrospective cohort study | 571 | - 9.6% had headaches - 16.6% had fatigue, muscle aches |
| Washington State, USA^22^ | Cross-sectional | 404 | - 295 (73%) of 404 COVID-19 patients reported neurological symptom - 208 (51.5%) of 404 COVID-19 patients presented with CNS symptoms; 21.3% had altered mental status, 20.3% had headache, 7.7% had dizziness - Of the 86 patients with altered mental status, 57.0% had pre-existing dementia - 163 (40.3%) of 404 COVID-19 patients presented with PNS symptoms; 32.4% had myalgia, 6.7% had gustatory malfunction, 4.5% had olfaction malfunction |
| Zengdu District, China^105^ | Cross-sectional | 276 | - Collected clinical data on COVID-19 inpatients at a single centre - 8.7% had headache - 0.7% had conjunctival congestion - 2.9% had nasal congestion - 9.4% had myalgia or arthralgia |
| Turkey^106^ | Prospective study | 239 | - 34.7% of COVID-19 patients had neurological symptoms - 27.6% had headache - 9 (3.8%) patients had cerebrovascular disease; 3 transient ischemic attacks, 4 ischemic cerebrovascular disease, 2 hemorrhagic cerebrovascular disease - D-dimer blood levels significantly higher in patients with one neurological symptom than those without neurological symptom - IL-6 was found to be significantly higher in patients with headache than those without |
| Wuhan, China^28^ | Case series | 214 | - Case series of 214 patients with COVID-19 - 36.4% had neurological symptoms - 45.5% of patients with severe COVID-19 had neurological symptoms, including cerebrovascular events, impaired consciousness, and muscle injury |
| Rome, Italy^107^ | Retrospective study | 213 | - 30% of COVID-19 patients presented neurological symptoms - COVID-19 patients had a higher frequency of headache, hyposmia and encephalopathy than 218 uninfected patients (controls, 21%) - Muscular involvement more frequent in COVID-19 patients than uninfected patients |
| Wuhan, China^108^ | Singe-center case series | 138 | - 34.8% of patients had myalgia - Less common symptoms were headache (6.5%), dizziness (9.4%), abdominal pain, diarrhea, nausea, and vomiting |
| Wuhan, China^109^ | Retrospective study | 274 | - 113 deceased and 161 recovered COVID-19 were analyzed - Disorders of consciousness more common in deceased than recovered patients - 10% of deceased COVID-19 patients reported headache and 12% of recovered COVID-19 reported headaches |
| Bologna, Italy^110^ | Prospective study | 108 | - Questionnaire by weekly telephone interviews - Neurological symptoms reported in early phase of COVID-19, median onset 1 to 3 days - 43% had headache, 10% dizziness, 37% dysosmia, 61% dysgeusia, 34% myalgia - Patients with olfactory disorders less frequently needed oxygen therapy |
| Italy^111^ | Retrospective, multicentre study | 108 | - 108 COVID-19 patients with acute neurological symptoms who received neuroimaging - 59% had altered mental status, 31% had ischemic stroke - 66% had no acute findings on brain CT, out of which 35% brain MRI demonstrated acute abnormalities |
| Rome, Italy^112^ | Cross-sectional study | 103 | - Single-centre study of patients hospitalized with COVID-19 who were interviewed about subjective neurological symptoms (13 items) - 94 patients (91.3%) reported at least one symptom, most frequently sleep, dysgeusia, headache, hyposmia, and depression - Women more frequently complained hyposmia, dysgeusia, dizziness, numbeness/paresthesias, daytime sleepiness, and muscle ache - Higher white blood cells and lower C-reactive protein levels correlated with the occurrence of hyposmia, dysgeusia, headache, daytime sleepiness, and depression |
| Vizcaya, Spain^113^ | Prospective cohort study | 100 | - 100 consecutive, unselected COVID-19 patients evaluated systematically - 88% of patients had one neurological manifestation associated with COVID-19 - 44% had anosmia-dysgeusia, 44% headache; associated with younger patients in less severe cases - 43% had myalgia, 36% had dizziness - 8% had encephalopathy, 7% had syncope, 2% had seizures, 2% had ischemic stroke |
| Wuhan, China^114^ | Retrospective, single-centre study | 99 | - 9% of COVID-19 patients had confusion - 8% had headache |
| Boston, USA^115^ | Prospective cohort study | 74 | - Patients hospitalized with COVID-19 who had an inpatient neurologic or neurocritical care consultation identified by electronic medical records at a large safety-net hospital - Most were African-American or Black (51%) - Most common neurologic presenting symptoms were altered mental status (53%), fatigue (24%), and headache (18%) - 20% of patients developed ischemic stroke. Survivors had median modified Rankin Scale score of 4 (moderately severe disability) by discharge. |
| Beijing, China^116^ | Retrospective single-centre cohort | 86 (all critically ill) | - 65% of COVID-19 patients presented with at least one neurological symptom - 23.3% presented with CNS symptoms including delirium, cerebrovascular disease, hypoxic-ischemic brain injury - 7% presented with neuromuscular involvement - 7/86 COVID-19 patients exhibited new stroke, and 6/7 were ischemic; significantly higher prevalence of antiphospholipid antibodies observed in patients with ischemic stroke than those without |
| France^117^ | Retrospective multicentre cohort | 64 | - 64 COVID-19 patients with neurologic manifestations who underwent a brain MRI - 56% of brain MRIs were considered abnormal - 27% of brain MRIs revealed ischemic strokes, 17% revealed leptomeningeal enhancement, 13% revealed encephalitis - 53% of patients reported confusion, 39% impaired consciousness, 16% headache |
| Italy^118^ | Retrospective, single-center cohort study | 56 | - 56 positive COVID-19 patients, 117 uninfected patients (controls) admitted for neurological diagnoses (or to a neuro-COVID unit) - Incident delirium was significantly higher in COVID-19 patients (26.8% vs 7.7%) - Stroke patients with COVID-19 and without COVID-19 had similar characteristics, but COVID-19 had higher modified Rankin Scale scores at discharge |
| Beijing, China^119^ | Retrospective study | 55 | - 18.2% of COVID-19 patients had muscle ache, 10.9% had dizziness - 10.9% had headache |
| Chicago, USA^120^ | Retrospective study | 50 | - 60% of COVID-19 patients had altered mental status - 40% had cerebrovascular events; 20% stroke, 8% intracerebral hemorrhage, 8% non-aneurysmal subarachnoid hemorrhage, and 4% transient ischemic attack - 26% of patients had new onset seizures or breakthrough seizures - 24% had headache, 24% had cognitive abnormalities - 14% had hypoxic ischemic brain injury - 4% had posterior reversible encephalopathy syndrome - 12% had dysautonomia, 12% had muscle injury with elevated CK levels, - 10% had dysgeusia, 6% had hyposmia - 6% had isolated unilateral peripheral facial palsy |
| China^121^ | Cross-sectional study | 48 | - 48 COVID-19 patients, 1 of which was mild, 38 moderate, and 9 severe - 10.4% had headache, and another 10.4% had dizziness |
| Wisconsin and Utah, USA^122^ | Prospective cohort study | 47 | - 198 household contacts exposed to SARS-CoV-2 prospectively followed with two or more RT-PCR (nasopharyngeal) and symptom diaries in a 14-day period - 47 tested positive and most common symptoms on first day were upper respiratory (68%) followed by neurological (64% - headache, anosmia, dysgeusia) |
| Wuhan, China^123^ | Retrospective study | 41 | - 8% (3/38) had headache - 44% had myalgia or fatigue |
| Rome, Italy^124^ | Cross-sectional study | 22 | - Structured interview regarding subjective neurological symptoms and dyspnea in patients with non-severe COVID-19 (seen in 31.8%) - Prevalence of subjective neurological symptoms was 81.8% - mostly sleepiness (50%), ageusia (27.3%), headache (22.7%), anosmia (13.6%) - Neurological symptoms, dyspnea descriptors, respiratory rate, arterial blood gas markers were similar between groups |

**Supplementary Table 5. Key evidence of CNS manifestations of COVID-19 from cohort studies and large case series (>10 patients).** Studies are listed in descending order of sample size.

| **Location** | **Study Type** | **Number of patients** | **Key findings** |
| --- | --- | --- | --- |
| **Stroke and other cerebrovascular manifestations** | | | |
| Multi-national (but primarily USA)^97^ | Retrospective cohort study | 236,379 | - Analyzed COVID-19 adult patients (index event 20-Jan-2020 and alive on 13-Dec-2020) in TriNetX, a global health collaborative clinical research platform of electronic records - Also had matched control cohorts of patients diagnosed with influenza and other respiratory tract infections (n=236,038) - Estimated 6-month incidence of 14 neurological and psychiatric outcomes, including stroke: 0.56% (95%CI 0.50-0.63) for intracranial hemorrhage, 2.10% (1·97–2·23) for ischemic stroke - In the subgroup with intensive care admission, estimated incidences were 2.66% (2.24–3.16) for intracranial hemorrhage, 6.92% (6.17–7.76) for ischemic stroke - Ischemic stroke more common in patients who had COVID-19 than in those who had influenza (hazard ratio [HR] 1.62, 95% CI 1.43–1.83) and those who had other respiratory tract infections (1.45, 1.36–1.55), as was intracranial hemorrhage (vs influenza: 2.44, 1.89-3.16; vs any other respiratory infection: 1.26, 1.11-1.43) - HRs were higher in patients who had more severe COVID-19 |
| Multi-national, 11 countries^125^ | Mixed prospective/ retrospective cohort study | 26,175 | - 156(0.9%) hospitalized COVID-19 patients had a stroke - 79% were ischemic strokes - Model using 17,799 patients: overall stroke risk estimated to be 0.5% among all centres; mechanical ventilation and presence of ischemic heart disease were predictive of stroke |
| Multi-national (USA, UK, Spain, Italy)^126^ | Retrospective cross-sectional multicentre study | 6,698 | - Consecutive patients admitted with acute ischemic stroke and COVID-19 at 12 stroke centers (9 of which admitted all stroke types, and were used to calculate stroke incidence rate) - Out of 6,698 COVID-19 patients admitted to the 9 centres, 1.3% had stroke (IQR 0.75-1.7%) - Median age of patients with large vessel occlusion (LVO) was 51 (16% <50), and African Americans comprised 28% of the patients at US centers (disproportionate) - 50% of LVO patients were either discharged home or to acute rehabilitation facilities. |
| Bronx, USA^99^ | Retrospective cohort study | 4,711 | - Examined records of all COVID-19 patients admitted to their hospital system - 55 patients had radiologically confirmed stroke - These patients had higher in-hospital mortality than age/severity-matched controls with COVID-19 but without stroke |
| New York, USA^127^ | Retrospective cohort study | 3,556 | - Consecutive patients with ischemic stroke and/or COVID-19 in New York health system - 32/3,556 (0.9%) hospitalized COVID-19 patients had imaging-proven ischemic stroke - Cryptogenic stroke was more common in patients with COVID-19 (65.6%) as compared to contemporary controls (30.4%, P=0.003) and historical controls (25.0%, P<0.001) - COVID-19 patients with stroke had higher peak D-dimer levels, stroke severity, and mortality than contemporary patients with stroke without COVID-19 - COVID-19 patients with stroke were more likely to be younger men with elevated troponin, higher stroke severity, higher erythrocyte sedimentation rate and mortality than historical controls with ischemic stroke |
| New York City, USA^128^ | Retrospective chart review | 3,334 | - Thrombotic events occurred in 533/3334 (16.0%) hospitalized COVID-19 patients - 207 (6.2%) thrombotic events were venous, 365 (11.1%) thrombotic events arterial - Mortality of COVID-19 patients with thrombotic events was higher than COVID-19 patients without (43.2% and 21% respectively) - Among 829 ICU patients, 29.4% had thrombotic events (13.6% venous, 18.6% arterial) - Among 2505 non-ICU patients, 11.5% had thrombotic event (3.6% venous, 8.4% arterial) |
| New York City, USA^129^ | Retrospective cohort study | 1,916 (+1,486 influenza) | - Retrospective cohort studying 1,916 patients with COVID-19 - 31 (1.6%) had an acute ischemic stroke - In contrast, 3 (0.2%) of 1,486 patients with influenza (controls) had ischemic stroke |
| Albacete, Spain^130^ | Retrospective study | 1,683 | - Consecutive COVID-19 patients hospitalized over 50 days at a single centre - 1.4% (23) of COVID-19 patients developed cerebrovascular disease - 17 patients were classified as cerebral ischemia, 5 as intracerebral haemorrhage, 1 leukoencephalopathy of posterior reversible type - Characteristic radiological pattern was identified in haemorrhage group including subarachnoid haemorrhage, parietooccipital leukoencephalopathy, microbleeds and single or multiple focal haematomas |
| USA, Spain, Egypt, and Romania^131^ | Retrospective cohort study | 1,448 | - Retrospective observational cohort studying 1,448 patients with COVID-19 - 172 (1.13%) COVID-19 patients diagnosed with acute cerebrovascular event; of these, 156 (1.08% of 1,448) had acute ischemic stroke - In-hospital mortality rate for SARS-CoV-2-associated stroke was 38.1% |
| USA (458 hospitals)^132^ | National stroke registry study | 1,143 | - Using Get With The Guidelines–Stroke, identified 41,971 patients with acute ischemic stroke (AIS) between Feb-June 2020 (AIS/COVID-19: 1,143; AIS/no COVID-19: 40,828) - AIS/COVID-19 patients were younger, more likely to be non-Hispanic Black, Hispanic, or Asian, more likely to present with higher NIHSS scores, and higher proportion of LVOs - These patients had longer times to evaluation and treatment, and worse morbidity and mortality |
| Philadelphia, USA^133^ | Retrospective chart review | 844 | - Among 844 inpatients with COVID-19 at 3 Philadelphia hospitals, 20(2.4%) had confirmed ischemic stroke; 8(0.9%) had ICH - Of the ischemic stroke patients, mean age was 64 years, 80% were Black, and 95% had at least one vascular risk factor; median COVID onset-to-stroke time was 21-days - Cardioembolic mechanism in 40%, cryptogenic in 35%, 3/11(27%) with vessel imaging had large vessel occlusion - Newly positive antiphospholipid antibodies in >75% of tested patients - Of ICH patients, 5/8(63%) were lobar, 3(38%) were SAH; 50% were on ECMO |
| New York City, USA^134^ | Retrospective case-control | 329 | - Retrospective case-control study including 329 patients admitted for stroke - COVID-19 was present in 38.3% (126/329) of patients - Large vessel occlusion was present in 31.7% of patients with COVID-19, in comparison to 15.3% of control patients without COVID-19 - Small vessel occlusion was present in 15.9% of patients with COVID-19 compared with 15.3% of patients without COVID-19 |
| Wuhan, China^135^ | Retrospective cohort study | 219 | - Among 219 patients with COVID-19, 10 (4.6%) developed acute ischemic stroke and 1 (0.5%) had intracerebral haemorrhage - Those with new stroke were older, more likely to have severe COVID-19, cardiovascular risk factors, and increased C-reactive protein and D-Dimer - 6 (54.5%) patients with stroke died within three months |
| Strasbourg, France^136^ | Multicentre prospective cohort study | 150 | - All patients referred to 4 ICUs for ARDS with prospective data collection - Propensity score matching performed to analyze occurrence of thromboembolic events between non-COVID-19 ARDS and COVID-19 ARDS patients. - Clinically-relevant thrombotic complications in 11.7% COVID vs 2.1% non-COVID cases - Strokes in 2/150 COVID-19 cases vs 1/233 non-COVID-19 cases (controls) - Most patients (> 95%) had elevated D-dimer and fibrinogen. |
| Italy^118^ | Retrospective, single-center cohort study | 56 | - 56 positive COVID-19 patients, 117 uninfected patients (controls) admitted for neurological diagnoses (or to a neuro-COVID unit) - Stroke in 35/56 COVID-19 patients vs 50/117 uninfected patients - Stroke patients with COVID-19 and without COVID-19 had similar characteristics, but COVID-19 had higher modified Rankin Scale scores at discharge |
| New York City, USA^137^ | Case series | 45 | - Retrospective observational case series of 45 emergent large vessel occlusion patients - 53% of patients tested positive for COVID-19 - Those with COVID-19 were younger (59 ± 13 vs 74 ± 17 for COVID-negative patients) |
| New York City, USA^138^ | Case series | 33 | - 33 COVID-19 positive patients with neuroimaging-documented intracranial hemorrhage - 5 (15.2%) patients had parenchymal hemorrhages, with 100% mortality rate - 7 (25%) had punctate hemorrhages - 17 (60.7%) had smell-moderate size hemorrhages - 5 (14.3%) had a large single site of hemorrhage |
| Dubai, UAE^139^ | Case series | 22 | - 22 cases of ischemic stroke in patients with PCR-confirmed COVID-19 - Most patients were young males, and two-thirds had ≥1 vascular comorbid conditions - All patients had large territory infarctions, with most having evidence of vessel occlusion - Only 2 patients had evidence of atherosclerotic disease |
| Philadelphia, USA^140^ | Case series | 22 | - Patients developing ischemic or hemorrhagic stroke with COVID-19 in two institutions - Generally younger sample – mean age 59.5-years - 17 cases of ischemic stroke, 3 aneurysm rupture, 2 sinus thrombosis - 16 underwent mechanical thrombectomy with favourable reperfusion, of whom 5 died - Overall, 13.6% had hemorrhagic conversion requiring decompressive surgery, 50% had poor functional status (Rankin 3-6) at discharge, and 36.4% died |
| Baltimore & Boston, USA^141^ | Case series | 20 | - Patients aged 40s-70s who underwent brain autopsy for COVID-19 - In cortical capillaries of 5 patients, megakaryocytes were identified (confirmed with CD61 and CD42b labelling), implicated in endothelial dysfunction - These were distinct from platelet clusters found in postmortem intravascular precipitates - Cortex of two controls without COVID-19 who had hypoxic changes showed no megakaryocytes |
| Piacenza, Italy^142^ | Case series | 19 | - 19 consecutive strokes occurring in COVID-19 patients, mean age 76.1 years - 15 patients had stroke onset during COVID-19 - 4 strokes preceded COVID-19 diagnosis with a mean delay of -2.5 days - Two cases (10.5%) were hemorrhagic and 17 (89.5%) were ischemic - Risk factors were: diabetes 10.5%, hypertension 84.2%, and atrial fibrillation 31.6% |
| Germany^143^ | Retrospective multicentre study | 18 | - 18 patients had evidence of acute intracranial bleeding within 11 days of admission - 6 patients had parenchymal hemorrhage, 11 had subarachnoid hemorrhage, 1 had subdural hemorrhage, 3 presented with intraventricular hemorrhage |
| New York City, USA^144^ | Case series | 16 | - Patients with COVID-19 and ischemic stroke admitted to two New York centres - Age ranged between 27 and 82; 81% had vascular risk factors - Eight had large vessel occlusion (LVO) with median age 55 years, all were male - Eight had non-LVO syndromes, median age 65.5 years and most were female (62.5%) - Both groups were 50% African Americans and 37.5% South Asian - The most common etiology was cryptogenic (6 patients) followed by small vessel occlusion (3 patients) and undetermined-unclassified (3 patients) |
| Boston, USA^145^ | Retrospective cohort study | 16 | - 16 consecutive patients with severe COVID-19 undergoing MRI for coma or focal deficits - 11 patients (69%) had punctate and linear SWI lesions in the subcortical and deep white matter; 8 (50%) had >10 SWI lesions and 4 (25%) had corpus callosum lesions - Brain autopsy in one patient revealed that SWI lesions corresponded to perivascular and parenchymal petechial hemorrhages and microscopic ischemic lesions - Distribution of lesions is similar to that seen in patients with hypoxic respiratory failure, sepsis, and disseminated intravascular coagulation - Single centre study with small sample |
| Bethesda, USA^146^ | Case series (autopsy) | 16 | - Convenience sample of 16 patients who died of COVID-19 - Multifocal microvascular injury was observed in the brain and olfactory bulbs by means of MRI, histopathological evaluation, and immunohistochemical analysis of corresponding sections, without evidence of viral infection - Punctate hyperintensities on MRI in 9 patients corresponded to areas of microvascular injury and fibrinogen leakage; hypointensities in 10 patients corresponded to congested vessels and linear hypointensities to microhemorrhages |
| NC and NY, USA^147^ | Case series | 10 | - 10 hospitalized patients with confirmed COVID-19 who developed severe neurovascular complications (one hemorrhage, two hemorrhagic infarcts, rest multiple infarcts) - Five patients (50%) died following critical illness and cerebral edema |
| **Encephalopathy or encephalitis** | | | |
| Multi-national (but primarily USA)^97^ | Retrospective cohort study | 236,379 | - Analysis of TriNetX database including matched controls with influenza and other respiratory infections (n=236,038 for controls) – see Table 4 for more details - Estimated 6-month incidence encephalitis was 0.10% (95%CI 0.08-0.13), with dementia identified in 0.67% (0.59-0.75) - In the subgroup with intensive care admission, estimated incidence of encephalitis was 0.35% (0.19-0.64) and that of dementia was 1.74% (1.31-2.30) - Encephalitis more common in patients who had COVID-19 than in those who had influenza (hazard ratio [HR] 1.70, 95% CI 1.04–2.78) and those who had other respiratory tract infections (1.41, 1.03–1.92); same for dementia (vs influenza: 2.33, 1.77-3.07; vs others: 1.71, 1.50-1.95) - HRs were higher in patients who had more severe COVID-19 |
| New York City, USA^148^ | Retrospective study | 115 | - 115 COVID-19 patients that had an MRI - 30.4% had leukoencephalopathy and/or microbleeds - Patients with leukoencephalopathy and/or microbleeds had more complications such as ventilator support duration, moderate-severe ARDS, higher mortality (20% vs. 9%) |
| Milan, Italy^149^ | Cross-sectional study | 18 | - Consecutive COVID-19 patients, admitted, evaluated with EEG - Symptoms included transient loss of consciousness, seizures/spasms, delirium, coma - Generalized EEG slowing in 88.9%, bifrontal predominance in 55.6% - Only two had epileptiform discharges; no seizures were detected |
| **Other Neuropsychiatric Disorders** | | | |
| Multi-national (but primarily USA)^97^ | Retrospective cohort study | 236,379 | - Analysis of TriNetX database including matched controls with influenza and other respiratory infections (n=236,038 for controls) – see Table 4 for more details - Estimated 6-month incidence of mood, anxiety, or psychotic disorder: 24.0% (95%CI 23.6-24.4) - In the subgroup with intensive care admission, estimated incidence was 27.8% (26.3-29.3) - These disorders were more common in patients who had COVID-19 than in those who had influenza (HR 1.46, 95% CI 1.43–1.50) and those who had other respiratory tract infections (1.20, 1.18–1.23). HRs were higher in patients who had more severe COVID-19 |
| USA^150^ | Cross-sectional survey | 3,900 | - US adult participants surveyed between May 2020 and Jan 2021 in 8 waves of an internet-based non-probability survey conducted by Qualtrics with multiple panels of respondents, including the Patient Health Questionnaire-9 for screening for depression - Among 82,319 respondents who completed the survey, 3,904 people reported prior COVID-19 - 52.4% met criteria for moderate or greater symptoms of major depression (PHQ-9≥10) - Symptoms were more likely among younger respondents and men, and among those with greater self-reported overall COVID-19 severity - Limitations include absence of lab confirmation of infection and reliance on self report, as well as response bias of the survey design |
| Milan, Italy^151^ | Cross-sectional study | 402 | - Adult survivors of COVID-19 (265 men, mean age 58) screened for psychiatric symptoms at 1-month follow-up - 56% scored in pathological range for at least one clinical dimension - 28% screened positive for post-traumatic stress disorder (PTSD), 31% for depression, 42% for anxiety, 20% for obsessive-compulsive symptoms, 40% for insomnia - Besides female sex and prior psychiatric diagnosis, baseline systemic immune-inflammation index predicted higher depression and anxiety scores |
| Indiana, USA^152^ | Retrospective chart review | 144 | - Observational study on consecutive COVID-19 patients admitted to the ICU - 73.6% (106/144) patients had delirium; median duration of 5 days - 76.4% (110/144) patients had delirium or were in a coma; median duration of 7 days - 63% of patients were positive for delirium on the first CAM-ICU assessment with the median score being 6, representing severe delirium - 26.4% mortality in patients with delirium, 15.8% mortality in patients without delirium |
| United Kingdom^153^ | Cross-sectional study | 125 | - Online network of major UK neuroscience bodies compiled cases - 23/39 (59%) patients with altered mental status met definitions for psychiatric diagnoses - 21 (92%) of these psychiatric diagnoses were new diagnoses - 10 (43%) of 23 patients with neuropsychiatric disorders had new-onset psychosis - 6 (26%) of 23 patients had neurocognitive (dementia-like) syndrome - 4 (17%) of 23 patients had an affective disorder |
| Milan, Italy^154^ | Retrospective single-centre | 57 | - Retrospective single-centre study with 57 elderly residents that tested positive for COVID-19 - 21/57 subjects had delirium-onset COVID-19 - Subjects with delirium were older (85 ± 4 years-old) - Delirium-onset COVID-19 was strongly associated with the higher mortality |
| **Seizures** | | | |
| China^155^ | Retrospective multicenter study | 304 | - Retrospective multicenter study of COVID-19 positive patients, 108 with severe disease - None in the cohort had known history of epilepsy - No acute symptomatic seizures nor status epilepticus was observed - 2 patients had seizure-like symptoms - 84 (27%) had brain insults or metabolic imbalances known to increase risk of seizures |
| New York, USA^156^ | Case series | 28 | - Patients with suspected COVID-19 (6 later tested negative) who had EEGs for acute encephalopathy or seizure-like events - Sporadic epileptiform discharges were present in 40.9% of COVID‐19‐positive and 16.7% of COVID‐19‐negative patients; frontal sharp waves were reported in 88.9% - No electrographic seizures were captured, but most received antiepileptic drugs |
| **Movement Disorders** | | | |
| Multi-national (but primarily USA)^97^ | Retrospective cohort study | 236,379 | - Analysis of TriNetX database including matched controls with influenza and other respiratory infections (n=236,038 for controls) – see Table 4 for more details - Estimated 6-month incidence parkinsonism was 0.11% (95%CI 0.08-0.14) - In the subgroup with intensive care admission, estimated incidence was 0.26% (0.15-0.45) - Parkinsonism more common in patients who had COVID-19 than in those who had influenza (hazard ratio [HR] 1.42, 95% CI 0.75–2.67) and those who had other respiratory tract infections (1.45, 1.05–2.00) - HRs were higher in patients who had more severe COVID-19 |
| Multiple centres (aggregated in Calgary, Canada)^157^ | Systematic review of case reports/series | 51 | - Cases of myoclonus or ataxia associated with COVID-19 identified from 32 publications (with additional case added by authors) - Mean age 59.6 years, 21.6% female - Myoclonus was multifocal/generalized and acute onset, usually within 1-month of COVID symptoms, occurring alone (46.7%), with ataxia (40.0%) or cognitive changes (30.0%) - Most improved in 2 months; treatment included anti-epileptic drugs or immunotherapy - Ataxia had acute onset, usually within 1 month, but sometimes an initial symptom - Ataxia occurred with cognitive changes (45.5%), myoclonus (36.4%), or a Miller Fisher syndrome variant (21.2%) - Most ataxia cases improved in 2 months, spontaneously or with immunotherapy |
| **Headaches** | | | |
| Valladolid, Spain^158^ | Retrospective cohort study | 576 | - Analyzed symptoms and laboratory abnormalities associated with headache among consecutive patients admitted to the hospital with confirmed COVID-19 - Headache in 23.7%, associated with lower mortality - Anosmia, myalgia, female sex and fever associated with higher odds of headache - Younger age, lower score on modified Rankin scale, increased C-reactive protein and D-dimer, abnormal platelet titres, and lymphopenia had lower odds of headache |
| Spain^159^ | Cross-sectional survey | 112 | - Survey of Spanish healthcare professionals infected with SARS‐CoV‐2 who presented with headache during the course of the disease (major concern of selection bias) - History of migraine in 17.9%, tension-type in 7.1%; 73.2% had no headache history - Headache presented independently of fever, around third day after symptom onset - Headache heterogeneous but often holocranial, hemicranial, or occipital, pressing, and worsening with physical activity or head movements - Prior migraine associated with higher higher frequency of pulsating headache |
| Wuhan, China^160^ | Retrospective case series | 62 | - 34% of COVID-19 patients had headache |
| Ankara, Turkey^161^ | Case series | 13 | - 13 patients with mild COVID-19 who developed headaches requiring consultation - Headache was the only symptom in 3 patients, emerged as an early symptom for rest - Severe, rapid onset, unrelenting with migraine-like features (most had no prior history) - Anosmia, diarrhea, loss of appetite and weight also occurred - Headache lasted up to 3 days in 70%, resolved in all patients within 2 weeks |
| **Other neurological, neuropathological, and neuroimaging features** | | | |
| Stockholm, Sweden^162^ | Retrospective chart review | 185 | - Included all consecutive adult hospitalized patients with PCR-positive for SARS-CoV-2 undergoing neuroimaging at the centre with systematic re-evaluation by 12 readers - Intra-axial susceptibility abnormalities most common finding in those undergoing MRI (29/39, 74%) with ovoid shape and predilection for corpus callosum and juxtacortical areas; some ischemic and macro-hemorrhagic lesions were also seen - 44% of patients had leukoencephalopathy, one with cytotoxic corpus callosum lesion - Olfactory bulb signal abnormalities in 18%, and parenchymal, leptomeningeal, cranial or spinal nerve enhancement in about 10-15% of cases - In one patient, regression of leukoencephalopathy seen on MRI followup, and in another, progressive leptomeningeal enhancement seen, suggesting dynamic processes |
| Barcelona, Spain^163^ | Cross-sectional study | 103 | - 103 COVID-19 patients who underwent neuroimaging at a single centre - Patients underwent neuroimaging for non-focal neurologic symptoms, signs of stroke, focal neurologic symptoms, post-sedation encephalopathy, and seizures - Ischemic stroke identified in 13 patients, hemorrhage in 7 patients - No specific neuroimaging findings in most others, so no conclusions could be drawn concerning concrete associations between neuroimaging and COVID-19 |
| Paris, France^164^ | Cross-sectional study | 73 | - 73 COVID-19 patients with neurological manifestations and brain MRI - 43 presented pathological MRI findings 2-4 weeks after symptom onset; 17 of these had acute ischemic infarct, 1 venous thrombosis, 8 with multiple microhemorrhages, 22 with perfusion abnormalities, 3 with cytotoxic lesions of the corpus callosum - CSF negative for SARS-CoV-2 in all patients |
| Madrid, Spain^165^ | Cross-sectional study | 55 | - Patients hospitalized for COVID-19 were tested for a cross-reactive antibody by immunofluorescence: 12 positive compared to 43 negative - Antibody-positive patients were more likely to have confusion, anosmia, or peripheral neuropathies (33.3% vs 9.8%) as well as DVT/PE (66.7% vs 34.1%) |
| Turkey, USA^166^ | Retrospective study | 50 | - Brain MRI performed in 27 (54%) of 50 COVID-19 patients with neurologic symptoms - 12/27 patients had acute MRI findings - 10/27 had cortical FLAIR signal abnormality, 3 patients had subcortical and deep white matter signal abnormality |
| Hamburg, Germany^167^ | Post-mortem case series | 43 | - Patients with positive SARS-CoV-2 by quantitative RT-PCR and adequate post-mortem samples - Neuropathological workup included histological and immunohistochemical staining for activated astrocytes, activated microglia, and cytotoxic T lymphocytes in the olfactory bulb, basal ganglia, brainstem, and cerebellum, and for presence of SARS-CoV-2 - Fresh territorial ischaemic lesions were seen in six (14%) patients - 37 (86%) patients had astrogliosis in all assessed regions. Activation of microglia and infiltration by cytotoxic T lymphocytes was most pronounced in the brainstem and cerebellum, and meningeal cytotoxic T lymphocyte infiltration was seen in 34 (79%) patients - **SARS-CoV-2 was detected in the brains of 21 (53%) of 40 examined patients**, with SARS-CoV-2 viral proteins found in cranial nerves originating from the lower brainstem and in isolated cells of the brainstem. The presence of SARS-CoV-2 in the CNS was not associated with the severity of neuropathological changes. |
| France^168^ | Case series | 37 | - 37 severe COVID-19 patients with neurological manifestations - 73% had alteration of consciousness, 32% had confusion, 19% had agitation - 54% had intracerebral hemorrhagic lesions with more severe clinical presentation - MRI findings included: 43% signal abnormalities in the medial temporal lobe, 30% non-confluent multifocal white matter hyperintense lesions on FLAIR and diffusion sequences, with associated hemorrhagic lesions, and 24% extensive and isolated white matter microhemorrhages - **One patient was positive for SARS-CoV-2 RNA in CSF** |
| Switzerland^169^ | Case series | 31 | - Cytological examination of CSF was performed for 33 CSF samples from 31 patients and the blood-brain barrier integrity evaluated using albumin quotient - SARS-CoV-2 RNA not detected in CSF; observed signs of blood-brain barrier disruption |

**Supplementary Table 6. Relevant case reports and small case series (<10 patients) on central nervous system (CNS) manifestations of COVID-19.** Studies are shown in descending order by sample size. For the handful of reports where SARS-CoV-2 PCR and/or antibodies were positive in the cerebrospinal fluid (CSF), the locations are bolded and indicated by an asterisk (*).

| **Location** | **Study Type** | **Number of patients** | **Key findings** |
| --- | --- | --- | --- |
| **Stroke with or without coagulopathy** | | | |
| Madrid, Spain^170^ | Case series | 8 | - All patients met diagnostic criteria for severe COVID-19, none with history of stroke - Ischemic stroke occurred a median of 11.5 days after the onset of COVID-19 symptoms - Stroke occurred a median of 5.5 days after admission among hospitalized patients - All of them were large artery infarctions |
| Italy^171^ | Retrospective case series | 6 | - 5 men and 1 woman, from 57 to 82-years presented with strokes (4 ischemic and 2 hemorrhagic) - All patients but 1 had pre-existing vascular risk factors - COVID-19-related pneumonia was severe in 5/6 cases - 5/6 died and the remaining 1 was severely neurologically affected |
| London, United Kingdom^172^ | Case series | 6 | - All were older than 50-years with large vessel occlusion and D-dimer ≥1000µg/L - Three patients had multi-territory infarcts, two had concurrent venous thromboses - Two patients had strokes despite therapeutic anticoagulation |
| Tehran, Iran^173^ | Case series | 6 | - Six patients younger than 55 presented diagnosis of stroke and confirmed COVID-19 - The most involved area was the middle cerebral artery (5/6 patients) - One patient’s stroke involved basal ganglia |
| New York City, USA^174^ | Case series | 5 | - Five patients younger than 50-years with large vessel stroke seen over just two weeks, vs an average of 0.73 young strokes seen every two weeks at that centre in the preceding year^174^ - Two patients did not have any COVID-19 symptoms, and one just had lethargy |
| London, UK^175^ | Case series | 5 | - Five consecutive cases of predominantly lobar COVID-19-associated intracerebral haemorrhage - Patients were typically relatively young with a severe, prolonged inflammatory prodrome - Four patients were on prophylactic heparin or low-molecular weight heparin, one on warfarin |
| Granada, Spain^176^ | Case series | 4 | - Four patients with ischaemic stroke and COVID-19 - Two patients presented cortical infarcts and no relevant arterial or cardioembolic disease - Other two patients were of advanced age and presented cardioembolic ischaemic stroke |
| Newark, USA^177^ | Case series | 4 | - 4 COVID-19 patients 37 to 68-years-old with varying COVID-19 severities presented ischemic strokes |
| Sakarya, Turkey^178^ | Case series | 4 | - Four patients from 45 to 77-years-old reported stroke cases with simultaneously diagnosis of COVID-19, presenting common symptoms of COVID-19 - Three patients had elevated D-dimer levels - Two patients had high C-reactive protein levels |
| Beijing, China^179^ | Case series | 3 (all critically ill) | - Antiphospholipid antibodies identified in the context of various combinations of limb or digital ischemia and bilateral infarcts in multiple territories - All 3 had thrombocytopenia and elevated prothrombin time, fibrinogen, and D-dimer, with anticardiolipin IgA and anti–β_2_-glycoprotein I (IgA and IgG) antibodies |
| Rio de Janeiro, Brazil^180^ | Case series | 3 | - Three critically ill COVID-19 patients, aged 40-60s, who all developed ARDS and acute renal failure - All developed catastrophic intracerebral hemorrhages (ICH) |
| Sari, Iran^181^ | Case series | 3 | - Three COVID-19 patients (2 females, 1 male) 55 to 88 years-old with ischemic stroke - One patient had fever along with stroke symptoms - One patient had dry cough 3-days before stroke symptoms - One patient had fever and respiratory problems 3-days after admission |
| Philadelphia, USA^182^ | Case series | 2 | - Two patients on veno-venous extracorporeal membrane oxygenation (ECMO) for severe COVID-19 - Developed devastating intracranial hemorrhage |
| New York, USA^183^ | Case series | 2 | - Two men (aged 62 and 74) admitted with COVID-19 and anticoagulated empirically - Both developed catastrophic intracranial hemorrhage and cerebral edema |
| New York, USA^184^ | Case series | 2 | - Two patients (49-year-old male and 53-year-old female) with COVID-19 presented with acute strokes - Imaging features in both patients were consistent with acute thrombosis in the ipsilateral common carotid artery bifurcation - Neither patient showed evidence of intracranial or extracranial atherosclerotic disease |
| New York City, USA^185^ | Case series | 2 | - Two female patients 33 and 77-years-old presented with stroke secondary to vessel thrombosis - They were later found to have COVID-19 with only mild respiratory symptoms |
| New York City, USA^186^ | Case series | 2 | - Two patients (61-year-old male and 34-year-old female) with COVID-19 diagnosis ranging from 7 to 10-days experienced painless vision loss for 2 days - Neuroimaging showed thrombotic events occurred leading to a devastating visual outcome secondary to occipital lobe ischemia |
| PennsylvaniaUSA^187^ | Case series | 2 | - Two COVID-19 patients, 31-year-old male and 62-year-old female, who had subarachnoid haemorrhage and ischaemic stroke respectively - CSF repeatedly negative for SARS-CoV-2 |
| London, UK^188^ | Case series (autopsy) | 2 | - Autopsy of two patients who died of COVID-19 showing multiple infarcts - 50-year-old male with cardiac arrest who had multifocal brain infarcts in watershed regions, as well as large right MCA and bilateral PCA infarcts - 60s-year-old female with multiorgan failure who had bilateral pallidal infarcts, cortical and white matter microbleeds, lymphohistiocytic inflammation with corresponding leptomeningeal hyperintensity (MRI) |
| New Delhi, India^189^ | Retrospective chart review | 2 | - Reviewing 25 patients with stroke admitted the previous month, found 14(56%) had ICH - Two patients with ICH were SARS-CoV-2 positive and they had no or mild respiratory symptoms and had higher occurrence of renal dysfunction. - Study highly limited by small convenience sample and limited data |
| Detroit, USA^190^ | Case report | 1 | - 72-year-old female with medical history for hypertension, diabetes, chronic renal failure, and gout presented with 3-wek history of progressive cough, general myalgia and shortness of breath - Tested positive for COVID-19 - Developed severe sepsis, septic shock, micro and macro-vascular thrombotic phenomena, leading to multi-vessel stroke |
| Flint, USA^191^ | Case report | 1 | - 31-year-old COVID-19 positive female with no stroke risk factors presented with cortical stroke - Correlation between cytokine release, encephalopathy, and the onset of stroke symptoms |
| Hubei, China^192^ | Case report | 1 | - 79-year-old male presented with 1-day history of right limb weakness and 1-week history of slight cough - RT-PCR was positive for SARS-CoV-2 - Treated with clopidogrel and atorvastatin for acute ischemic stroke |
| Lecco, Italy^193^ | Case report | 1 | - 47-year-old male with 10-day history of fever and the subsequent appearance of dyspnea, as well as a medical history of allergic asthma - Tested positive for SARS-CoV-2 and did not have any stroke risk factors present - 5-days after hospitalization, developed transient episode of paresthesia in the left hand and loss of vision, lasting for 2 minutes - The next day experience 2 similar episodes of 15 and 30 minutes |
| Los Angeles, USA^194^ | Case report | 1 | - Patient presented with acute chest pain, diaphoresis, and hypotension - RT-PCR tested positive for SARS-CoV-2 - Intubated due to hypoxemic respiratory failure and extubated on day 4 - On day 5, large vessel occlusion stroke syndrome |
| Reggio Emilia, Italy^195^ | Case report | 1 | - 33-year-old male presented with acute onset of occipital headache, retching and balance disorder - 10-days prior complained of cough and occasional fever - CT-scans suggested intraluminal thrombosis - Acute cerebrovascular disorder as presenting early onset of COVID-19 |
| Reno, USA^196^ | Case report | 1 | - 58-year-old female with a history of a cerebral artery ischemic stroke presented with acute-onset dysarthria, left-sided droop and left-sided hemiparesis of 1-hour duration - Was later found to be COVID-19; COVID-19 presenting solely with focal neurological manifestations |
| Strasbourg, France^197^ | Case report | 1 | - 65-year-old woman newly diagnosed with immune thrombocytopenic purpura (ITP), with a small right frontal subarachnoid hemorrhage on CT - Resolved 96 hours after treatment with prednisone, platelet transfusion, and eltrombopag |
| USA^198^ | Case report | 1 | - Four patients ranging from 73 to 83-years-old presented with radiographic evidence of acute stroke and PCR-confirmed COVID-19 |
| New York City, USA^199^ | Case report | 1 | - 55-year-old male presented with left wrist drop - Had carotid artery thrombosis and later found to be COVID-19 positive |
| Wuhan, China^200^ | Case report | 1 | - 68-year-old male on warfarin presented with fever, cough and fatigue for 8 days, RT-PCR positive - CT Head for altered level of consciousness showed right temporal-occipital, left frontal-occipital-parietal hemorrhage with intraventricular extension, subarachnoid hemorrhage, brain herniation (died on day-26) |
| Madrid, Spain^201^ | Case report | 1 | - 67-year-old male found dazed and confused by his neighbours, diagnosed with SARS-CoV2 pneumonia - After 15 days, had persistent confusion, gait ataxia, dysarthria, partial cortical blindness and anosognosia with visual confabulation, optic ataxia, simultagnosia, and mild left hemihypoesthesia - MRI Brain showed strokes in the posterior segment of the right MCA, left PCA, and right superior cerebellar artery with cortical laminar necrosis - CSF showed elevated WBC (lymphocytes 90%) and protein, normal glucose, and negative meningitis/encephalitis multiplex PCR assay (SARS-CoV-2 not tested in CSF), + oligoclonal bands |
| Madrid, Spain^202^ | Case report | 1 | - 19-year-old female with SARS- CoV-2 infection presenting with thalamic hemorrhage and acute cognitive impairment - Angiography showed features of Moyamoya angiopathy – ? unmasked by COVID-19 |
| Sari, Iran^203^ | Case report | 1 | - 54-year-old female with loss of consciousness (Glasgow Coma Scale score of 10) - Brain CT and MRI showed bilateral subacute basal ganglia hemorrhage - Lung CT showed classic findings of COVID-19 |
| Orlando, USA^204^ | Case report | 1 | - 66-year-old female presented in cardiac arrest with return of spontaneous circulation - Found to have subarachnoid hemorrhage and later tested positive for COVID-19 |
| Daegu, Korea^205^ | Case report | 1 | - 53-year-old female with sudden left hemiparesis, had otherwise asymptomatic COVID-19 infection - CT showed right external capsule and putamen ICH with intraventricular extension |
| Lillie, France^206^ | Case report | 1 | - 65-year-old man with typical COVID-19 respiratory presentation - Remained drowsy and unarousable - CT/MRI showed multiple white matter, basal ganglia, cerebellar, and bilateral globus pallidus hemorrhagic lesions and extensive diffusion-restricting (ischemic) lesions in the centrum semiovale, corpus callosum, basal ganglia, and cerebellum, with patchy/punctuate enhancement (?vasculitis) |
| Braga, Portugal^207^ | Case report | 1 | - 42-year-old male with fever, asthenia, myalgias, dry cough, hyposmia, RT-PCR+ for SARS-CoV-2 - One week later, presented with altered mental status, slowed movements, apathy - Neuro exam showed dysexecutive syndrome, perseveration, mild dysphonia/dysphagia - MRI Brain showed multiple lesions in deep and subcortical white matter bilaterally, as well as thalami, basal ganglia, and pons, some with diffusion restriction - Irregularity of left P3 seen on MRA, and irregularity of lenticulostriate artery seen on digital subtraction angiography – COVID-19 associated CNS vasculopathy diagnosed - CSF showed mildly elevated proteins, negative for SARS-CoV-2 or other viruses - Treated with IVIG initially, then high-dose IV methylprednisolone for 5-days, then tapered, plus aspirin - Discharged after 13-days with oral prednisolone with no new MRI lesions |
| **Cerebral Venous Thrombosis (CVT)** | | | |
| Shiraz, Iran^208^ | Case series and literature review | 6 (28 found on review) | - Patients with documented COVID-19 and clinical and radiological features of CVT, aged 31-62 - 5 had clinical manifestations of CVT and COVID-19 simultaneously, 3 had predisposing factors - 4 patients died; review indicated COVID-19 patients with CVT had higher mortality than those without. |
| USA^209^ | Case series | 3 | - Three patients younger than 41-years-old with COVID-19 had neurologic findings related to CVT - The median time from COVID-19 symptoms to a thrombotic event was 7 days (rang, 2-7 days) |
| Paris, Italy^210^ | Case series | 2 | - Two patients 62 and 54-year-old females positive for COVID-19 developed cerebral venous thrombosis |
| Iran^211^ | Case report | 1 | - 65-year-old previously healthy male complained of loss of consciousness, upward gaze, tongue biting - Neuroimaging showed hemorrhagic infarct - Lymphopenia and low oxygen saturation, suggesting COVID-19; RT-PCR positive for SARS-CoV-2 - No CVT susceptible conditions and no common COVID-19 symptoms |
| Italy^212^ | Case report | 1 | - 81-year-old male presented with interstitial pneumonia with respiratory distress; RT-PCR tested positive for SARS-CoV-2 - Developed sudden impairment of consciousness, leading to coma - Neuroimaging suggested concomitant venous and arterial thrombosis of the brain |
| Lecco, Italy^213^ | Case report | 1 | - 54-year-old male presented with 2-week history of cough, fatigue and fever - Nasopharyngeal swab positive for SARS-CoV-2 - Brain CT showed cerebral venous thrombosis |
| USA^214^ | Case report | 1 | - 29-year-old female, with no significant past medical history, presented with new onset seizures and 1-week history of headaches, fever, cough and shortness of breath - Neuroimaging revealed hemorrhagic venous infarction - CVT as early presentation of COVID-19 |
| Iran^215^ | Case report | 1 | - 43-year-old woman with headache, seizure, drowsiness - MRI/MRV showed thrombosis of superior sagittal sinus, internal cerebral vein, straight sinus, and hyperintensities in bilateral thalami, basal ganglia, and left temporal lobe (venous infarction) - CSF unremarkable, elevated liver enzymes - Treated with low molecular weight heparin – improved clinically after 8 days |
| Rome, Italy^216^ | Case report | 1 | - 44-year-old woman with COVID-19 presented with worsening dyspnea, headache, altered mental status, aphasia, and right hemiparesis - Imaging showed superior vena cava, pulmonary artery, and deep intracerebral venous thrombosis. |
| Paris, France^217^ | Case report | 1 | - 72-year-old man with sudden left hemiparesis, altered mental status, refractory status epilepticus - COVID-19 positive, found to have deep cerebral vein thrombosis - Complicated by hemorrhagic venous infarction - CSF showed 30 WBC/mm3 but otherwise unremarkable, negative for SARS-CoV-2 |
| **Encephalopathy, encephalitis, or other neuropsychiatric presentations** | | | |
| Geneva, Switzerland ^218^ | Case series | 9 | - 9 COVID-19 patients presented with delayed recovery of consciousness or considerable agitation - Common MRI finding amongst all patients was presence of microbleeds in unusual distribution with a specific predilection for the corpus callosum - 5/9 had microbleeds in the internal capsule and 5/9 also had microbleeds in middle cerebellar peduncles |
| Nijmegen, The Netherlands^219^ | Case series | 7 | - COVID-19 patients with prolonged but reversible unconsciousness after severe respiratory failure - After cessation of sedatives, all patients showed a prolonged comatose state - Neuroimaging did not show signs of devastating injury, CSF analyses were normal - Clinical pattern of awakening started with early eye opening without obeying commands and persistent flaccid weakness in all cases - Time between cessation of sedatives to the first moment of being fully responsive with obeying commands ranged from 8 to 31 days. |
| Gothenburg, Sweden^220^ | Case series | 6 | - Patients hospitalized with COVID-19 and neurologic symptoms who had undergone lumbar puncture - Assessed CSF SARS-CoV-2 RNA along with CSF biomarkers of intrathecal inflammation (CSF white blood cell count, neopterin, β2-microglobulin, and immunoglobulin G index), blood-brain barrier integrity (albumin ratio), and axonal injury (CSF neurofilament light chain protein [NfL]) - Neurologic symptoms and signs included features of encephalopathies (4 of 6), suspected meningitis (1 of 6), and dysgeusia (1 of 6) - SARS-CoV-2 RNA was detected in the plasma of 2 patients and in CSF at low levels in 3 patients in one but not in a second real-time PCR assay - CSF neopterin and β2-microglobulin were increased in all, whereas median immunoglobulin G index, albumin ratio, and CSF white cell count were normal in all; CSF NfL was elevated in 2 patients - Overall an unusual pattern of marked CSF inflammation in which soluble markers were increased but white cell response and other immunologic features typical of CNS viral infections were absent, but they could not convincingly detect SARS-CoV-2 as the underlying driver of CNS inflammation |
| Geneva, Switzerland ^221^ | Case series | 5 | - Five patients intubated because of COVID-19 related acute respiratory distress syndrome - Presented with pathological slow recovery of consciousness, responsive to high dose glucocorticoids - MRI consistent with endotheliitis |
| Toronto, Canada^222^ | Case series | 4 | - Assessed neuroimaging findings of 4 COVID-19 patients with abnormal mental status, deranged coagulation parameters, and elevated D-dimer levels - CT/MR imaging revealed common pattern of multifocal subcortical/cortical petechial-type hemorrhages, consistent with thrombotic microangiopathy |
| Milan, Italy^223^ | Case series | 4 | - Four COVID-19 patients with subacute encephalopathy - Brain MRI showed a multifocal involvement of the cortex in all cases located in the parietal, occipital and frontal regions |
| New York City, USA^224^ | Case series | 4 | - Four patients from 23 to 73-years-old critically ill patients presenting with leukoencephalopathy following COVID-19 infection |
| USA^225^ | Case series | 4 | - Four patients ranging from 64 to 74-years-old presented with acute respiratory distress syndrome, admitted to the ICU with mechanical ventilation - Persistent confusion, lethargy, new focal neurological deficits, suggested PRES - Brain imaging confirmed presence of cerebral vasogenic edema - Improved symptoms with blood pressure control |
| Atlanta, USA^226^ | Case series | 3 | - Three COVID-19 patients developed encephalopathy and encephalitis - All 3 patients had increased CSF levels of anti-S1 IgM - SARS-CoV-2 was not found in any CSF sample |
| ***Amiens, France**^227^ | Case series | 2 | - Two patients, a middle-aged female and male, positive for SARS-CoV-2 had brain MRIs consistent with encephalopathy - SARS-CoV-2 antibodies detected in both patients’ CSF samples |
| Paris-Saclay, France^228^ | Case series | 2 | - Two men, aged 49 and 51 years, with acute encephalopathy and rapid clinical deterioration - Both recently tested positive for COVID-19 on nasopharyngeal swab - Brain MRI showed a cytotoxic-pattern lesion of the splenium of the corpus callosum was found, with T2-FLAIR hyperintensity and restricted diffusion - This is typically reversible and the underlying mechanism is thought to rely on the vulnerability of the splenium of the corpus callosum to cytokinopathy. |
| Cambridge, USA^229^ | Case series | 2 | - Two patients, 58-year-old male and 67-year-old female, positively diagnosed with COVID-19 required mechanical ventilation - MRI revealed findings compatible with posterior reversible encephalopathy syndrome (PRES) |
| USA^230^ | Case series | 2 | - Two hospitalized patients, 48-year-old male and 67-year-old female, with COVID-19 - Brain imaging revealed hemorrhagic posterior reversible encephalopathy syndrome |
| Marseille, France^231^ | Case series | 2 | - Two patients who underwent 18F-FDG PET studies in post-viral stage of COVID-19 - One had anosmia and the other had delayed onset of a pain syndrome - Hypometabolism of olfactory/rectus gyrus seen in both; patient with pain also had hypometabolism in the amygdala, hippocampus, parahippocampus, cingulate cortex, pre-/post-central gyrus, thalamus/hypothalamus, cerebellum, pons and medulla |
| Nottingham, UK^232^ | Case series | 2 | - Two patients 46-year-old male and 79-year-old female with severe COVID-19 with acute onset of altered mental status and delirium with normal respiration in the first 2-days |
| ***Erlangen, Germany^233^** | Case report | 1 | - 53-year-old man with fever and increasing headache - Neuroimaging and CSF revealed meningeosis carcinomatosa - SARS-CoV-2 infection of the CNS was also confirmed by detection of viral RNA in 2 independent CSF samples and SARS-CoV-2–specific intrathecal IgG antibody synthesis |
| ***Beijing, China**^234^ | Case report | 1 | - 56-year-old patient with pneumonia due to COVID-19 who developed symptoms of encephalitis - SARS-CoV-2 PCR positive in the CSF |
| United Arab Emirates (UAE)^235^ | Case report | 1 | - 43-year-old male diagnosed with COVID-19 required intubation 1-week after - Brain MRI showed features of encephalopathy |
| Boca Raton, USA^236^ | Case report | 1 | - 74-year-old man initially presented with fever/cough, and returned in 24h with encephalopathy - CT head showed old known stroke, EEG showed slowing, and CSF was bland |
| Boca Raton, USA^237^ | Case report | 1 | - 72-year-old male initially presented with fever and dry cough - Developed hypoxemic respiratory failure and intubated - MRI revealed small cortical posterior left parietal infarct - SARS-CoV-2 PCR tested positive - EEG consistent with encephalopathy, CSF bland |
| Brescia, Italy^238^ | Case report | 1 | - 36-year-old male patient presented with 2 days of fever, headache, body pain, cough, diarrhea and vomiting. The patient was negative for SARS-CoV-2 and diagnosed as gastroenteritis and discharged - 4-days later patient went back to hospital complaining of the same symptoms and additionally he presented with drowsiness and appeared mildly confused. - COVID-19 swab was repeated and resulted positive - Bilateral supratentorial leptomeningeal increased enhancement was detected and further supported the diagnosis of COVID-19-related meningoencephalitis. |
| Calgary, Canada^239^ | Case report | 1 | - 69-year-old male with history of coronary artery disease, hypertension and type 2 diabetes mellitus and travel history to USA, presented with acute encephalopathy and respiratory distress - Nasopharyngeal swab for COVID-19 tested positive for SARS-CoV-2, CSF negative - Brain MRI showed multifocal, multicompartmental hemorrhages with peri-hemorrhage vasogenic edema |
| China^240^ | Case report | 1 | - 31-year-old male presenting with 5-day history of fever and headache - Lab tests consistent with encephalitis - Later developed mild encephalitis/encephalopathy with a reversible splenial lesion |
| Istanbul, Turkey^241^ | Case report | 1 | - 38-year-old male patient admitted with a history of fever for 5 days, tested positive for SARS-CoV-2 - After fifth day of ICU, patient complained about vision loss in both eyes. - Brain MRI revealed vasogenic edema similar to posterior reversible leucoencephalopathy (PRES). - Corticosteroid therapy was tapered and on the tenth day neurological examination and neurocognitive assessment were completely normal - Brain MRI performed two weeks later showed complete regression of the lesions |
| Créteil, France^242^ | Case report | 1 | - 69-year-old female presented to stroke unit for 3 generalized seizures, mutism and delirium, and 7-day history of unusual asthenia - SARS-CoV-2 was positive in nasopharyngeal swab and negative in CSF - MRI day 8 confirmed diagnosis of PRES |
| Detroit, USA^243^ | Case report | 1 | - Middle-aged woman with cough, fever, altered mentation - CT/MRI showed hemorrhagic enhancing lesions in the thalami, medial temporal lobes, and subinsular regions, without any arterial or venous occlusions, supportive of acute necrotizing encephalitis |
| France^244^ | Case report | 1 | - 72-year-old male presented with subacute cerebellar syndrome and myoclonus days after general COVID-19 symptoms - Oropharyngeal swab test was positive for SARS-CoV-2 - Brain ^18^F-FDG PET results were compatible with encephalitis - Treatment with steroids allowed a rapid improvement of symptoms |
| London, United Kingdom^245^ | Case report | 1 | - 65-year-old female presented with 1-week history of widespread involuntary movements, diplopia and cognitive decline, having experienced fever, cough and myalgias the week before - Throat and nasopharyngeal swab was positive for SARS-CoV-2, CSF negative for SARS-CoV-2 - Neurological examination revealed widespread, stimulus-sensitive myoclonus involving the tongue and all four limbs - Neurological symptoms persisted for the following week but repeated testing for SARS-CoV-2 was negative, with no symptoms of COVID-19 - Teleconferencing meeting outcome found most likely cause of neurological symptom to be postinfectious immune-mediated encephalitis |
| London, United Kingdom^246^ | Case report | 1 | - 50-year-old female presented with seizures and reduced consciousness 10-days after onset of fever, cough and headache - SARS-CoV-2 nasopharyngeal swab test was positive - MRI on day 6 revealed worsening brain stem swelling with symmetrical hemorrhagic lesions consistent with acute necrotizing encephalopathy |
| Los Angeles, USA^247^ | Case report | 1 | - 41-year-old female with history of diabetes presented with headache, fever and new onset seizure, no respiratory symptoms - Admitted for management of viral meningitis - Experienced worsening encephalopathy - COVID-19 testing positive - Began to improve by day 5 of admission after being administered hydroxychloroquine |
| Madrid, Spain^248^ | Case report | 1 | - 56-year-old female presented cough, fever and respiratory failure for 5 days after travel to Venice - Intubated for 10 days - 48-hours outside ICU, developed acute confusional syndrome - EEG and cranial MRI consistent with inflammatory encephalopathy related to SARS-CoV-2 - Radiological lesions may be secondary to inflammatory reaction, vs tissue injury due to viral invasion |
| New York City, New York^249^ | Case report | 1 | - 50-year-old female presented with 1-week history of dyspnea and cough; nasopharyngeal swab positive for SARS-CoV-2 - Intubated on hospital day 2 and brain CT on day 17 and brain MRI on hospital day 23, suggested delayed post-hypoxic leukoencephalopathy |
| New York City, USA^250^ | Case report | 1 | - 20-year-old female with SARS-CoV-2 presented with 4-day history of upper respiratory symptoms, fevers and sudden acute altered mental status diagnosed with encephalitis - An extensive work up led to the most likely cause for the neurologic decompensation to be viewed as SARS-CoV-2 symptomology. |
| Toulouse, France^251^ | Case report | 1 | - 51-year-old male hospitalized after 10 days of fever and cough; nasal swab positive for COVID-19 - MRI at day 22 revealed progressing lesions with diffuse hyperintense lesions in the thalami, cerebellum, brainstem, supratentorial grey and white matters; COVID-19 necrotising haemorrhagic encephalopathy - Resolved with steroids and polyvalent immunoglobulin |
| UAE^252^ | Case report | 1 | - 36-year-old male presented with 2 days history of fever, headache, body pain, cough, diarrhea and vomiting. The patient was negative for SARS-CoV-2 and diagnosed as gastroenteritis and discharged - 4-days later patient went back to hospital complaining of the same symptoms and additionally he presented with drowsiness and appeared mildly confused. - COVID-19 swab was repeated and resulted positive - Bilateral supratentorial leptomeningeal increased enhancement was detected and further supported the diagnosis of COVID-19-related meningoencephalitis. |
| United Kingdom^253^ | Case report | 1 | - 66-year-old female with no notable medical history presented with a few hours of history confusion - 6-hours after admission she had a single, spontaneously resolving, generalized tonic-clonic seizure and no respiratory symptoms - MRI on day 2 were consistent with limbic encephalitis - Nasopharyngeal swab was positive for SARS-CoV-2 |
| ***Uppsala, Sweden**^254^ | Case report | 1 | - 55-year-old female presented with fever and myalgia; nasopharyngeal swab positive for SARS-CoV-2 - MRI revealed low attenuating areas in the thalami and midbrain and acute necrotizing encephalitis - SARS-CoV-2 was positive in CSF 19 days after symptom onset, after testing negative twice |
| Varese, Italy^255^ | Case report | 1 | - 64-year-old female presented with 1-day history of fever and dyspnea - Nasopharyngeal swab tested positive for SARS-CoV-2 - After 24 hours, taken to ICU and sedated - Woke up on day 25 complaining of blurred vision - Brain imaging consistent with PRES |
| New York City, USA^199^ | Case report | 1 | - 64-year-old female presented with respiratory symptoms tested positive for COVID-19 and later developed PRES |
| Samsun, Turkey^256^ | Case report | 1 | - 35-year-old female presented with headache and seizures - Magnetic resonance imaging findings were suggestive of high-grade glioma - Neurologic symptoms resolved after a left anterior temporal lobectomy was performed - Histologic examination revealed encephalitis - Postoperatively, our patient tested positive for COVID-19 |
| Wuhan, China^257^ | Case report | 1 | - Frank meningoencephalitis: nuchal rigidity, Kernig’s and Brudzinski’s signs, extensor plantar response - SARS-CoV-2 was not detected in the CSF; symptoms resolved with supportive care |
| Yanagido, Japan^258^ | Case report | 1 | - 75-year-old male presented with left-dominant kinetic tremor in his hands, walking instability, and urinary incontinence - Brain MRI revealed abnormal hyperintensity in the splenium of corpus callosum, suggesting clinically mild encephalitis/encephalopathy with a reversible splenial lesion - Later throat swab RT-PCR tested positive for SARS-CoV-2 |
| Boston, USA^259^ | Case report | 1 | - Elderly male presented with worsening fatigue, headache, and new oxygen requirement 6-days after diagnosis of COVID-19 - 3-days later mutism, staring, posturing, grimacing, echolalia, verbigeration, stereotypy, rigidity, waxy flexibility, and automatic obedience suggested catatonia - Development of catatonic symptoms alongside peaking proinflammatory markers such as interleukin-6 and C-Reactive Protein |
| Madrid, Spain^260^ | Case report | 1 | - 58-year-old male developed an asymmetric hypokinetic-rigid syndrome alongside other complications such as hyposmia, generalized myoclonus, fluctuating and transient changes in level of consciousness, opsoclonus after severe SARS-CoV-2 infection - Spontaneous improvement without any specific therapy - Suggests the involvement of basal brain structures |
| New Haven, USA^261^ | Case report | 1 | - 78-year-old immunocompromised woman with altered mental status and seizure-like activity - Found to have SARS-CoV-2 infection but not in the CSF (although inflammatory markers were present) |
| ***Madrid, Spain**^262^ | Case report | 1 | - 74-year-old woman with gastrointestinal symptoms followed by headache and impaired consciousness - MRI showed small 3mm focus of restricted diffusion that was also hyperintense on FLAIR - Positive CSF RT-PCR for COVID-19 in the absence of inflammatory CSF |
| London, UK^263^ | Case report | 1 | - 55-year-old woman with post-COVID-19 delirium followed by florid psychotic symptoms consisting of persecutory delusion, complex visual and auditory hallucinations and Capgras phenomenon, for 40 days - Elevated tumour necrosis factor (TNF)-α. Normal MRI Brain, EEG, CSF, autoimmune panel |
| Geneva, Switzerland^264^ | Case report | 1 | - 56-year-old woman with COVID-19 and acute respiratory distress syndrome presented with altered mental status in the ICU - Video-EEG showed focal monomorphic theta slowing in bilateral frontal-central regions - MRI showed abundant microbleeds in bilateral white matter junction, corpus callosum and internal capsule, suggestive of Critical Illness-Associated Cerebral Microbleeds - CSF unremarkable and negative for SARS-CoV-2 |
| Marseille, France^265^ | Case report | 1 | - 63-year-old man with ARDS requiring mechanical ventilation and ECMO, followed by delirium - MRI showed callosal and juxtacortical hematomas associated with countless and punctate cerebral microbleeds disseminated in the corpus callosum and along the gray/white matter interface - Diagnosed as Critical Illness-associated Cerebral Microbleeds |
| Tehran, Iran^266^ | Case report | 1 | - 39-year-old female with typical COVID-19 symptoms who then developed a para-infectious encephalitis - Had self-limited seizures and decreased level of consciousness - MRI Brain showed FLAIR hyperintensities in bilateral thalami, medial temporal and pons with corresponding T1 hypointensities and no gadolinium enhancement - CSF negative for SARS-CoV-2, unremarkable otherwise |
| Connecticut, USA^267^ | Case report | 1 | - 50-year-old male found unresponsive at home; SARS-CoV-2 positive (throat swab RT-PCR) - Exam showed posturing with bilateral upper extremity extension on hyperpronation, normal reflexes - MRI showed multiple punctate foci of restricted diffusion in centrum semiovale and periventricular white matter (infarcts of embolic origin) - High procalcitonin, uptrending troponin, and progressive elevated creatinine; no improvement in 14-days |
| Philadelphia, USA^268^ | Case report | 1 | - 52-year-old woman with bilateral hand paresthesias followed by cough, dyspnea, headache, confusion - Refractory hypoxemia requiring mechanical ventilation within 1 hour of presentation - MRI showed bilateral globus pallidus lesions in keeping with hypoxic ischemic injury |
| Royal Oak, USA^269^ | Case report | 1 | - 66-year-old woman with lethargy, aphasia, right-sided weakness, in the context of severe COVID-19 - CT Head showed hypodense corpus callosum and left parietotemporal hypodensities - MRI showed diffusion restriction and microhemorrhages associated with these lesions - Attributed by the authors to a cytokine storm |
| Rotterdam, Netherlands^270^ | Case report | 1 | - 57-year-old male with critical respiratory distress from COVID-19 bilateral pneumonia - Developed lethal cerebral edema after 2-weeks in the ICU despite improving respiration |
| Boston, USA^271^ | Case report | 1 | - 47-year-old man with COVID-19 and prolonged coma, progressing from vegetative state to minimally conscious state minus and plus - MRI showed basal ganglia and thalamic T2 hyperintensities - Resting-state fMRI during coma showed intact functional network connectivity, and weeks later he recovered the ability to follow commands |
| Singapore^272^ | Case report | 1 | - 61-year-old man with COVID-19 and hypoxic respiratory failure and cytokine release syndrome with shock, acute kidney injury requiring renal replacement therapy, and hepatic dysfunction, on remdesivir - Markedly elevated inflammatory markers, severely encephalopathic with flaccid tetraplegia - CT/MRI showed finds of acute hemorrhagic leukoencephalitis |
| London, UK^273^ | Case report | 1 | - 41-year-old man with acute behavioural disruption and 10-day history of dry cough and fever - Sexually disinhibited, pressured speech, grandiose ideas in keeping with mania - SARS-CoV-2 positive in swab but not in CSF |
| Grenoble, France^274^ | Case report | 1 | - 96-year-old woman: bilateral tonic-clonic seizures, fever, then left hemiparesis; no respiratory symptoms - Had developed anosmia, dysgeusia, and behavioral dysexecutive syndrome two days before - Nasopharyngeal SARS-CoV-2 RT-PCR initially negative but CT Chest suggestive - CSF showed 8 leukocytes/mm^3^ (75% polymorphonuclear neutrophilic cells), negative for SARS-CoV-2 - Brain MRI showed hyperintensity of olfactory tracts on T2 and diffusion-weighted imaging - Ten days after admission, SARS-CoV-2 serology returned positive |
| Toronto, Canada^275^ | Case report | 1 | - 18-year-old woman presented with 1-week of fever, fatigue, malaise, loss of appetite, then drowsiness and confusion, followed by urinary retention and a generalized tonic-clonic seizure the next day - On exam, macular rash seen, and patient was drowsy and disoriented, unable to walk or stand - Labs showed thrombocytopenia, nasopharyngeal RT-PCR positive for SARS-CoV-2 - CT/MRI Brain were normal, but EEG showed moderated intermittent theta/delta slow-wave activity predominantly over the frontocentrotemporal regions - CSF showed increased white cells but normal protein, and was negative for SARS-CoV-2 - Clinical picture evolved to include myoclonic jerks, mood/behavioural changes, pruritis, chest pain - Second admission 2-weeks later with right/bilateral jerks, MRI: hyperintensities in claustrum bilaterally - Treated with 5-day course of IV methylprednisolone for “antibody-negative” autoimmune encephalitis, followed by weekly pulse steroids as an outpatient - MRI 1-month later showed resolution, jerks and seizures resolved but memory deficits persisted |
| **CNS Demyelination** | | | |
| Rochester, USA^276^ | Case report | 1 | - 71-year-old man initially presented with fatigue and exertional dyspnea while respiratory status deteriorated steadily with worsening symptoms - Swab tested positive for SARS CoV-2 - Post-mortem examinations of the brain indicated ADEM-like lesions |
| ***Genova, Italy**^277^ | Case report | 1 | - 64-year-old female presented with previous influenza-like syndrome and both anosmia and ageusia. Patient developed bilateral vision impairment associated with sensory deficit on right leg - MRI consistent with ADEM; PCR for SARS-CoV-2 negative on nasal swab but positive in CSF sample - Findings indicative of immune-mediated CNS disease that occurred after SARS-CoV-2 infection |
| Tehran, Iran^278^ | Case report | 1 | - 58-year-old male presented with decreased level of consciousness and the inability to walk - Nasopharyngeal swab was positive for SARS-CoV-2, imaging consistent with ADEM - Findings suggested that COVID-19 infection might have led to the development of ADEM |
| Marseille, France^279^ | Case report | 1 | - 54-year-old woman with typical COVID-19 symptoms followed by altered mental status - CSF unremarkable, SARS-CoV-2 negative, slowing on EEG - MRI showed bilateral but asymmetrical lesions with periventricular, globus pallidus, deep white matter involvement and contrast enhancement, raising the question of acute demyelination (normal vessels) |
| Connecticut, USA^280^ | Case report | 1 | - 51-year-old female with COVID-19 developed coma and impaired oculocephalic response to one side - MRI Brain showed acute multifocal demyelinating lesions, CSF negative for infection - After high-dose steroids and IVIG, regained consciousness over several weeks. - Final diagnosis was acute disseminated encephalomyelitis (ADEM) due to COVID-19 |
| Tehran, Iran^281^ | Case report | 1 | - 21-year-old male with encephalomyelitis following GI and respiratory infectious symptoms - Two CSF samples showed mononuclear pleocytosis, elevated protein, and hypoglycorrhachia - MRI Brain showed bilateral posterior internal capsule lesions extending to the ventral portion of the pons and a marbled splenium hyperintensity pattern - Cervical and thoracic MRI showed longitudinally extensive transverse myelitis, negative AQP4 and MOG antibodies - Chest CT and IgG testing in keeping with COVID-19 but oropharyngeal swabs negative – authors diagnosed a post-COVID-19 demyelinating event in the CNS |
| Brescia, Italy^282^ | Case report | 1 | - 54-year-old female with COVID-19 interstitial pneumonia and seizures - MRI showed demyelinating-type lesions in the brain and C-spine - CSF unremarkable and negative for SARS-CoV-2 - High-dose steroid treatment was followed by neurological and respiratory recovery |
| **Myelitis (including Necrotizing and Transverse Myelitis)** | | | |
| Central Connecticut, USA^280^ | Case report | 1 | - 51-year-old woman presented with dyspnea, fever, and vomiting with no pertinent neurological history - Tested positive for SARS-CoV-2 from nasopharyngeal swab - Developed coma and impaired oculocephalic response to one side |
| Dubai, UAE^283^ | Case report | 1 | - 32-year-old male presented with previous high-grade fever and flu-like symptoms, as well as sudden bilateral lower limb weakness - Findings were suggestive of acute myelitis likely as direct damage or a sequalae of a post infectious process of COVID-19 |
| Esslingen, Germany^284^ | Case report | 1 | - 60-year-old patient admitted with typical respiratory symptoms of COVID-19, no neurological symptoms. - Tested positive for SARS-CoV-2 - Developed multifocal transverse myelitis shortly after COVID-19 infection |
| Los Angeles, USA^285^ | Case report | 1 | - 26-year-old male presented with vision loss first affecting the left eye, then right eye 3 days later - Testing from nasal and oropharyngeal swabs returned positive for SARS-CoV-2 - Findings suggested bilateral severe optic neuritis and myelitis were triggered by COVID-19 infection |
| Michigan, USA^286^ | Case report | 1 | - 61-year-old female presented with progressive weakness - SARS-CoV-2 was positive in the nasopharyngeal swab but negative in the CSF - Patient developed acute necrotizing myelitis and acute motor axonal neuropathy at the same time |
| New York City, USA^287^ | Case report | 1 | - 44-year-old man initially presenting with urinary retention for 2 days with additional symptoms of bilateral lower extremity weakness and numbness, and an inability to walk - SARS-CoV-2 tested positive, CSF unremarkable - Imaging findings suggested ADEM |
| Rennaz, Switzerland ^288^ | Case report | 1 | - 63-year old male had a viral prodrome for 7 days followed by progressive weakness in lower limbs - The pathogenic virus was deemed to likely have been SARS-CoV-2, although tests were negative - Findings suggest transverse myelitis appeared 2 weeks after the mild viral symptoms presumably caused by COVID-19 infection |
| Sydney, Australia^289^ | Case report | 1 | - 60-year-old male presented with bilateral lower limb weakness, urinary retention and constipation - Diagnosed with COVID-19 10-days prior - Neurological symptoms and recent viral infection suggested post-viral acute transverse myelitis |
| Varese, Italy^290^ | Case report | 1 | - 22-year-old female presented with worsening dyspnea, fever and loss of consciousness - Bilateral ground-glass opacity involving lung parenchyma suggested COVID-19 and RT-PCR resulted positive for SARS-CoV-2 - On 15^th^ day, developed acute flaccid tetraparesis, more pronounced in distal muscles, with hyperreflexia, fecal and urinary incontinence - Brain and spine MRI only showed subacute small frontal hemorrhage - RT-PCR CSF sample was negative for SARS-CoV-2 infection |
| Terrassa, Spain^291^ | Case report | 1 | - 69-year-old female with cervical pain, imbalance, motor weakness, and prior fever and dry cough - SARS-CoV-2 PCR was positive in nasopharyngeal swab and negative in CSF - Findings led to the diagnosis of acute necrotizing myelitis alongside the COVID-19 infection |
| ***São Paulo, Brazil**^292^ | Case report | 1 | - 42-year-old patient presented with hypoesthesia and 3-week history of mild respiratory symptoms including coryza and nasal obstruction without fever - Cervical spine MRI showed small left lateral ventral lesion without mass effect, without enhancement - RT-PCR nasal and pharyngeal samples for SARS-CoV-2 was negative - RT-PCR CSF examination 16-days later was positive for SARS-CoV-2 |
| **Seizures** | | | |
| Boston, USA^293^ | Retrospective case series | 7 | - Patients ranged from 37 to 88-years-old - 3 patients had history of well-controlled epilepsy - 4 patients had new onset seizures, including 2 patients with prior history of remote stroke - 3 patients had no prior symptoms of COVID-19 - In all patients seizures prompted presentation to emergency department |
| New York, USA^294^ | Case series | 2 | - Two advanced-age, non-epileptic, male patients with history of lung disease presented with concern for infection and tested positive for SARS-CoV-2 - Developed acute encephalopathy days into hospitalization with clinical and electrographic seizures - Resolution of seizures achieved with levetiracetam |
| Birmingham, USA^295^ | Case series | 2 | - De novo status epilepticus in two African‐American women with laboratory‐confirmed COVID-19 - Seizures were the initial presentation in one, the other had fluctuating mental status and pneumonia |
| Dublin, Ireland^296^ | Case report | 1 | - 20-year-old previously healthy young male brought to hospital following seizure with a 3-day history complaining of myalgia, lethargy and fever - Nasopharyngeal swab for SARS-CoV-2 was negative, with a second swab 2-days later testing positive |
| Italy^297^ | Case report | 1 | - 54-year-old firefighter with no neurological history admitted after a single seizure characterized by clonic movements in the right arm and loss of consciousness - Reported suffering from conjunctivitis in the last 10 days and a mild fever in the past week - Nasopharyngeal swabs tested positive for SARS-CoV-2 - Seizure as a possible symptom of SARS-CoV-2 |
| Japan^298^ | Case report | 1 | - 44-year-old male presented with intermittent twitch on the left hand and face with a history 6-months prior he has epilepsy following cerebral venous thrombosis - Anosmia and glass-like shadows suggested COVID-19 and RT-PCR tested positive for SARS-CoV-2 |
| Kempen, Germany^299^ | Case report | 1 | - 70-year-old female admitted due to recurrent non-epileptic seizures/convulsive syncopes - Fever, cough, as ore through, loss of gustation or olfaction were not reported, and vital signs and clinical examination did not show abnormalities - That evening patient exhibited signs of dyspnea; oropharyngeal swabs were positive for SARS-CoV-2 - Seizure as a possible initial symptom of SARS-CoV-2 |
| United States^300^ | Case report | 1 | - 72-year-old male with history of hypertension, coronary artery disease with stent, diabetes type 2 and stage kidney disease presented with complaints of weakness after experiencing hypoglycemic episode - Abnormal labs suggested COVID-19 and the RT-PCR was SARS-CoV-2 positive - 24 hour EEG showed six left temporal seizures |
| Nantes, France^301^ | Case report | 1 | - 59-year-old male with non-lesional status epilepticus, confirmed COVID-19 - MRI and CSF unremarkable, CSF negative for SARS-CoV-2 – improved with clobazam/levetiracetam |
| Madrid, Spain^302^ | Case report | 1 | - 74-year-old male with IgG kappa multiple myeloma, severe bilateral COVID-19 pneumonia - Developed refractory status epilepticus with MRI showing posterior reversible encephalopathy syndrome |
| Paris, France^303^ | Case report | 1 | - 69-year-old male admitted for status epilepticus preceded by anosmia and typical COVID-19 symptoms - RT-PCR positive (tracheal) for SARS-CoV-2, CSF unremarkable and negative for SARS-CoV-2 - EEG & MRI showed right frontal lobe involvement (hyperintense on MRI, lateralized discharges on EEG) |
| **Movement disorders** | | | |
| Paris, France^304^ | Case series | 5 | - All patients underwent intubation and mechanical ventilation - Abnormal movements developed 23 ± 7 days after ICU discharge - 4 patients had upper limbs postural and action-tremor, 1 patient also had irregular orthostatic tremor - 1 patient had bilateral upper limbs jerky/myoclonic abnormal movements - Overall EEG recordings supported a mixed cortical-subcortical pattern of myoclonic jerks |
| Madrid, Spain^305^ | Case series | 3 | - Three patients (2 males, 1 female) from 63 to 88-year-old presented with hypersomnia and generalized myoclonus following onset of COVID-19 - Myoclonus generalized with both positive and negative jerks - Recovery, at least partially, with immunotherapy |
| *** Shiraz, Iran**^306^ | Case report | 1 | - 47-year-old male with progressive vertigo and ataxia for 7 days and cerebellar findings on exam - MRI Brain showed cerebellar edema with leptomeningeal enhancement - CSF showed mild lymphocytic pleocytosis, elevated protein, and lactate dehydrogenase - **SARS-CoV-2 RNA was detected in the oropharyngeal/nasopharyngeal and CSF specimens** - Patient improved while on treatment with lopinavir/ritonavir |
| Ahmedabad, India^307^ | Case report | 1 | - Middle-aged patient with imbalance and involuntary jerky movements of the body, 3-weeks after initial recovery from COVID-19 lung infection (positive CT thorax and RT-PCR nasal swab) - Opsoclonus, cortical myoclonus and cerebellar ataxia on exam - Unremarkable MRI Brain, CSF studies, and antibody studies - Recovered after treatment with IV methylprednisolone, sodium valproate, clonazepam, levetiracetam |

**Supplementary Table 7. Key evidence of PNS manifestations of COVID-19 from cohort studies and large case series (>10 patients).** Studies are listed in descending order of sample size.

| **Location** | **Study Type** | **Number of patients** | **Key findings** |
| --- | --- | --- | --- |
| **Guillain-Barré syndrome (GBS)** | | | |
| Multi-national (but primarily USA)^97^ | Retrospective cohort study | 236,379 | - Analysis of TriNetX database including matched controls with influenza and other respiratory infections (n=236,038 for controls) – see Table 4 for more details - Estimated 6-month incidence of GBS: 0.08% (95%CI 0.06-0.11); of any nerve, nerve root, or plexus disorder: 2.85% (2.69-3.03); of myoneural junction or muscle disease: 0.45% (0.40-0.52) - In subgroup with intensive care admission, incidence of GBS: 0.33% (0.21-0.54); of any nerve, nerve root, or plexus disorder: 4.24% (3.58-5.03); myoneural junction or muscle disease: 3.35% (2.76-4.05) - These disorders were more common in patients who had COVID-19 than in those who had influenza (e.g. GBS – HR 1.21, 95% CI 0.72–2.04) and those who had other respiratory tract infections (2.06, 1.43–2.96). HRs were higher in patients who had more severe COVID-19 |
| Spain (61 centres)^308^ | Retrospective, case–control, ED‐based, multicenter study | 71,904 with COVID-19 | - 11 GBS cases identified among 71,904 COVID patients seen over 2 months at 61 emergency departments (EDs) during the peak of the pandemic’s first wave in Spain - The relative frequency of GBS was higher in COVID (0.15%) than non‐COVID (0.02%) patients (OR: 6.30, 95%CI 3.18–12.5), as was standardized incidence (9.44 and 0.69 cases/100,000 inhabitant‐years, respectively, OR: 13.5, 95% CI 9.87–18.4) - Olfactory–gustatory disorders were more frequent in COVID‐GBS than non‐COVID–GBS and COVID–non‐GBS patients - Although COVID‐GBS patients were more frequently admitted to intensive care, mortality was not increased vs non-COVID controls |
| United Kingdom^309^ | National registry study (with two comparative periods) | 25 with probable or definite COVID-19 | - Epidemiology of GBS cases reported to the UK National Immunoglobulin Database was studied from 2016-2019 and compared to cases reported during the COVID-19 pandemic - UK population data for COVID-19 infection collated from UK public health bodies - Members of the British Peripheral Nerve Society also prospectively reported incident cases of GBS during the pandemic at their hospitals to a central register - Incidence of GBS 2016-2019 was 1.65-1.88/100,000 individuals/year, and fell March-May 2020 compared to the same months of 2016-2019 - GBS & COVID-19 incidences during pandemic varied among regions, did not correlate with each other - 47 GBS cases were reported to the central register (COVID-19 status: 13 definite, 12 probable, 22 non-COVID-19) - No significant differences in pattern of weakness, time to nadir, neurophysiology, CSF findings or outcome between these groups, but intubation more frequent in COVID-19 group (54% vs 23%) - Concluded there were no epidemiological or phenotypic clues of SARS-CoV-2 being causative of GBS |
| Northern Italy^310^ | Retrospective case-control study | 30 with COVID-19 | - GBS cases (n=34) diagnosed in 12 referral hospitals from Lombardy and Veneto (which had the highest number of COVID-19 patients in Northern Italy) in March-April 2020 were compared to control population of GBS cases (n=13) diagnosed in March-April 2019 at the same hospitals - 30 of 34 (88.2%) patients with GBS in 2020 had COVID-19 - Incidence in 2020 was 0.202/100,000/month vs 0.077/100,000/month in 2019 – a 2.6-fold increase - Estimated incidence of GBS in COVID-19-positive patients was 47.9/100,000 overall and 236/100,000 if hospitalized - COVID-GBS patients, compared with non-COVID-GBS patients, showed lower MRC sum score, higher frequency of demyelinating subtype, more frequent hypotension, and higher rate of admission to intensive care unit |

**Supplementary Table 8. Relevant case reports or brief series (≤10 patients) on PNS manifestations of COVID-19**

| **Location** | **Number of patients** | **Key findings** |
| --- | --- | --- |
| **Guillain-Barré Syndrome (GBS)** | | |
| Besançon, France^311^ | 6 | - Reported unusually high number of GBS cases, admitting 7 patients while in previous 3 years, in same time period and region, usually admit 0 to 2 cases - SARS-CoV-2 nasopharyngeal swab and serology were negative in all six patients |
| Italy^312^ | 5 | - Five COVID-19 patients ranging from 49 to 94-years-old developed neurological symptoms, mainly expressing as polyradiculoneuritis and cranial polyneuritis and diagnosed with GBS based on clinical presentation, CSF analysis, and nerve conduction studies (NCS) - PCR for SARS-CoV-2 in CSF tested in four out of five patients, with all negative tests |
| Switzerland^313^ | 3 | - Three patients presented demyelinating electrophysiological pattern - Neurological symptoms appeared within the first 22 days (7, 15, and 22 days) after appearance of typical COVID-19-related symptoms - SARS-CoV-2 of the CSF was tested in two out of three patients with negative results and positive nasopharyngeal swab; third case showed SARS-CoV-2 seroconversion in the serum and the fourth nasopharyngeal swab was positive |
| Kashan, Iran^314^ | 2 | - 46-year-old male and 65-year-old male with progressive ascending weakness several days after COVID-19 - NCS and CSF were in keeping with GBS - Second patient received IVIG (first one was already ambulatory without treatment |
| Italy ^315^ | 2 | - 55-year-old man hospitalized for severe respiratory syndrome tested positive for COVID-19, after 20 days of admission to ICU, patient showed acute onset of bilateral eyelid ptosis, dysphagia and dysphonia. NCS revealed symmetric demyelinating findings; administered intravenous immunoglobulins (IVIG) with very rapid clinical response on swallowing, speech, tongue motility and strength, as well as eyelid ptosis - 60-year-old man hospitalized for fever and cough, positive for COVID-19. 20 days later, he presented with acute weakness in lower limbs with distal distribution and right foot drop. NCS showed Acute Motor & Sensory Axonal Neuropathy. Improvement seen after 5-days of IVIG. - No SARS-CoV-2 detected in CSF in either case |
| New York City, USA^316^ | 2 | - 36-year-old man with ophthalmoparesis, leg paresthesia, and areflexia suggesting GBS, with MRI showing enhancement and enlargement of the left oculomotor nerve; improved with 3 days of IVIG - 71-year-old woman with painless diplopia, optic nerve sheath enhancement on MRI; lumbar puncture was normal with normal opening pressure |
| USA^317^ | 2 | - Two patients, a 48-year-old female and 75-year-old-male, presented with COVID-19 symptoms such as cough, shortness of breath and fever - Developed neurological symptoms and were diagnosed with Bell’s palsy and GBS respectively |
| Qazvin, Iran^318^ | 2 | - 38-year-old male presented with 5-day history of ascending paresthesia and bilateral facial droop and his 14-year-old daughter presented with 2-day history of progressive ascending quadriparesthesia with mild lower limb weakness - Neurological examination and electromyography-nerve conduction study findings were consisted with GBS; throat swab sample was positive for COVID-19 - Example of familial occurrence of GBS after COVID-19 infection |
| Bursa, Turkey^319^ | 1 | - 53-year-old female presented with 3-day history of dysarthria associated with progressive weakness and numbness of lower extremities, could only walk with assistance, slight weakness in her hand muscles; GBS was considered most likely diagnosis - Nasopharyngeal swab for SARS-CoV-2 was positive - CSF test for SARS-CoV-2 was negative - Improved neurologic findings two weeks after onset of symptoms - Speculate pathophysiologic mechanism of GBS in COVID-19 to be para-infectious rather than post-infectious |
| Geneva, Switzerland^313^ | 1 | - Man in his seventies was hospitalized because of paraparesis, distal allodynia, difficulties in voiding and constipation; ten days prior he developed myalgia, fatigue and a dry cough - Nasopharyngeal swab tested positive for SARS-CoV-2 prior to first signs of polyneuropathy - Neurological findings suggested acute inflammatory demyelinating polyneuropathy |
| Innsbruck, Austria^320^ | 1 | - 68-year-old male developed dry cough, headache, fatigue, myalgia and fever followed by anosmia and ageusia, tested negative for SARS-CoV-2 RNA - Patient recovered from respiratory symptoms, but still complained of severe fatigue and developed symmetric distal tingling in both feet - GBS diagnosed based on neurological presentation; administered IVIG - Oropharyngeal swabs repeatedly negative but anti-SARS-CoV-2 antibodies highly positive in serum and CSF |
| Iran^321^ | 1 | - 41-year-old male developed ascending paresthesia and paralysis after SARS-CoV-2 infection - Electrodiagnostic evaluation revealed demyelinating type polyneuropathy, suggesting GBS - CSF was negative for SARS-CoV-2 |
| Italy^322^ | 1 | - 71-year-old male patient presented with a 3-day history of subacute onset of paresthesia at limb extremities, followed by distal weakness rapidly evolving to a severe, flaccid tetraparesis - 1-week prior he had a low grade fever for a few days - Neurological findings interpreted as severe form of acute polyradiculoneuritis with prominent demyelinating features, suggesting GBS - Administered high-dose IVIG, severe respiratory failure developed in first 24 hours |
| Italy^323^ | 1 | - 70-year-old female presented with asthenia, hands/feet paresthesia and gait difficulties progressing over 1 day - 24-days prior she had developed a fever and dry cough and 1-day later tested positive for COVID-19, symptoms of which resolved in a few days - Neurophysiologic findings were consistent with a diagnosis of GBS - Administered 5-days IVIG however did not improve symptoms and patient was intubated due to respiratory failure due to worsening of muscle weakness |
| Italy^324^ | 1 | - 68-year-old male presented with acute progressive symmetric ascending flaccid tetraparesis - Oropharyngeal swab for SARS-CoV-2 tested positive - Neurological examination, CSF assessment and electrophysiological studies suggested Acute Inflammatory Demyelinating Polyradiculoneuropathy, subtype of GBS |
| Italy^325^ | 1 | - 66-year-old woman presented with a 3-day history of increasing difficulty walking, acute fatigue, and 10-days earlier a mild fever and cough that spontaneously resolved - Rhinopharyngeal swab for SARS-CoV-2 was negative - She was paraparetic with a rapidly progressive symmetric weakness in lower limbs, initial distal weakness in upper limbs, and diffuse areflexia, suggesting GBS - SARS-CoV-2 was not detected in CSF - Progression of neurological symptoms and respiratory symptoms progressed simultaneously, resembling para-infectious manifestation |
| Italy^326^ | 1 | - Man in 60s presented with 3-day history of progressive limb weakness and distal paresthesia - 20-days prior, he had developed fever, headache and myalgia, followed by anosmia and ageusia - Polymerase-chain reaction for SARS-CoV-2 on CSF tested negative but SARS-CoV-2 IgG tested positive - Treated with 5-day course of IVIG |
| Jingzhou, China^327^ | 1 | - 61-year-old woman presented with GBS eight days before developing typical COVID-19 symptoms - GBS may have been her initial symptom of GBS - CSF showed albuminocytologic dissociation (high protein with white cells <5/mm^3^) typical of GBS |
| [London, UK](https://www.ncbi.nlm.nih.gov/pmc/articles/PMC7298664/)^328^ | 1 | - 57-year-old male with a progressive flaccid symmetrical motor and sensory neuropathy following 1-week history of cough and malaise - Diagnosed with GBS secondary to COVID-19, suggested post-infectious complication - Administered IVIG, proceeding to have worsening respiratory function |
| Madrid, Spain^329^ | 1 | - 61-year-old patient had fever and coughing without dyspnea on day 1 of illness - Nasopharyngeal sampling RT-PCR indicated SARS-CoV-2 - On day 10, noted liquid dripping on his right facial commissure - Rare variant of GBS in COVID-19 infection |
| Monza,  [Italy](https://nn.neurology.org/content/7/4/e741.abstract)^330^ | 1 | - 71-year-old male patient presented with a 3-day history of subacute onset of paresthesia at limb extremities, followed by distal weakness rapidly evolving to severe - He had a low-grade fever for a few days the week prior - Nasopharyngeal swab tested positive for SARS-CoV-2 while CSF was negative |
| Morocco^331^ | 1 | - 70-year-old female receiving prednisone as maintenance therapy for rheumatoid arthritis, presented with a rapidly, bilateral weakness and tingling sensation in all four extremities; 3-days prior to the symptom’s onset the patient presented with an episode of dry cough spontaneously resolving within 48 hours - Neurological findings were consistent with AMSAN, a subtype of GBS - Oropharyngeal swab was positive for SARS-CoV-2 while CSF was negative |
| New York, USA^332^ | 1 | - 67-year-old female with rapidly progressive quadriparesis, low back pain, paresthesias, urinary retention - 10-days prior developed a cough, nausea, and diminished appetite - Nasopharyngeal SARS-CoV-2 RT-PCR test was positive - Motor dysfunctions continued to progress - CSF was negative for SARS-CoV-2 |
| Pittsburgh, USA^333^ | 1 | - 72-year-old male presented with symmetric paresthesias and ascending appendicular weakness - 7-days prior developed diarrhea, anorexia, and chills, which resolved in 5 days - Nasopharyngeal PCR SARS-CoV-2 was positive - Prominent dysautonomia - Microbiological CSF testing was negative, including for SARS-CoV-2 |
| Pittsburgh, USA^334^ | 1 | - 54-year-old male patient with ascending limb weakness and numbness followed respiratory symptoms - 2-weeks prior developed rhinorrhea, odynophagia, fevers, chills, night sweats; wife was positive for COVID-19 - NCS consistent with GBS; administered 5-days of IVIG |
| Saint-Étienne, France^335^ | 1 | - 64-year-old male admitted to hospital after tear of rotator cuff, presented with fever and cough for 2-days, nasopharyngeal swab tested positive for SARS-CoV-2 - 11-days after COVID-19 symptom onset, patient complained of paresthesia in feet and hands - NCS 5-days after neurological symptom onset showed demyelinating pattern in accordance with GBS - GBS as a complication of COVID-19 |
| Sari, Iran^336^ | 1 | - 65-year-old man with electrophysiological features in keeping with the acute motor and sensory axonal neuropathy (AMSAN) variant of GBS - CSF was not obtained |
| Selters, Germany^337^ | 1 | - 54-year-old female patient presented with acute, proximal, moderate, symmetric paraparesis - Symptoms began 3 weeks after a positive oropharyngeal test for COVID-19 - Reported loss of smell and taste 2 weeks prior to GBS symptoms but no respiratory symptoms - SARS-CoV-2 was not tested in CSF |
| Suhl, Germany^338^ | 1 | - 56-year-old female with dry cough, mild fever and general weakness; positive for SARS-CoV-2 - 7-days after positive test, presented with weakness of her limbs and tingling sensation in all fingertips and toes - Showed typical, but severe, course of GBS - SARS-CoV-2 RT-PCR in CSF was negative |
| Netherlands^339^ | 1 | - 50-year-old male presented with 4 days of progressive bilateral facial weakness, paresthesia of distal extremities and an unsteady gait - 4 weeks prior he had an episode of dry cough lasting several days without fever or other symptoms of infection - PCR for SARS-CoV-2 in the CSF was negative - Fecal PCR reaction and serum immunoglobulin IgM and IgG for SARS-CoV-2 were positive |
| [United Kingdom](https://www.ncbi.nlm.nih.gov/pmc/articles/PMC7348325/)^340^ | 1 | - 49-year-old male presented with difficulty in mobilising and reported a 3-week history of shortness of breath, headache and cough; oropharyngeal swabs positive for SARS-CoV-2 - Patient returned 3-days later with worsening lower limb paraesthesia and unable to mobilize due to ascending lower limb weakness - NCS showed demyelinating polyneuropathy - Administered IVIG and his symptoms stabilized - Developed GBS as likely post-infectious complication 3-weeks after initial onset of symptoms |
| [USA](https://www.mayoclinicproceedings.org/article/S0025-6196(20)30545-0/fulltext#%20)^341^ | 1 | - 58-year-old female with travel history to Florida presented with progressive paraparesis and evolving areflexia suggesting GBS - SARS-CoV-2 nasopharyngeal swab, performed 3 weeks after onset of symptoms, was negative - Improved motor and gait examination after 5 sessions of every-other-day plasma exchange |
| USA ^342^ | 1 | - 21-year-old male presented with 4-day progressive history of fever, cough, dyspnea, diarrhea, nausea, headache, and sinonasal congestion - Bifacial weakness with paresthesia, GBS subtype - Administered treatment with 5 cycles of plasma exchange - CSF positive for IgG and IgM |
| Zaragoza, Spain^343^ | 1 | - 56-year-old female presented with recent unsteadiness and paraesthesia in both hands - 15 days prior, she complained of fever, dry cough and shortness of breath - PCR was positive for SARS-CoV-2 - Microbiological studies on CSF, including SARS-CoV-2, were negative - Administered IVIG, with significant improvement noted over the next 2 weeks |
| Imola, Italy^344^ | 1 | - 66-year-old Maroccan woman presented with weakness in all limbs, severe in lower, distal > proximal, with distal tingling sensation and pronounced lumbar pain for 8 days; absent reflexes - NCS consistent with AIDP. CSF showed albuminocytological dissociation - Nasopharyngeal swab RT-PCR positive for SARS-CoV-2; CSF not tested for SARS-CoV-2 - Improved with IVIG course |
| Ingolstadt, Germany^345^ | 1 | - 51-year-old male presented with 2-days of progressive upper and lower limb weakness, acral paresthesias preceded by fluctuating fever and flu-like symptoms with fatigue and dry cough - Weakness progressed to a locked-in syndrome needing mechanical ventilation - CSF showed albuminocytological dissociation, MRI showed generalized nerve root contrast enhancement - IVIG and plasma exchange treatment was followed by some motor improvement |
| Kyushu, Japan^346^ | 1 | - 69-year-old male with severe COVID-19 pneumonia requiring intubation - No cough reflex observed, tendon reflexes diminished – GBS diagnosed and given IVIG - Cough reflex recovered, patient successfully extubated five days after IVIG |
| Calgary, Canada^347^ | 1 | - 58-year-old male with acute-onset facial diplegia, dysarthria, and paresthesia in his feet - Labs showed persistent thrombocytosis, elevated D-Dimer; oropharyngeal RT-PCR positive for SARS-CoV-2 - MRI Brain showed bilateral intra- and extra-cranial facial nerve enhancement - CSF showed albuminocytologic dissociation, but was negative for SARS-CoV-2 - NCS consistent with GBS, treated with IVIG 5-day course, discharged with some improved facial movements |
| **Miller Fisher Syndrome (MFS) and similar presentations** | | |
| Northern Italy^348^ | 5 | - First neurological symptoms began 5-10 days after typical COVID-19 symptoms: lower-limb weakness/paresthesia in 4 patients, facial diplegia then ataxia and paresthesia in one patient - Flaccid quadriparesis evolved over 36-96 hours in 4 patients; three received mechanical ventilation - 2 patients had demyelinating features on electrophysiology, 3 had more axonal features - CSF showed albuminocytologic dissociation in three patients and normal protein in two patients - Antiganglioside antibodies were absent in 3 tested patients - MRI showed nerve root enhancement in 2 patients, facial nerve enhancement in one - All were treated with IVIG, two received a second course and one also receiving plasma exchange - At 4 weeks, two remained in ICU with mechanical ventilation, two were receiving physical therapy, and one was discharged and ambulatory |
| Madrid, Spain^349^ | 2 | - Patients presented with ophthalmoplegia, ataxia, and areflexia clinically in keeping with MFS, along with multiple cranial neuropathies and typical COVID-19 symptoms - CSF showed albuminocytologic dissociation - One patient tested positive for GD1b-IgG antibodies (versus GQ1b antibody more typical of MFS) - One patient treated with IVIG; both had full recovery except anosmia/ageusia in treated patient |
| Málaga, Spain^350^ | 1 | - 51-year-old female diagnosed with MFS 2-weeks after COVID-19 - RT-PCR SARS-CoV-2 negative; IgG was positive |
| Trieste, Italy^351^ | 1 | - 50-year-old female developed SARS-CoV-2 pneumonia - Developed neurological symptoms 10-days after admission in keeping with MFS - Administered IVIG; recovered after 14-days |
| Poole, United Kingdom^352^ | 1 | - 63-year-old male presented with fever; COVID-19 swab results positive - Neurological signs included complex opthalmoplegia, ataxia, and areflexia, suggesting MFS |
| **Other Cranial or Peripheral Neuropathies** | | |
| Pavia, Italy^353^ | 4 | - Patients presenting with fever, hyposmia, hypogeusia, cough; respiratory failure within 2 weeks - On weaning from respiratory support and post-tracheostomy, they had hoarseness, dysphagia, and tongue deviation. Normal Brain MRI, antibody assays - 20 weeks after respiratory symptoms, unilateral tongue deviation, and weakness of soft palate, trapezius, and sternocleidomastoid muscles persisted (asymmetric bulbar palsy) - EMG showed asymmetric changes of acute and/or chronic neurogenic changes in bulbar muscles without worsening over time, suggesting motor neuronopathy of medullary nuclei or lower cranial polyneuropathy |
| Besançon, France^354^ | 1 | - 62-year-old male with cranial nerve X and XII deficits post-intubation in keeping with Tapia syndrome - This presentation could have been solely explained by complications of intubation rather than COVID-19 (in particular, prolonged prone-position ventilation with lateral flexion of the head) |
| Auckland, New Zealand^355^ | 1 | - 55-year-old female presented with 7-day history of cough, fever, dyspnea; SARS-CoV-2 PCR positive - Several bilateral weakness of shoulder abduction and external rotation, mild weakness of finger abduction, and numbness over the distribution of the axillary nerves - Electromyography showed complete denervation of deltoid muscles bilaterally and of right infraspinatus and suggested bilateral suprascapular, axillary and ulnar neuropathies |
| Northern Ireland, United Kingdom^356^ | 1 | - 69-year-old male presented with 3-day history of bilateral lower limb weakness; tested positive for COVID-19 - Reduced power of 4/5 knee extension bilaterally; knee and ankle jerks were absent bilaterally - Received MRI of head and spin with no evidence of inflammation of demyelination - Peripheral neuropathy manifested before onset of typical flu-like symptoms of COVID-19 |
| Ehime Prefecture, Japan^357^ | 1 | - 35-year-old woman with COVID-19 pneumonia developed lower-motor-neuron pattern facial nerve palsy and olfactory dysfunction; CSF was unremarkable with negative SARS-CoV-2 PCR - Treated with traditional Japanese medicine Maoto |
| Nagoya, Japan^358^ | 1 | - 70-year-old male with oropharyngeal dysphagia, absent gag reflex, and consequent aspiration pneumonia during recovery from severe COVID-19 - Suggests that glossopharyngeal and vagal neuropathy may cause dysphagia after COVID-19 |
| Dresden, Germany^359^ | 1 | - 52-year-old man with mononeuropathic brachial neuritis on dominant side following infection with SARS‐CoV‐2 - CSF unremarkable, no evidence of SARS-CoV-2 infection or immunoglobulins - Partial pain relief with oral prednisolone course |
| Singapore^360^ | 1 | - 27-year-old man developed left facial nerve palsy on day 6 of COVID-19 illness beginning with cough, fever - CSF unremarkable, negative for typical viruses as well as SARS-CoV-2 IV - MRI Brain showed enhancement of the left facial nerve; started on prednisone and valacyclovir |
| Porto, Portugal^361^ | 1 | - 35-year-old woman, 39-weeks gestation, presented with 2-days of left facial nerve palsy - Found to be SARS-CoV-2 positive, treated with 10-day prednisolone taper |
| Madrid, Spain^362^ | 1 | - 30-year-old female with acute vestibular dysfunction – vertigo, unsteadiness, nystagmus for 48-hours - MRI clear, SARS-CoV-2 positive, no other symptoms (resolved with symptomatic treatment) |
| Miami, Florida^363^ | 1 | - 32-year-old male, otherwise healthy, with abducens nerve palsy in the setting of SARS-CoV-2 infection - MRI 5 weeks after diplopia onset showed atrophic left lateral rectus, T2 hyperintense, indicating denervation |
| **Neuromuscular Junction Disorders** | | |
| Italy^364^ | 3 | - Three patients (2 men, 1 woman) from 64 to 71-years-old without previous neurological or autoimmune disorders diagnosed with myasthenia gravis 5-7 days after the onset of COVID-19 |
| **Myopathy or Myositis** | | |
| Rome, Italy^365^ | 6 | - Ventilator-dependent SARS-CoV-2-confirmed patients, aged 51-72, developed acute flaccid quadriplegia 6-14 days after first symptoms - Preserved extraocular, mimic and tongue muscles, flaccid quadriplegia with possibilities of little distal movements of the hands, no sensory abnormalities, retained but weak deep tendon reflexes - NCS/EMG showed myopathic abnormalities with fibrillation potentials and rapid recruitment of small, polyphasic motor units in deltoid or biceps, quadriceps, and tibial anterior, reduced CMAP amplitude (below 40%–80% of normal) with markedly prolonged duration. CK was normal to mildly elevated, highest 1274 UI/L. - One died of sepsis, the others all showed improvement 14-21 days later |
| Denmark^366^ | 1 | - 68-year-old male with 8-days of fever, cough, and dyspnea; RT-PCR positive for SARS-CoV-2 - Clinical examination showed severe symmetrical proximal and distal weakness, diffuse muscle wasting, and absent deep tendon reflexes - Clinical and EMG findings indicated critical illness myopathy (CIM), but not GBS |
| New York, USA^367^ | 1 | - 58‐year‐old woman presented with cough, dyspnea, and myalgia - Returned with more severe dyspnea, cough, and severe proximal limb, bulbar, and facial weakness - CK 700, anti-SSA and anti-SAE1 antibodies strongly positive, anti-Ku weakly positive - Muscle biopsy showed perivascular inflammatory infiltration with endomysial extension, regenerating fibers - Diagnosed with COVID-19-associated myositis, treated with 5-day course of intravenous methylprednisolone as well as toculizumab (as IL-6 levels were elevated) |
| **Unclear or mixed CNS/PNS presentations** | | |
| Pointe-à-Pitre, France^368^ | 4 | - Severe COVID-19 in males aged 50–70 with combined CNS-PNS features occurring late after first symptoms - Symptoms included confusion, cognitive dysfunction (memory deficit, frontal syndrome), psychiatric disorders (paranoid delusion, hallucinations), weakness, pyramidal signs, dysautonomia, swallowing dysfunction, vertical supranuclear eye palsy, upper limbs myoclonus, fasciculation and focal muscle atrophy - One patient had a small acute sub-cortical ischemic stroke on brain MRI - CSF showed a normal cell count and a moderate increase of protein level in the up to 80 mg/L in two cases - RT-PCR and IgM for SARS-CoV-2 in the CSF were negative in all patients - Three patients had electrophysiological features of acute motor demyelinating polyradiculoneuropathy with delayed distal latencies and F-waves, slowed conduction velocities and conduction blocks; one had lower motor neuron features in both the upper and lower limbs. - IVIG was given for 5 days - psychiatric symptoms, cognitive impairment and dysautonomia improved thereafter, but myoclonus and motor weakness of the upper limbs persisted 3 weeks after discharge |
| Wroclaw, Poland^369^ | 2 | - Two patients with COVID-19 who reported cutaneous hyperesthesia and became asymptomatic 10 days later; several neuropathic features were present. No specific investigations reported. - They were treated with hydroxychloroquine which had been considered by some to be potentially useful at the dawn of the pandemic |
| Wuhan, China^370^ | 1 | - 64‐year‐old male presented with fever, cough, insomnia, general myalgia - Next day, found lethargic and unresponsive. CT Head unremarkable. - Chest CT and throat swab confirmed COVID-19 - Level of consciousness fluctuated, and patient was confused, with bilateral ankle clonus, left Babinski and bilateral Chaddock sign, slight neck stiffness, Brudzinski sign - CSF unremarkable, SARS-CoV-2 negative; spontaneous improvement of neurological symptoms |
| Wuhan, China^371^ | 1 | - 53-year-old female presenting with dizziness, also noted to have dry throat but no other symptoms - Normal MRI Brain but abnormal chest CT (infiltrations and pleural effusion) – SARS-CoV-2 RT-PCR positive |
| Wroclaw, Poland^372^ | 1 | - 66-year-old man with COVID ‑19 complicated by both ischemic cerebral stroke and cerebral venous thrombosis - Also developed critical illness polyneuropathy - CSF negative for SARS-CoV-2, but positive for enterovirus (enteroviral encephalitis thus an alternative explanation) |

**Supplementary Table 9. Key evidence of potential HEENT and neurological manifestations of COVID-19 in children from cohort studies or large series (>10 patients)**

| **Location** | **Study Type** | **Number of patients** | **Key findings** |
| --- | --- | --- | --- |
| 61 hospitals, USA^373^ | Multi-centre case series | 1,695 (616 with MIS-C) | - Children and adolescents hospitalized with acute COVID-19 or MIS-C captured in Overcoming COVID-19 public health registry - 365 (22%) had neurologic involvement; 22% of these patients had underlying neurologic disorders - 88% of those with neurological symptoms had transient symptoms and survived - 43 (12%) developed COVID-19–related life-threatening neurologic disorders: severe encephalopathy (n=15, 5 with splenial lesions), stroke (n=12), CNS demyelination (n=8), GBS or variants (n=4), and acute fulminant cerebral edema (n=4) - 11 (26%) died, and 17 (40%) survived with new neurologic sequelae - Those with life-threatening symptoms had higher neutrophil-to-lymphocyte ratios and D-dimer |
| 66 hospitals in USA^374^ | Case series | 539 MIS-C, 577 severe COVID-19 | - Headache or altered mental status seen at presentation in 40.4% with MIS-C vs 32.2% with severe COVID-19 - Severe neurological involvement in 12.2% MIS-C vs 19.9% severe COVID-19 (significantly less in MIS-C) - Unclear what “severe” entailed; seizures reported in 2% of MIS-C group and 6% of severe COVID-19 group |
| Wuhan, China^375^ | Retrospective cohort | 216 | - 49 children (22.7%) showed various ocular manifestations, of which 9 had ocular complaints being the initial manifestations of COVID-19 - The common ocular manifestations were conjunctival discharge (27 [55.1%]), eye rubbing (19 [38.8%]), and conjunctival congestion (5 [10.2%]) - Children with systemic symptoms (29.3% vs 14.0%; P = 0.008) or with cough (31.6% vs 17.5%; P = 0.02) were more likely to develop ocular symptoms - Ocular symptoms were typically mild, and children recovered or improved |
| London, United Kingdom^376^ | Case series | 27 | - Children with COVID-19 presenting with new CNS and PNS symptoms and splenial changes on MRI, with no respiratory symptoms - Neurological improvement was seen in all patients, with 2 making a complete recovery by the end of the study |

**Supplementary Table 10. Relevant case reports or brief series (≤10 patients) on potential neurological manifestations of COVID-19 in children.**

| **Location** | **Number of patients** | **Key findings** |
| --- | --- | --- |
| **Stroke – ischemic, hemorrhagic, venous sinus thrombosis** | | |
| New York City, USA^377^ | 2 | - 5-year-old boy with no past medical history presented with fever, cough, and abdominal pain. He progressed to cardiogenic shock and tested positive for COVID-19 - After 5 days CT head revealed a right middle cerebral artery infarction, cerebral edema, and diffuse contralateral subarachnoid hemorrhage - Brain death was confirmed 3 days later following normalization of his electrolytes - 2-month-old boy, tested negative for COVID-19 presented with respiratory failure, pneumomediastinum, and bilateral pneumothoraces. - Electroencephalogram showed non-convulsive status epilepticus - CT revealed bilateral MCA and posterior cerebral artery territory infarctions with hemorrhagic transformation - MRI showed hemorrhagic infarctions (thought to be cardioembolic) in bilateral occipito-parietal lobes, left temporal and left frontal lobes, and stable bilateral subdural collections |
| New Jersey, USA^378^ | 2 | - Two adolescents presented with orbital cellulitis and sinusitis: SARS-CoV-2 positive - MRI Brain/Orbit findings included hemorrhagic abscess with blood of varying age in the first patient, intracranial epidural abscess in the second, radiographic signal consistent with hemorrhagic or thrombotic phenomena, retro-maxillary antral fat changes, and meningeal enhancement or extension in both cases - Radiographic findings thereby mimicked fungal infection, although final cultures and ancillary investigation for allergic and invasive fungal disease remained negative |
| Madrid, Spain^379^ | 1 | - 13-year-old girl with impaired consciousness and severe headache - Found to have multiple cerebral venous sinus thromboses with venous infarction in setting of COVID-19 |
| Brazil^380^ | 1 | - 15-year-old male, presented frontal headaches with retro-orbital pain accompanied by fever evolving to weakness and pain of the lower limbs, which ascended to upper limbs - NCS was compatible with the acute motor axonal neuropathy variant of GBS |
| New York City, USA^381^ | 1 | - 33-month-old boy , positive for SARSCoV-2 presented to the emergency with two days of fever, emesis, and rash - Examination revealed a tachycardic, interactive toddler with erythematous macules on his thighs. - On hospital day 6 during neurology consultation, the patient was somnolent, with slight facial grimace to noxious stimuli, diffuse hypotonia, and significant weakness - MRI brain revealed restricted diffusion in the bilateral lateral thalamic nuclei without T2/FLAIR changes. - The patient’s neurological status continued to improve, no additional neuroprotective strategies were employed |
| Philadelphia, USA^382^ | 1 | - 12 year-old-boy with no previous history of fever, cough, shortness of breath, skin rash, hemoglobinopathy, or recent trauma, initially presented with generalized seizures - Developed right-sided hemiparesis and dysarthria - Nasopharyngeal swab tested positive for SARS-CoV-2 and CSF positive for SARS-CoV-2 - Neurologic findings consistent with cerebral arteriopathy and ischemic stroke |
| **Movement Disorders** | | |
| Madrid, Spain^383^ | 1 | - 26 day-old-male infant with two paroxysmal episodes, upward rolling of the eyes and generalised hypertonia - During his hospital stay the PCR test for detection of SARS-CoV-2 was positive - On Day 6 there was no evidence of convulsive seizures and no findings of the neurologic examination - The infant was discharged with recommendations of maintaining isolation at home |
| New York City, USA^384^ | 1 | - 6-week-old male infant brought to hospital after 1 day of cough, fever, and brief episodes of sustained upward gaze associated with bilateral leg stiffening - PCR nasopharyngeal swab testing was positive for SARSCoV-2 RNA - The patient was discharged home 1 day after admission without further fever or events on follow-up 1 week later |
| **Seizures and Status Epilepticus** | | |
| USA^385^ | 1 | - 8-year-old boy, tested positive for COVID-19, admitted with status epilepticus - Treatment abated the seizure activity and all clinical symptoms resolved within a few hours - 24-hour EEG was abnormal, indicative of diffuse cerebral dysfunction |
| New York, USA^386^ | 1 | - 14-year-old girl presented with 6 days of fever, nasal congestion, myalgia, followed by 3 GTC seizures and perioral cyanosis; tested positive for SARS-CoV-2 RT-PCR - Two days after admission, had two apneic episodes necessitating intubation – EEG monitoring showed epileptiform correlate, neuroimaging was fine - Another apneic episode was associated with lip smacking – EEG showed correlate from right posterior temporal region. Lacosamide was added to levetiracetam with complete resolution of apneic events and extubation |
| New York City, USA^387^ | 1 | - 11-year-old-boy presented with status epilepticus requiring anticonvulsant medications and cerebrospinal fluid evidence for encephalitis - Electroencephalography revealed frontal intermittent delta activity. - Nasopharyngeal swab was positive for COVID-19 as well as rhinovirus/enterovirus but the latter was absent in cerebrospinal fluid polymerase chain reaction - Resolution of symptoms occurred in 6 days, without treatment |
| New York City, USA^388^ | 1 | - 11-year-old-boy presented with abnormal shakiness of the body , associated with stretching and tightening of all four limbs, uprolling of eyes, frothing from the mouth, and tongue bite without urinary or bowel incontinence - Swab PCR positive for SARS-CoV2 - Progressively improved with anti-seizure medications |
| **Guillain-Barre Syndrome** | | |
| Saudi Arabia^389^ | 1 | - 11-year-old boy experienced febrile illness with respiratory manifestations and a persistent cough for 3-weeks before neurological symptoms started, manifestations were consistent with GBS - SARS-CoV-2 confirmed by oropharyngeal swab |

**SUPPLEMENTARY FIGURES**

**
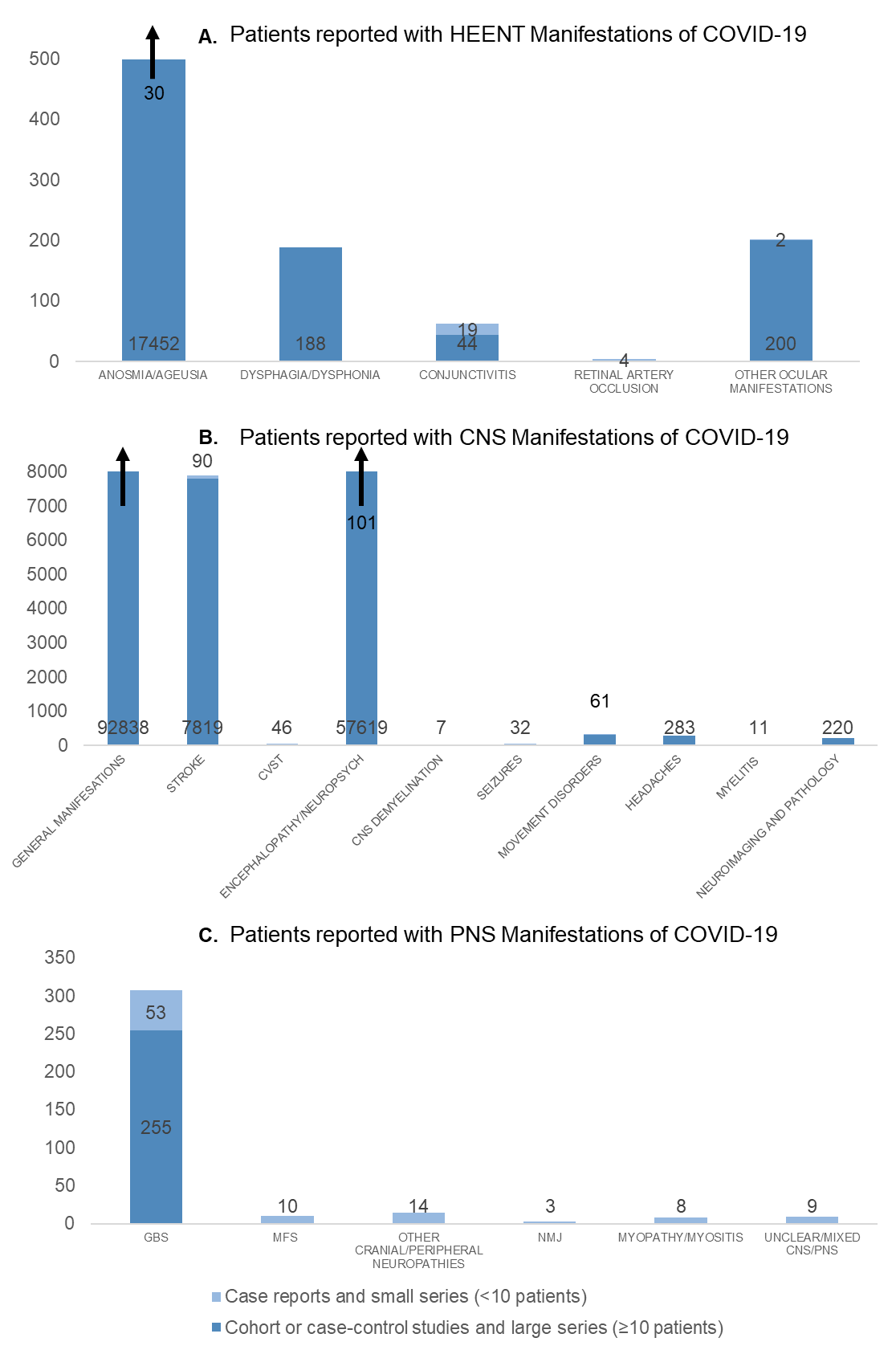
**

**Supplementary Figure 1.** Number of patients reported as of March 7, 2021 with (A) HEENT, (B) central nervous system (CNS), and (C) peripheral nervous system (PNS) manifestations of COVID-19. CVST – Cerebral Venous Sinus Thrombosis, GBS – Guillain-Barre Syndrome, MFS – Miller-Fisher Syndrome, NMJ – Neuromuscular Junction disorders like myasthenia gravis.

**
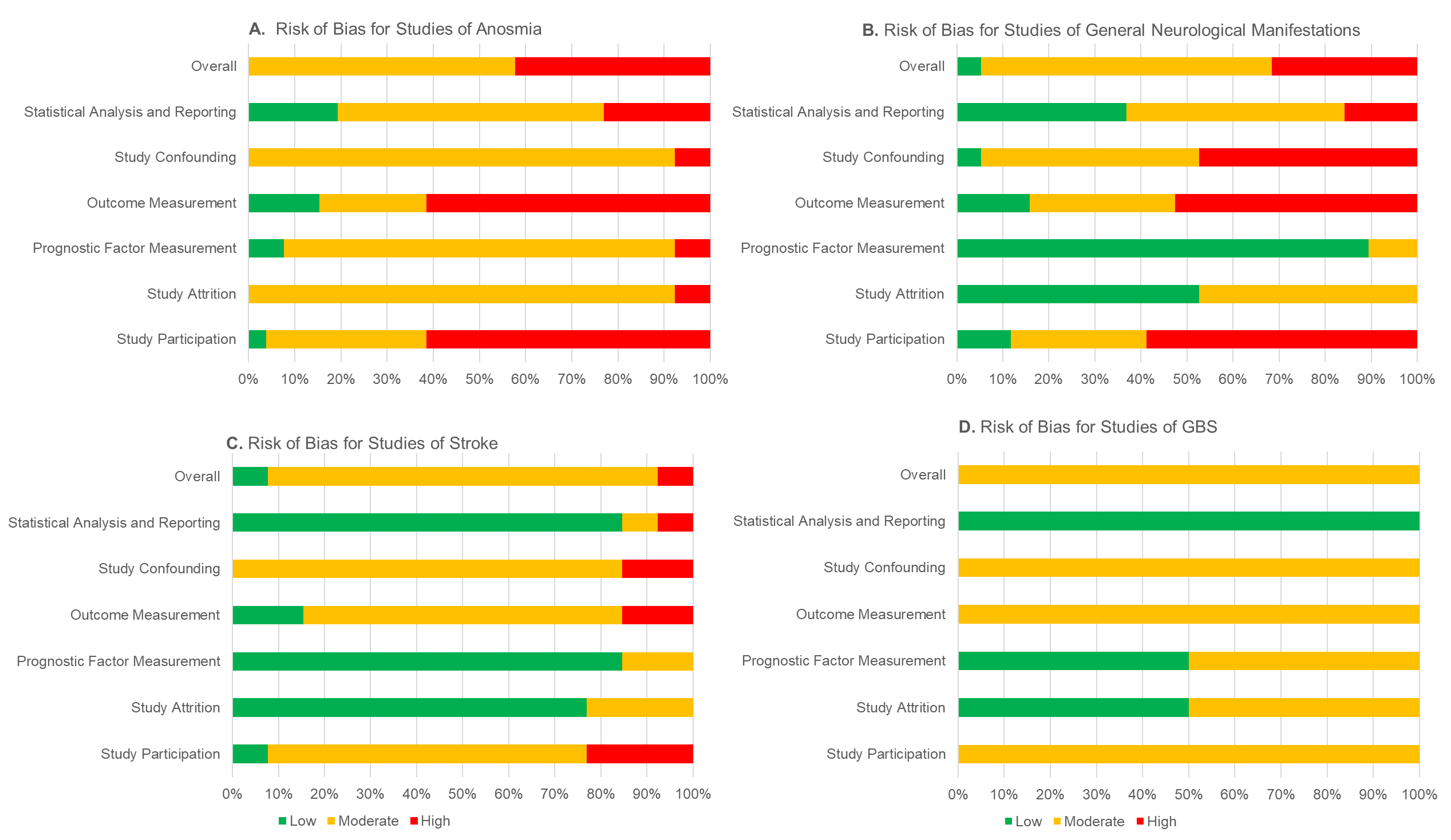
**

**Supplementary Figure 2.** Risk of bias evaluated using the QUIPS tool for studies of (A) anosmia or dysgeusia, (B) general neurological manifestations, and (C) ischemic stroke in patients with COVID-19, that were included in pooled analyses.

**
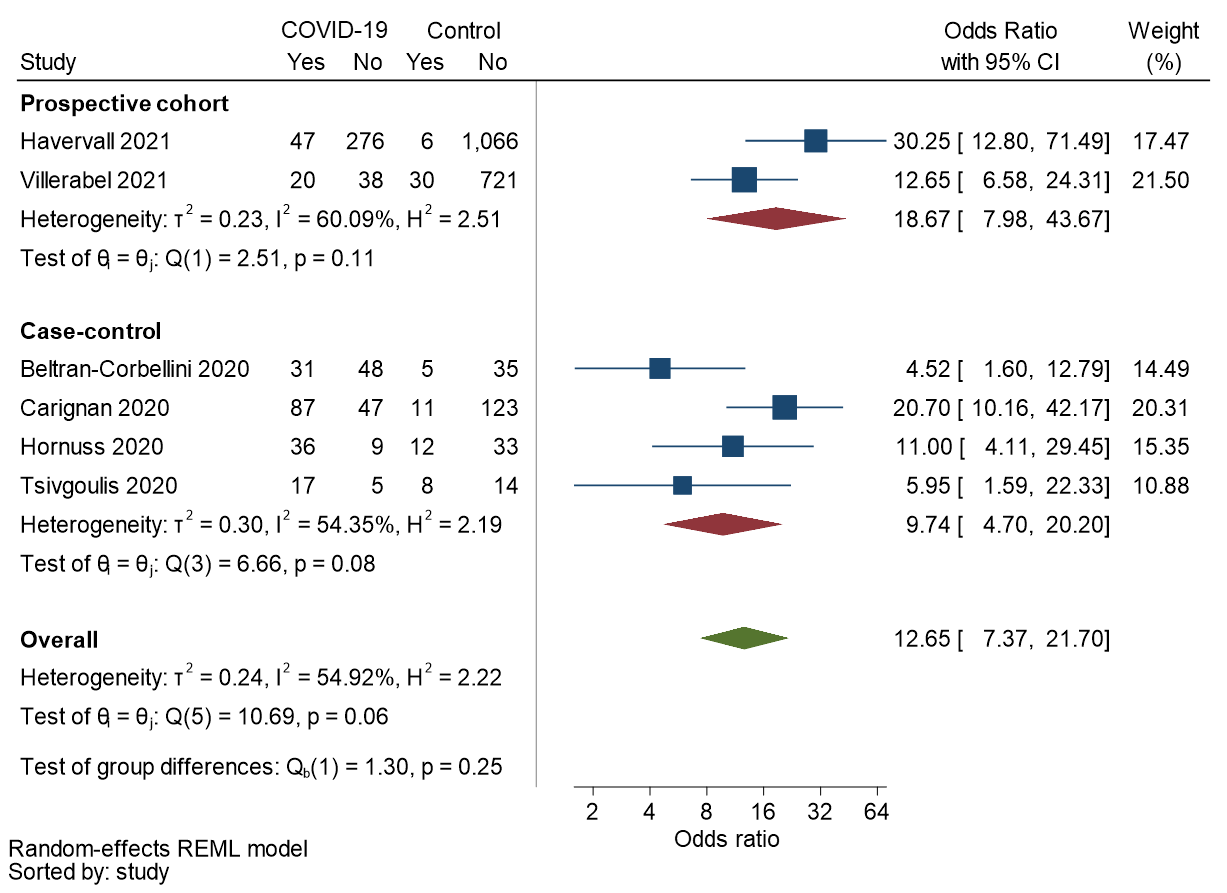
**

**Supplementary Figure 3.** Forest plots from random-effects meta-analyses for studies comparing the occurrence of anosmia and/or ageusia in patients with COVID-19 versus control groups without COVID-19, excluding two studies (Altin 2020 and Chung 2020) as part of a sensitivity analysis. The studies are grouped by their design (case-control studies or prospective cohorts). The “Yes” columns indicate the number of patients in a given group (COVID-19 patients or control) who had anosmia and/or ageusia, while “No” indicates the number of patients in that group who did not have this symptom.

**
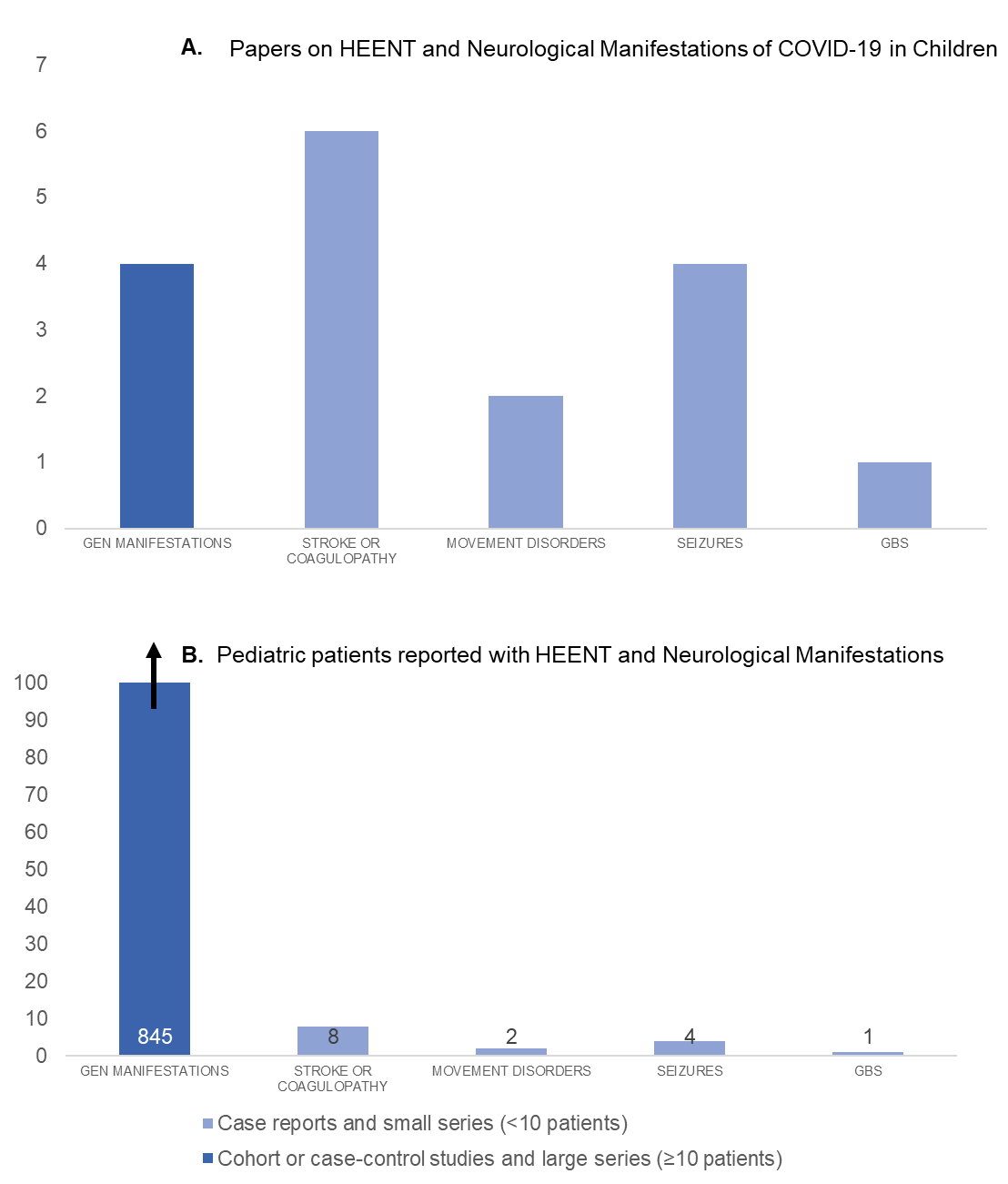
**

**Supplementary Figure 4.** Number of (A) published papers on, and (B) patients reported with, HEENT and neurological manifestations in pediatric cases of COVID-19 as of March 7, 2021. Gen manifestations = General, non-specific HEENT and neurological manifestations.

GBS = Guillain-Barre Syndrome.

**SUPPLEMENTARY REFERENCES**

1. Hwang CS. Olfactory neuropathy in severe acute respiratory syndrome: report of A case. Acta Neurol Taiwan. 2006;15(1):26-8.

2. Umapathi T, Kor AC, Venketasubramanian N, et al. Large artery ischaemic stroke in severe acute respiratory syndrome (SARS). J Neurol. 2004;251(10):1227-31.

3. Tsai LK, Hsieh ST, Chang YC. Neurological manifestations in severe acute respiratory syndrome. Acta Neurol Taiwan. 2005;14(3):113-9.

4. Saad M, Omrani AS, Baig K, et al. Clinical aspects and outcomes of 70 patients with Middle East respiratory syndrome coronavirus infection: a single-center experience in Saudi Arabia. Int J Infect Dis. 2014;29:301-6.

5. Chao CC, Tsai LK, Chiou YH, et al. Peripheral nerve disease in SARS:: report of a case. Neurology. 2003;61(12):1820-1.

6. Tsai LK, Hsieh ST, Chao CC, et al. Neuromuscular disorders in severe acute respiratory syndrome. Arch Neurol. 2004;61(11):1669-73.

7. Leung TW, Wong KS, Hui AC, et al. Myopathic changes associated with severe acute respiratory syndrome: a postmortem case series. Arch Neurol. 2005;62(7):1113-7.

8. Kim JE, Heo JH, Kim HO, et al. Neurological Complications during Treatment of Middle East Respiratory Syndrome. J Clin Neurol. 2017;13(3):227-33.

9. Bagheri SHR, Asghari AM, Farhadi M, et al. Coincidence of COVID-19 epidemic and olfactory dysfunction outbreak. MedRxiv. 2020.

10. Parma V, Ohla K, Veldhuizen MG, et al. More than smell – COVID-19 is associated with severe impairment of smell, taste, and chemesthesis. Chemical Senses. 2020.

11. Lechien JR, Chiesa-Estomba CM, Hans S, Barillari MR, Jouffe L, Saussez S. Loss of Smell and Taste in 2013 European Patients With Mild to Moderate COVID-19. Ann Intern Med. 2020;173(8):672-5.

12. Huang C, Huang L, Wang Y, et al. 6-month consequences of COVID-19 in patients discharged from hospital: a cohort study. Lancet. 2021;397(10270):220-32.

13. Lapostolle F, Schneider E, Vianu I, et al. Clinical features of 1487 COVID-19 patients with outpatient management in the Greater Paris: the COVID-call study. Intern Emerg Med. 2020;15(5):813-7.

14. Salmon D, Bartier S, Hautefort C, et al. Self-reported loss of smell without nasal obstruction to identify COVID-19. The multicenter CORANOSMIA cohort study. The Journal of infection. 2020:S0163-4453(20)30463-1.

15. Lechien JR, Chiesa-Estomba CM, Place S, et al. Clinical and epidemiological characteristics of 1420 European patients with mild-to-moderate coronavirus disease 2019. Journal of Internal Medicine. 2020;288(3):335-44.

16. Gómez-Iglesias P, Porta-Etessam J, Montalvo T, et al. An Online Observational Study of Patients With Olfactory and Gustory Alterations Secondary to SARS-CoV-2 Infection. Front Public Health. 2020;8:243-.

17. Villerabel C, Makinson A, Jaussent A, et al. Diagnostic Value of Patient-Reported and Clinically Tested Olfactory Dysfunction in a Population Screened for COVID-19. JAMA Otolaryngol Head Neck Surg. 2021;147(3):271-9.

18. Chiesa-Estomba CM, Lechien JR, Radulesco T, et al. Patterns of smell recovery in 751 patients affected by the COVID-19 outbreak. Eur J Neurol.n/a(n/a).

19. Joffily L, Ungierowicz A, David AG, et al. The close relationship between sudden loss of smell and COVID-19. Braz J Otorhinolaryngol. 2020:S1808-8694(20)30060-4.

20. Haehner A, Draf J, Dräger S, de With K, Hummel T. Predictive Value of Sudden Olfactory Loss in the Diagnosis of COVID-19. ORL J Otorhinolaryngol Relat Spec. 2020;82(4):175-80.

21. Lechien JR, Chiesa-Estomba CM, De Siati DR, et al. Olfactory and gustatory dysfunctions as a clinical presentation of mild-to-moderate forms of the coronavirus disease (COVID-19): a multicenter European study. Eur Arch Otorhinolaryngol. 2020.

22. Agarwal P, Ray S, Madan A, Tyson B. Neurological manifestations in 404 COVID-19 patients in Washington State. Journal of Neurology. 2020.

23. Qiu C, Cui C, Hautefort C, et al. Olfactory and Gustatory Dysfunction as an Early Identifier of COVID-19 in Adults and Children: An International Multicenter Study. Otolaryngology–Head and Neck Surgery. 2020:0194599820934376.

24. Dell'Era V, Farri F, Garzaro G, Gatto M, Aluffi Valletti P, Garzaro M. Smell and taste disorders during COVID-19 outbreak: Cross-sectional study on 355 patients. Head & neck. 2020;42(7):1591-6.

25. Havervall S, Rosell A, Phillipson M, et al. Symptoms and Functional Impairment Assessed 8 Months After Mild COVID-19 Among Health Care Workers. JAMA. 2021.

26. Villarreal IM, Morato M, Martínez-RuizCoello M, et al. Olfactory and taste disorders in healthcare workers with COVID-19 infection. European archives of oto-rhino-laryngology : official journal of the European Federation of Oto-Rhino-Laryngological Societies (EUFOS) : affiliated with the German Society for Oto-Rhino-Laryngology - Head and Neck Surgery. 2020:1-5.

27. Gorzkowski V, Bevilacqua S, Charmillon A, et al. Evolution of Olfactory Disorders in COVID-19 Patients. The Laryngoscope.n/a(n/a).

28. Mao L, Jin H, Wang M, et al. Neurologic Manifestations of Hospitalized Patients With Coronavirus Disease 2019 in Wuhan, China. JAMA Neurol. 2020.

29. Spinato G, Fabbris C, Polesel J, et al. Alterations in Smell or Taste in Mildly Symptomatic Outpatients With SARS-CoV-2 Infection. JAMA. 2020.

30. Boscolo-Rizzo P, Borsetto D, Fabbris C, et al. Evolution of Altered Sense of Smell or Taste in Patients With Mildly Symptomatic COVID-19. JAMA Otolaryngol Head Neck Surg. 2020.

31. Spadera L, Viola P, Pisani D, et al. Sudden olfactory loss as an early marker of COVID-19: a nationwide Italian survey. European archives of oto-rhino-laryngology : official journal of the European Federation of Oto-Rhino-Laryngological Societies (EUFOS) : affiliated with the German Society for Oto-Rhino-Laryngology - Head and Neck Surgery. 2020:1-9.

32. Sakalli E, Temirbekov D, Bayri E, Alis EE, Erdurak SC, Bayraktaroglu M. Ear nose throat-related symptoms with a focus on loss of smell and/or taste in COVID-19 patients. Am J Otolaryngol. 2020;41(6):102622-.

33. Li J, Long X, Zhu C, et al. Olfactory Dysfunction in Recovered Coronavirus Disease 2019 (COVID-19) Patients. Movement Disorders. 2020;35(7):1100-1.

34. Patel A, Charani E, Ariyanayagam D, et al. New-onset anosmia and ageusia in adult patients diagnosed with SARS-CoV-2 infection. Clin Microbiol Infect. 2020;26(9):1236-41.

35. Biadsee A, Biadsee A, Kassem F, Dagan O, Masarwa S, Ormianer Z. Olfactory and Oral Manifestations of COVID-19: Sex-Related Symptoms-A Potential Pathway to Early Diagnosis. Otolaryngol Head Neck Surg. 2020;163(4):722-8.

36. Vaira LA, Hopkins C, Petrocelli M, et al. Smell and taste recovery in coronavirus disease 2019 patients: a 60-day objective and prospective study. The Journal of Laryngology & Otology. 2020:1-14.

37. Carignan A, Valiquette L, Grenier C, et al. Anosmia and dysgeusia associated with SARS-CoV-2 infection: an age-matched case–control study. Canadian Medical Association Journal. 2020;192(26):E702-E7.

38. Abalo-Lojo JM, Pouso-Diz JM, Gonzalez F. Taste and Smell Dysfunction in COVID-19 Patients. Annals of Otology, Rhinology & Laryngology. 2020;129(10):1041-2.

39. Cocco A, Amami P, Desai A, Voza A, Ferreli F, Albanese A. Neurological features in SARS-CoV-2-infected patients with smell and taste disorder. Journal of Neurology. 2020:1-3.

40. Jalessi M, Barati M, Rohani M, et al. Frequency and outcome of olfactory impairment and sinonasal involvement in hospitalized patients with COVID-19. Neurological sciences : official journal of the Italian Neurological Society and of the Italian Society of Clinical Neurophysiology. 2020;41(9):2331-8.

41. Hornuss D, Lange B, Schröter N, Rieg S, Kern WV, Wagner D. Anosmia in COVID-19 patients. Clin Microbiol Infect. 2020:S1198-743X(20)30294-9.

42. Lechien JR, Cabaraux P, Chiesa-Estomba CM, et al. Objective olfactory evaluation of self-reported loss of smell in a case series of 86 COVID-19 patients. Head & neck. 2020;42(7):1583-90.

43. Altin F, Cingi C, Uzun T, Bal C. Olfactory and gustatory abnormalities in COVID-19 cases. European archives of oto-rhino-laryngology : official journal of the European Federation of Oto-Rhino-Laryngological Societies (EUFOS) : affiliated with the German Society for Oto-Rhino-Laryngology - Head and Neck Surgery. 2020:1-7.

44. Beltrán-Corbellini Á, Chico-García JL, Martínez-Poles J, et al. Acute-onset smell and taste disorders in the context of COVID-19: a pilot multicentre polymerase chain reaction based case-control study. Eur J Neurol. 2020:10.1111/ene.14273.

45. Lechien JR, Cabaraux P, Chiesa-Estomba CM, et al. Psychophysical Olfactory Tests and Detection of COVID-19 in Patients With Sudden Onset Olfactory Dysfunction: A Prospective Study. Ear, Nose & Throat Journal. 2020:0145561320929169.

46. Luers JC, Rokohl AC, Loreck N, et al. Olfactory and Gustatory Dysfunction in Coronavirus Disease 2019 (COVID-19). Clinical Infectious Diseases. 2020.

47. Klopfenstein T, Zahra H, Kadiane-Oussou NdJ, et al. New loss of smell and taste: Uncommon symptoms in COVID-19 patients on Nord Franche-Comte cluster, France. International Journal of Infectious Diseases. 2020.

48. Giacomelli A, Pezzati L, Conti F, et al. Self-reported olfactory and taste disorders in SARS-CoV-2 patients: a cross-sectional study. Clin Infect Dis. 2020.

49. Tsivgoulis G, Fragkou PC, Delides A, et al. Quantitative evaluation of olfactory dysfunction in hospitalized patients with Coronavirus [2] (COVID-19). Journal of neurology. 2020;267(8):2193-5.

50. Chung TW-H, Sridhar S, Zhang AJ, et al. Olfactory Dysfunction in Coronavirus Disease 2019 Patients: Observational Cohort Study and Systematic Review. Open Forum Infect Dis. 2020;7(6):ofaa199-ofaa.

51. Vargas-Gandica J, Winter D, Schnippe R, et al. Ageusia and anosmia, a common sign of COVID-19? A case series from four countries. J Neurovirol. 2020:1-5.

52. Lechien JR, Chiesa-Estomba CM, Cabaraux P, et al. Features of Mild-to-Moderate COVID-19 Patients With Dysphonia. J Voice. 2020.

53. Guan WJ, Ni ZY, Hu Y, et al. Clinical Characteristics of Coronavirus Disease 2019 in China. N Engl J Med. 2020.

54. Sindhuja K, Lomi N, Asif MI, Tandon R. Clinical profile and prevalence of conjunctivitis in mild COVID-19 patients in a tertiary care COVID-19 hospital: A retrospective cross-sectional study. Indian J Ophthalmol. 2020;68(8):1546-50.

55. Zhou Y, Duan C, Zeng Y, et al. Ocular Findings and Proportion with Conjunctival SARS-COV-2 in COVID-19 Patients. Ophthalmology. 2020;127(7):982-3.

56. Zhang X, Chen X, Chen L, et al. The evidence of SARS-CoV-2 infection on ocular surface. Ocul Surf. 2020;18(3):360-2.

57. Wu P, Duan F, Luo C, et al. Characteristics of Ocular Findings of Patients With Coronavirus Disease 2019 (COVID-19) in Hubei Province, China. JAMA Ophthalmol. 2020.

58. Xia J, Tong J, Liu M, Shen Y, Guo D. Evaluation of coronavirus in tears and conjunctival secretions of patients with SARS-CoV-2 infection. J Med Virol. 2020.

59. Chen L, Deng C, Chen X, et al. Ocular manifestations and clinical characteristics of 535 cases of COVID-19 in Wuhan, China: a cross-sectional study. Acta Ophthalmol. 2020:10.1111/aos.14472.

60. Öncül H, Öncül FY, Alakus MF, Çağlayan M, Dag U. Ocular findings in patients with coronavirus disease 2019 (COVID-19) in an outbreak hospital. J Med Virol. 2020.

61. Gangaputra SS, Patel SN. Ocular Symptoms among Nonhospitalized Patients Who Underwent COVID-19 Testing. Ophthalmology. 2020:S0161-6420(20)30574-1.

62. Abrishami M, Tohidinezhad F, Daneshvar R, et al. Ocular Manifestations of Hospitalized Patients with COVID-19 in Northeast of Iran. Ocul Immunol Inflamm. 2020;28(5):739-44.

63. Hong N, Yu W, Xia J, Shen Y, Yap M, Han W. Evaluation of ocular symptoms and tropism of SARS-CoV-2 in patients confirmed with COVID-19. Acta Ophthalmol. 2020:10.1111/aos.14445.

64. Marinho PM, Marcos AAA, Romano AC, Nascimento H, Belfort R, Jr. Retinal findings in patients with COVID-19. Lancet (London, England). 2020;395(10237):1610-.

65. Gilani S, Roditi R, Naraghi M. COVID-19 and anosmia in Tehran, Iran. Med Hypotheses. 2020;141:109757-.

66. Brookes NRG, Fairley JW, Brookes GB. Acute Olfactory Dysfunction—A Primary Presentation of COVID-19 Infection. Ear, Nose & Throat Journal. 2020:0145561320940119.

67. Politi LS, Salsano E, Grimaldi M. Magnetic Resonance Imaging Alteration of the Brain in a Patient With Coronavirus Disease 2019 (COVID-19) and Anosmia. JAMA Neurology. 2020;77(8):1028-9.

68. Lechner M, Chandrasekharan D, Jumani K, et al. Anosmia as a presenting symptom of SARS-CoV-2 infection in healthcare workers - A systematic review of the literature, case series, and recommendations for clinical assessment and management. Rhinology. 2020.

69. Distinguin L, Ammar A, Lechien JR, et al. MRI of Patients Infected With COVID-19 Revealed Cervical Lymphadenopathy. Ear Nose Throat J. 2020:145561320940117.

70. Kirschenbaum D, Imbach LL, Ulrich S, et al. Inflammatory olfactory neuropathy in two patients with COVID-19. Lancet. 2020;396(10245):166.

71. Lee JM, Lee SJ. Olfactory and Gustatory Dysfunction in a COVID-19 Patient with Ankylosing Spondylitis Treated with Etanercept: Case Report. Journal of Korean medical science. 2020;35(21):e201-e.

72. Jacob J, Flannery W, Mostert C. Novel ENT triad of anosmia, ageusia and hearing impairment in COVID-19. Internal Medicine Journal.n/a(n/a).

73. Gane SB, Kelly C, Hopkins C. Isolated sudden onset anosmia in COVID-19 infection. A novel syndrome? Rhinology. 2020;58(3):299-301.

74. Laurendon T, Radulesco T, Mugnier J, et al. Bilateral transient olfactory bulb edema during COVID-19–related anosmia. Neurology. 2020;95(5):224-5.

75. Eliezer M, Hautefort C, Hamel A-L, et al. Sudden and Complete Olfactory Loss of Function as a Possible Symptom of COVID-19. JAMA Otolaryngology–Head & Neck Surgery. 2020;146(7):674-5.

76. Melley LE, Bress E, Polan E. Hypogeusia as the initial presenting symptom of COVID-19. BMJ Case Rep. 2020;13(5).

77. Ismail, II, Gad KA. Absent Blood Oxygen Level-Dependent Functional Magnetic Resonance Imaging Activation of the Orbitofrontal Cortex in a Patient With Persistent Cacosmia and Cacogeusia After COVID-19 Infection. JAMA Neurol. 2021.

78. Scalinci SZ, Trovato Battagliola E. Conjunctivitis can be the only presenting sign and symptom of COVID-19. IDCases. 2020;20:e00774-e.

79. Deng C, Chen L, Chen X, Zhang X, Chen B, Sun X. Documenting Course of 2 Cases of Conjunctivitis in Mobile Hospitals During the Coronavirus Disease 2019 Pandemic. JAMA Ophthalmology. 2020.

80. Daruich A, Martin D, Bremond-Gignac D. Unilateral conjunctivitis as first presentation of Coronavirus Disease 2019 (COVID-19): A telemedicine diagnosis. J Fr Ophtalmol. 2020;43(5):e167-e8.

81. Navel V, Chiambaretta F, Dutheil F. Haemorrhagic conjunctivitis with pseudomembranous related to SARS-CoV-2. Am J Ophthalmol Case Rep. 2020;19:100735-.

82. Cheema M, Aghazadeh H, Nazarali S, et al. Keratoconjunctivitis as the initial medical presentation of the novel coronavirus disease 2019 (COVID-19). Can J Ophthalmol. 2020;55(4):e125-e9.

83. Ozturker ZK. Conjunctivitis as sole symptom of COVID-19: A case report and review of literature. Eur J Ophthalmol. 2020:1120672120946287-.

84. Ying NY, Idris NS, Muhamad R, Ahmad I. Coronavirus Disease 2019 Presenting as Conjunctivitis. J Korean Acad Fam Med. 2020;0(0).

85. Nayak B, Poddar C, Panigrahi MK, Tripathy S, Mishra B. Late manifestation of follicular conjunctivitis in ventilated patient following COVID-19 positive severe pneumonia. Indian J Ophthalmol. 2020;68(8):1675-7.

86. Salducci M, La Torre G. COVID-19 emergency in the cruise's ship: a case report of conjunctivitis. Clin Ter. 2020;171(3):e189-e91.

87. Chen L, Liu M, Zhang Z, et al. Ocular manifestations of a hospitalised patient with confirmed 2019 novel coronavirus disease. Br J Ophthalmol. 2020.

88. Khavandi S, Tabibzadeh E, Naderan M, Shoar S. Corona virus disease-19 (COVID-19) presenting as conjunctivitis: atypically high-risk during a pandemic. Cont Lens Anterior Eye. 2020;43(3):211-2.

89. Guo D, Xia J, Wang Y, Zhang X, Shen Y, Tong JP. Relapsing viral keratoconjunctivitis in COVID-19: a case report. Virol J. 2020;17(1):97.

90. Bettach E, Zadok D, Weill Y, Brosh K, Hanhart J. Bilateral anterior uveitis as a part of a multisystem inflammatory syndrome secondary to COVID-19 infection. J Med Virol. 2020.

91. Méndez Mangana C, Barraquer Kargacin A, Barraquer RI. Episcleritis as an ocular manifestation in a patient with COVID-19. Acta Ophthalmol.n/a(n/a).

92. Acharya S, Diamond M, Anwar S, Glaser A, Tyagi P. Unique case of central retinal artery occlusion secondary to COVID-19 disease. IDCases. 2020;21:e00867-e.

93. Dumitrascu OM, Volod O, Bose S, Wang Y, Biousse V, Lyden PD. Acute ophthalmic artery occlusion in a COVID-19 patient on apixaban. J Stroke Cerebrovasc Dis. 2020;29(8):104982-.

94. Selvaraj V, Sacchetti D, Finn A, Dapaah-Afriyie K. Acute Vision Loss in a Patient with COVID-19. R I Med J (2013). 2020;103(6):37-8.

95. Insausti-Garcia A, Reche-Sainz JA, Ruiz-Arranz C, Lopez Vazquez A, Ferro-Osuna M. Papillophlebitis in a COVID-19 patient: Inflammation and hypercoagulable state. Eur J Ophthalmol. 2020:1120672120947591.

96. Virgo J, Mohamed M. Paracentral acute middle maculopathy and acute macular neuroretinopathy following SARS-CoV-2 infection. Eye (Lond). 2020;34(12):2352-3.

97. Taquet M, Geddes JR, Husain M, Luciano S, Harrison PJ. 6-month neurological and psychiatric outcomes in 236 379 survivors of COVID-19: a retrospective cohort study using electronic health records. Lancet Psychiatry. 2021.

98. Nalleballe K, Reddy Onteddu S, Sharma R, et al. Spectrum of neuropsychiatric manifestations in COVID-19. Brain Behav Immun. 2020;88:71-4.

99. Eskandar EN, Altschul DJ, de La Garza Ramos R, et al. Neurologic Syndromes Predict Higher In-Hospital Mortality in COVID-19. Neurology. 2020.

100. Frontera JA, Sabadia S, Lalchan R, et al. A Prospective Study of Neurologic Disorders in Hospitalized Patients With COVID-19 in New York City. Neurology. 2021;96(4):e575-e86.

101. Studart-Neto A, Guedes BF, Tuma RLE, et al. Neurological consultations and diagnoses in a large, dedicated COVID-19 university hospital. Arq Neuropsiquiatr. 2020;78(8):494-500.

102. Xiong W, Mu J, Guo J, et al. New onset neurologic events in people with COVID-19 infection in three regions in China. Neurology. 2020:10.1212/WNL.0000000000010034.

103. Romero-Sánchez CM, Díaz-Maroto I, Fernández-Díaz E, et al. Neurologic manifestations in hospitalized patients with COVID-19. The ALBACOVID registry. 2020;95(8):e1060-e70.

104. Shu L, Wang X, Li M, et al. Clinical characteristics of moderate COVID-19 patients aggravation in Wuhan Stadium Cabin Hospital: A 571 cases of retrospective cohort study. Journal of Medical Virology. 2020;n/a(n/a).

105. Wei Y, Zeng W, Huang X, et al. Clinical characteristics of 276 hospitalized patients with coronavirus disease 2019 in Zengdu District, Hubei Province: a single-center descriptive study. BMC infectious diseases. 2020;20(1):549-.

106. Karadaş Ö, Öztürk B, Sonkaya AR. A prospective clinical study of detailed neurological manifestations in patients with COVID-19. Neurological sciences : official journal of the Italian Neurological Society and of the Italian Society of Clinical Neurophysiology. 2020;41(8):1991-5.

107. Luigetti M, Iorio R, Bentivoglio AR, et al. Assessment of neurological manifestations in hospitalized patients with COVID-19. Eur J Neurol.n/a(n/a).

108. Wang D, Hu B, Hu C, et al. Clinical Characteristics of 138 Hospitalized Patients With 2019 Novel Coronavirus-Infected Pneumonia in Wuhan, China. JAMA. 2020.

109. Chen T, Wu D, Chen H, et al. Clinical characteristics of 113 deceased patients with coronavirus disease 2019: retrospective study. BMJ. 2020;368:m1091.

110. Vacchiano V, Riguzzi P, Volpi L, et al. Early neurological manifestations of hospitalized COVID-19 patients. Neurological sciences : official journal of the Italian Neurological Society and of the Italian Society of Clinical Neurophysiology. 2020;41(8):2029-31.

111. Mahammedi A, Saba L, Vagal A, et al. Imaging in Neurological Disease of Hospitalized COVID-19 Patients: An Italian Multicenter Retrospective Observational Study. Radiology. 2020:201933.

112. Liguori C, Pierantozzi M, Spanetta M, et al. Subjective neurological symptoms frequently occur in patients with SARS-CoV2 infection. Brain Behav Immun. 2020;88:11-6.

113. Garcia-Monco JC, Cabrera Muras A, Erburu Iriarte M, et al. Neurological Manifestations in a Prospective Unselected Series of Hospitalized COVID-19 patients. Neurology: Clinical Practice. 2020:10.1212/CPJ.0000000000000913.

114. Chen N, Zhou M, Dong X, et al. Epidemiological and clinical characteristics of 99 cases of 2019 novel coronavirus pneumonia in Wuhan, China: a descriptive study. Lancet. 2020;395(10223):507-13.

115. Anand P, Zhou L, Bhadelia N, Hamer DH, Greer DM, Cervantes-Arslanian AM. Neurologic findings among inpatients with COVID-19 at a safety-net US hospital. Neurol Clin Pract. 2020.

116. Fan S, Xiao M, Han F, et al. Neurological Manifestations in Critically Ill Patients With COVID-19: A Retrospective Study. Front Neurol. 2020;11:806-.

117. Kremer S, Lersy F, Anheim M, et al. Neurologic and neuroimaging findings in COVID-19 patients: A retrospective multicenter study. Neurology. 2020:10.1212/WNL.0000000000010112.

118. Benussi A, Pilotto A, Premi E, et al. Clinical characteristics and outcomes of inpatients with neurologic disease and COVID-19 in Brescia, Lombardy, Italy. Neurology. 2020;95(7):e910-e20.

119. Sun L, Shen L, Fan J, et al. Clinical features of patients with coronavirus disease 2019 from a designated hospital in Beijing, China. Journal of Medical Virology.n/a(n/a).

120. Pinna P, Grewal P, Hall JP, et al. Neurological manifestations and COVID-19: Experiences from a tertiary care center at the Frontline. Journal of the Neurological Sciences. 2020;415.

121. Zhong Z-F, Huang J, Yang X, et al. Epidemiological and clinical characteristics of COVID-19 patients in Hengyang, Hunan Province, China. World J Clin Cases. 2020;8(12):2554-65.

122. Yousaf AR, Duca LM, Chu V, et al. A prospective cohort study in non-hospitalized household contacts with SARS-CoV-2 infection: symptom profiles and symptom change over time. Clin Infect Dis. 2020.

123. Huang C, Wang Y, Li X, et al. Clinical features of patients infected with 2019 novel coronavirus in Wuhan, China. Lancet. 2020;395(10223):497-506.

124. Ora J, Liguori C, Puxeddu E, et al. Dyspnea perception and neurological symptoms in non-severe COVID-19 patients. Neurol Sci. 2020;41(10):2671-4.

125. Shahjouei S, Naderi S, Li J, et al. Risk of stroke in hospitalized SARS-CoV-2 infected patients: A multinational study. EBioMedicine. 2020;59:102939.

126. Khandelwal P, Al-Mufti F, Tiwari A, et al. Incidence, Characteristics and Outcomes of Large Vessel Stroke in COVID-19 Cohort: An International Multicenter Study. Neurosurgery. 2021.

127. Yaghi S, Ishida K, Torres J, et al. SARS-CoV-2 and Stroke in a New York Healthcare System. Stroke. 2020;51(7):2002-11.

128. Bilaloglu S, Aphinyanaphongs Y, Jones S, Iturrate E, Hochman J, Berger JS. Thrombosis in Hospitalized Patients With COVID-19 in a New York City Health System. JAMA. 2020;324(8):799-801.

129. Merkler AE, Parikh NS, Mir S, et al. Risk of Ischemic Stroke in Patients With Coronavirus Disease 2019 (COVID-19) vs Patients With Influenza. JAMA Neurology. 2020.

130. Hernández-Fernández F, Valencia HS, Barbella-Aponte RA, et al. Cerebrovascular disease in patients with COVID-19: neuroimaging, histological and clinical description. Brain. 2020.

131. Siegler JE, Cardona P, Arenillas JF, et al. EXPRESS: Cerebrovascular events and outcomes in hospitalized patients with COVID-19: The SVIN COVID-19 Multinational Registry. Int J Stroke. 2020:1747493020959216.

132. Srivastava PK, Zhang S, Xian Y, et al. Acute Ischemic Stroke in Patients With COVID-19: An Analysis From Get With The Guidelines-Stroke. Stroke. 2021:STROKEAHA121034301.

133. Rothstein A, Oldridge O, Schwennesen H, Do D, Cucchiara BL. Acute Cerebrovascular Events in Hospitalized COVID-19 Patients. Stroke. 2020;51(9):e219-e22.

134. Kihira S, Schefflein J, Mahmoudi K, et al. Association of Coronavirus Disease (COVID-19) With Large Vessel Occlusion Strokes: A Case-Control Study. American Journal of Roentgenology. 2020:1-6.

135. Li Y, Li M, Wang M, et al. Acute cerebrovascular disease following COVID-19: a single center, retrospective, observational study. Stroke Vasc Neurol. 2020;5(3):279-84.

136. Helms J, Tacquard C, Severac F, et al. High risk of thrombosis in patients with severe SARS-CoV-2 infection: a multicenter prospective cohort study. Intensive Care Med. 2020;46(6):1089-98.

137. Majidi S, Fifi JT, Ladner TR, et al. Emergent Large Vessel Occlusion Stroke During New York City&#x2019;s COVID-19 Outbreak. Stroke. 2020;51(9):2656-63.

138. Dogra S, Jain R, Cao M, et al. Hemorrhagic stroke and anticoagulation in COVID-19. J Stroke Cerebrovasc Dis. 2020;29(8):104984-.

139. Khan M, Ibrahim RHM, Siddiqi SA, et al. COVID-19 and acute ischemic stroke – A case series from Dubai, UAE. International Journal of Stroke. 2020;15(6):699-700.

140. Sweid A, Hammoud B, Bekelis K, et al. Cerebral ischemic and hemorrhagic complications of coronavirus disease 2019. Int J Stroke. 2020;15(7):733-42.

141. Nauen DW, Hooper JE, Stewart CM, Solomon IH. Assessing Brain Capillaries in Coronavirus Disease 2019. JAMA Neurol. 2021.

142. Immovilli P, Terracciano C, Zaino D, et al. Stroke in COVID-19 patients—A case series from Italy. International Journal of Stroke. 2020;15(6):701-2.

143. Nawabi J, Morotti A, Wildgruber M, et al. Clinical and Imaging Characteristics in Patients with SARS-CoV-2 Infection and Acute Intracranial Hemorrhage. Journal of Clinical Medicine. 2020;9(8).

144. Tiwari A, Berekashvili K, Vulkanov V, et al. Etiologic Subtypes of Ischemic Stroke in SARS-CoV-2 Patients in a Cohort of New York City Hospitals. Front Neurol. 2020;11:1004.

145. Conklin J, Frosch MP, Mukerji SS, et al. Susceptibility-weighted imaging reveals cerebral microvascular injury in severe COVID-19. J Neurol Sci. 2021;421:117308.

146. Lee MH, Perl DP, Nair G, et al. Microvascular Injury in the Brains of Patients with Covid-19. N Engl J Med. 2021;384(5):481-3.

147. Franceschi AM, Arora R, Wilson R, Giliberto L, Libman RB, Castillo M. Neurovascular Complications in COVID-19 Infection: Case Series. AJNR Am J Neuroradiol. 2020;41(9):1632-40.

148. Agarwal S, Jain R, Dogra S, et al. Cerebral Microbleeds and Leukoencephalopathy in Critically Ill Patients With COVID-19. Stroke. 2020;51(9):2649-55.

149. Cecchetti G, Vabanesi M, Chieffo R, et al. Cerebral involvement in COVID-19 is associated with metabolic and coagulation derangements: an EEG study. J Neurol. 2020;267(11):3130-4.

150. Perlis RH, Ognyanova K, Santillana M, et al. Association of Acute Symptoms of COVID-19 and Symptoms of Depression in Adults. JAMA Netw Open. 2021;4(3):e213223.

151. Mazza MG, De Lorenzo R, Conte C, et al. Anxiety and depression in COVID-19 survivors: Role of inflammatory and clinical predictors. Brain Behav Immun. 2020.

152. Khan SH, Lindroth H, Perkins AJ, et al. Delirium Incidence, Duration, and Severity in Critically Ill Patients With Coronavirus Disease 2019. Crit Care Explor. 2020;2(12):e0290.

153. Varatharaj A, Thomas N, Ellul MA, et al. Neurological and neuropsychiatric complications of COVID-19 in 153 patients: a UK-wide surveillance study. The Lancet Psychiatry.

154. Poloni TE, Carlos AF, Cairati M, et al. Prevalence and prognostic value of <em>Delirium</em> as the initial presentation of COVID-19 in the elderly with dementia: An Italian retrospective study. EClinicalMedicine.

155. Lu L, Xiong W, Liu D, et al. New onset acute symptomatic seizure and risk factors in coronavirus disease 2019: A retrospective multicenter study. Epilepsia. 2020;61(6):e49-e53.

156. Galanopoulou AS, Ferastraoaru V, Correa DJ, et al. EEG findings in acutely ill patients investigated for SARS-CoV-2/COVID-19: A small case series preliminary report. Epilepsia Open. 2020;5(2):314-24.

157. Chan JL, Murphy KA, Sarna JR. Myoclonus and cerebellar ataxia associated with COVID-19: a case report and systematic review. J Neurol. 2021.

158. Trigo J, Garcia-Azorin D, Planchuelo-Gomez A, et al. Factors associated with the presence of headache in hospitalized COVID-19 patients and impact on prognosis: a retrospective cohort study. J Headache Pain. 2020;21(1):94.

159. Porta-Etessam J, Matias-Guiu JA, Gonzalez-Garcia N, et al. Spectrum of Headaches Associated With SARS-CoV-2 Infection: Study of Healthcare Professionals. Headache. 2020;60(8):1697-704.

160. Xu XW, Wu XX, Jiang XG, et al. Clinical findings in a group of patients infected with the 2019 novel coronavirus (SARS-Cov-2) outside of Wuhan, China: retrospective case series. BMJ. 2020;368:m606.

161. Toptan T, Aktan C, Basari A, Bolay H. Case Series of Headache Characteristics in COVID-19: Headache Can Be an Isolated Symptom. Headache. 2020;60(8):1788-92.

162. Klironomos S, Tzortzakakis A, Kits A, et al. Nervous System Involvement in COVID-19: Results from a Retrospective Consecutive Neuroimaging Cohort. Radiology. 2020:202791.

163. Pons-Escoda A, Naval-Baudín P, Majós C, et al. Neurologic Involvement in COVID-19: Cause or Coincidence? A Neuroimaging Perspective. American Journal of Neuroradiology. 2020;41(8):1365.

164. Chougar L, Shor N, Weiss N, et al. Retrospective Observational Study of Brain Magnetic Resonance Imaging Findings in Patients with Acute SARS-CoV-2 Infection and Neurological Manifestations. Radiology. 2020:202422-.

165. Schiaffino MT, Di Natale M, Garcia-Martinez E, et al. Immunoserologic Detection and Diagnostic Relevance of Cross-Reactive Autoantibodies in Coronavirus Disease 2019 Patients. J Infect Dis. 2020;222(9):1439-43.

166. Kandemirli SG, Dogan L, Sarikaya ZT, et al. Brain MRI Findings in Patients in the Intensive Care Unit with COVID-19 Infection. Radiology.0(0):201697.

167. Matschke J, Lutgehetmann M, Hagel C, et al. Neuropathology of patients with COVID-19 in Germany: a post-mortem case series. Lancet Neurol. 2020;19(11):919-29.

168. Kremer PS, Lersy F, Sèze PJd, et al. Brain MRI Findings in Severe COVID-19: A Retrospective Observational Study. Radiology.0(0):202222.

169. Bellon M, Schweblin C, Lambeng N, et al. Cerebrospinal fluid features in SARS-CoV-2 RT-PCR positive patients. Clinical Infectious Diseases. 2020.

170. Sierra-Hidalgo F, Muñoz-Rivas N, Torres Rubio P, et al. Large artery ischemic stroke in severe COVID-19. Journal of Neurology. 2020.

171. Morassi M, Bagatto D, Cobelli M, et al. Stroke in patients with SARS-CoV-2 infection: case series. Journal of neurology. 2020;267(8):2185-92.

172. Beyrouti R, Adams ME, Benjamin L, et al. Characteristics of ischaemic stroke associated with COVID-19. Journal of Neurology, Neurosurgery, and Psychiatry. 2020.

173. Ashrafi F, Zali A, Ommi D, et al. COVID-19-related strokes in adults below 55 years of age: a case series. Neurological sciences : official journal of the Italian Neurological Society and of the Italian Society of Clinical Neurophysiology. 2020;41(8):1985-9.

174. Oxley TJ, Mocco J, Majidi S, et al. Large-Vessel Stroke as a Presenting Feature of Covid-19 in the Young. N Engl J Med. 2020.

175. Benger M, Williams O, Siddiqui J, Sztriha L. Intracerebral haemorrhage and COVID-19: Clinical characteristics from a case series. Brain Behav Immun. 2020;88:940-4.

176. Barrios-López JM, Rego-García I, Muñoz Martínez C, et al. Ischaemic stroke and SARS-CoV-2 infection: a causal or incidental association? Neurología (English Edition). 2020;35(5):295-302.

177. Diaz-Segarra N, Edmond A, Kunac A, Yonclas P. COVID-19 Ischemic Strokes as an Emerging Rehabilitation Population: A Case Series. Am J Phys Med Rehabil. 2020:10.1097/PHM.0000000000001532.

178. TunÇ A, ÜnlÜbaŞ Y, Alemdar M, AkyÜz E. Coexistence of COVID-19 and acute ischemic stroke report of four cases. Journal of clinical neuroscience : official journal of the Neurosurgical Society of Australasia. 2020;77:227-9.

179. Zhang Y, Xiao M, Zhang S, et al. Coagulopathy and Antiphospholipid Antibodies in Patients with Covid-19. N Engl J Med. 2020.

180. Goncalves B, Righy C, Kurtz P. Thrombotic and Hemorrhagic Neurological Complications in Critically Ill COVID-19 Patients. Neurocrit Care. 2020;33(2):587-90.

181. Sharifi-Razavi A, Karimi N, Zarvani A, Cheraghmakani H, Baghbanian SM. Ischemic stroke associated with novel coronavirus 2019: a report of three cases. Int J Neurosci. 2020:1-5.

182. Heman-Ackah SM, Su YS, Spadola M, et al. Neurologically Devastating Intraparenchymal Hemorrhage in COVID-19 Patients on Extracorporeal Membrane Oxygenation: A Case Series. Neurosurgery. 2020;87(2):E147-E51.

183. Carroll E, Lewis A. Catastrophic Intracranial Hemorrhage in Two Critically Ill Patients with COVID-19. Neurocrit Care. 2020.

184. Gulko E, Gomes W, Ali S, Al-Mufti F, Mehta H. Acute Common Carotid Artery Bifurcation Thrombus: An Emerging Pattern of Acute Strokes in Patients with COVID-19? American Journal of Neuroradiology. 2020;41(8):E65-E6.

185. Fara MG, Stein LK, Skliut M, Morgello S, Fifi JT, Dhamoon MS. Macrothrombosis and stroke in patients with mild Covid-19 infection. Journal of Thrombosis and Haemostasis. 2020;18(8):2031-3.

186. Cyr DG, Vicidomini CM, Siu NY, Elmann SE. Severe Bilateral Vision Loss in 2 Patients With Coronavirus Disease 2019. J Neuroophthalmol. 2020;40(3):403-5.

187. Al Saiegh F, Ghosh R, Leibold A, et al. Status of SARS-CoV-2 in cerebrospinal fluid of patients with COVID-19 and stroke. Journal of Neurology, Neurosurgery &amp; Psychiatry. 2020;91(8):846-8.

188. Jaunmuktane Z, Mahadeva U, Green A, et al. Microvascular injury and hypoxic damage: emerging neuropathological signatures in COVID-19. Acta Neuropathol. 2020;140(3):397-400.

189. Agarwal A, Vishnu VY, Vibha D, et al. Intracerebral Hemorrhage and SARS-CoV-2: Association or Causation. Ann Indian Acad Neurol. 2020;23(3):261-4.

190. Liu JL, Khawaja AM, Majjhoo AQ. Stroke as a delayed manifestation of multi-organ thromboembolic disease in COVID-19 infection. Journal of the Neurological Sciences. 2020;417.

191. Deliwala S, Abdulhamid S, Abusalih MF, Al-Qasmi MM, Bachuwa G. Encephalopathy as the Sentinel Sign of a Cortical Stroke in a Patient Infected With Coronavirus Disease-19 (COVID-19). Cureus. 2020;12(5):e8121-e.

192. Zhai P, Ding Y, Li Y. The impact of COVID-19 on ischemic stroke. Diagn Pathol. 2020;15(1):78-.

193. Mantero V, Basilico P, Costantino G, Pozzetti U, Rigamonti A, Salmaggi A. Recurrent Transient Ischemic Attack in a Young Patient with COVID-19. J Clin Neurol. 2020;16(3):513-4.

194. Moshayedi P, Ryan TE, Mejia LLP, Nour M, Liebeskind DS. Triage of Acute Ischemic Stroke in Confirmed COVID-19: Large Vessel Occlusion Associated With Coronavirus Infection. Front Neurol. 2020;11:353-.

195. Cavallieri F, Marti A, Fasano A, et al. Prothrombotic state induced by COVID-19 infection as trigger for stroke in young patients: A dangerous association. eNeurologicalSci. 2020;20:100247-.

196. Mahboob S, Boppana SH, Rose NB, Beutler BD, Tabaac BJ. Large vessel stroke and COVID-19: Case report and literature review. eNeurologicalSci. 2020;20:100250-.

197. Zulfiqar AA, Lorenzo-Villalba N, Hassler P, Andrès E. Immune Thrombocytopenic Purpura in a Patient with Covid-19. N Engl J Med. 2020.

198. Avula A, Nalleballe K, Narula N, et al. COVID-19 presenting as stroke. Brain, Behavior, and Immunity. 2020;87:115-9.

199. Doo FX, Kassim G, Lefton DR, Patterson S, Pham H, Belani P. Rare presentations of COVID-19: PRES-like leukoencephalopathy and carotid thrombosis. Clin Imaging. 2020;69:94-101.

200. Li J, Long X, Zhu C, et al. A case of COVID-19 pneumonia with cerebral hemorrhage. Thromb Res. 2020;193:22-4.

201. Guillan M, Villacieros-Alvarez J, Bellido S, et al. Unusual simultaneous cerebral infarcts in multiple arterial territories in a COVID-19 patient. Thromb Res. 2020;193:107-9.

202. Ghosh R, Dubey S, Kanti Ray B, Chatterjee S, Benito-Leon J. COVID-19 Presenting With Thalamic Hemorrhage Unmasking Moyamoya Angiopathy. Can J Neurol Sci. 2020;47(6):849-51.

203. Haddadi K, Ghasemian R, Shafizad M. Basal Ganglia Involvement and Altered Mental Status: A Unique Neurological Manifestation of Coronavirus Disease 2019. Cureus. 2020;12(4):e7869.

204. Craen A, Logan G, Ganti L. Novel Coronavirus Disease 2019 and Subarachnoid Hemorrhage: A Case Report. Cureus. 2020;12(4):e7846.

205. Kim C, Kwak Y, Hwang J, Eun MY. Spontaneous Intracerebral Hemorrhage in a Patient with Asymptomatic 2019 Novel Coronavirus Disease. J Clin Neurol. 2020;16(3):515-7.

206. Hanafi R, Roger PA, Perin B, et al. COVID-19 Neurologic Complication with CNS Vasculitis-Like Pattern. AJNR Am J Neuroradiol. 2020;41(8):1384-7.

207. Matos AR, Quintas-Neves M, Oliveira AI, et al. COVID-19 Associated Central Nervous System Vasculopathy. Can J Neurol Sci. 2021;48(1):139-40.

208. Ostovan VR, Foroughi R, Rostami M, et al. Cerebral venous sinus thrombosis associated with COVID-19: a case series and literature review. J Neurol. 2021.

209. Cavalcanti DD, Raz E, Shapiro M, et al. Cerebral Venous Thrombosis Associated with COVID-19. American Journal of Neuroradiology. 2020;41(8):1370-6.

210. Poillon G, Obadia M, Perrin M, Savatovsky J, Lecler A. Cerebral venous thrombosis associated with COVID-19 infection: Causality or coincidence? Journal of Neuroradiology. 2020.

211. Hemasian H, Ansari B. First case of Covid-19 presented with cerebral venous thrombosis: A rare and dreaded case. Rev Neurol (Paris). 2020;176(6):521-3.

212. Malentacchi M, Gned D, Angelino V, et al. Concomitant brain arterial and venous thrombosis in a COVID-19 patient. Eur J Neurol. 2020;27(9):e38-e9.

213. Rigamonti A, Mantero V, Piamarta F, Spena G, Salmaggi A. Cerebral venous thrombosis associated with coronavirus infection: an underestimated entity? Neurological Sciences. 2020.

214. Klein DE, Libman R, Kirsch C, Arora R. Cerebral venous thrombosis: A typical presentation of COVID-19 in the young. J Stroke Cerebrovasc Dis. 2020;29(8):104989-.

215. Aghayari Sheikh Neshin S, Basirjafari S, Saberi A, Shahhosseini B, Zarei M. Liver abnormality may develop cerebral vein thrombosis in COVID-19. J Neurol Sci. 2020;417:117076.

216. Garaci F, Di Giuliano F, Picchi E, Da Ros V, Floris R. Venous cerebral thrombosis in COVID-19 patient. J Neurol Sci. 2020;414:116871.

217. Chougar L, Mathon B, Weiss N, Degos V, Shor N. Atypical Deep Cerebral Vein Thrombosis with Hemorrhagic Venous Infarction in a Patient Positive for COVID-19. AJNR Am J Neuroradiol. 2020;41(8):1377-9.

218. Fitsiori A, Pugin D, Thieffry C, Lalive P, Vargas MI. Unusual Microbleeds in Brain MRI of Covid-19 Patients. Journal of Neuroimaging. 2020;n/a(n/a).

219. Abdo WF, Broerse CI, Grady BP, et al. Prolonged Unconsciousness Following Severe COVID-19. Neurology. 2021;96(10):e1437-e42.

220. Eden A, Kanberg N, Gostner J, et al. CSF Biomarkers in Patients With COVID-19 and Neurologic Symptoms: A Case Series. Neurology. 2021;96(2):e294-e300.

221. Pugin D, Vargas M-I, Thieffry C, et al. COVID-19-related encephalopathy responsive to high doses glucocorticoids. Neurology. 2020:10.1212/WNL.0000000000010354.

222. Nicholson P, Alshafai L, Krings T. Neuroimaging Findings in Patients with COVID-19. American Journal of Neuroradiology. 2020;41(8):1380-3.

223. Anzalone N, Castellano A, Scotti R, et al. Multifocal laminar cortical brain lesions: a consistent MRI finding in neuro-COVID-19 patients. Journal of Neurology. 2020.

224. Huang H, Eichelberger H, Chan M, et al. Pearls and Oy-sters: Leukoencephalopathy in critically ill COVID-19 patients. Neurology. 2020:10.1212/WNL.0000000000010636.

225. Parauda SC, Gao V, Gewirtz AN, et al. Posterior reversible encephalopathy syndrome in patients with COVID-19. Journal of the neurological sciences. 2020;416:117019-.

226. Benameur K, Agarwal A, Auld SC, et al. Encephalopathy and Encephalitis Associated with Cerebrospinal Fluid Cytokine Alterations and Coronavirus Disease, Atlanta, Georgia, USA, 2020. Emerg Infect Dis. 2020;26(9):2016-21.

227. Andriuta D, Roger P-A, Thibault W, et al. COVID-19 encephalopathy: detection of antibodies against SARS-CoV-2 in CSF. Journal of Neurology. 2020.

228. Edjlali M, Le Gal A, Louvet M, et al. Teaching NeuroImages: Cytotoxic lesions of the corpus callosum in encephalopathic patients with COVID-19. Neurology. 2020;95(22):1021-2.

229. Kishfy L, Casasola M, Banankhah P, et al. Posterior reversible encephalopathy syndrome (PRES) as a neurological association in severe Covid-19. Journal of the neurological sciences. 2020;414:116943-.

230. Franceschi AM, Ahmed O, Giliberto L, Castillo M. Hemorrhagic Posterior Reversible Encephalopathy Syndrome as a Manifestation of COVID-19 Infection. American Journal of Neuroradiology. 2020;41(7):1173-6.

231. Guedj E, Million M, Dudouet P, et al. (18)F-FDG brain PET hypometabolism in post-SARS-CoV-2 infection: substrate for persistent/delayed disorders? Eur J Nucl Med Mol Imaging. 2021;48(2):592-5.

232. Hosseini AA, Shetty AK, Sprigg N, Auer DP, Constantinescu CS. Delirium as a presenting feature in COVID-19: Neuroinvasive infection or autoimmune encephalopathy? Brain, Behavior, and Immunity. 2020;88:68-70.

233. Steininger PA, Seifert F, Balk S, et al. Pearls & Oy-sters: SARS-CoV-2 Infection of the CNS in a Patient With Meningeosis Carcinomatosa. Neurology. 2021;96(10):496-9.

234. Zhou L, Zhang M, Wang J, Gao J. Sars-Cov-2: Underestimated damage to nervous system. Travel Med Infect Dis. 2020:101642.

235. Al Mazrouei SS, Saeed GA, Al Helali AA, Ahmed M. COVID-19-associated encephalopathy: Neurological manifestation of COVID-19. Radiol Case Rep. 2020;15(9):1646-9.

236. Filatov A, Sharma P, Hindi F, Espinosa PS. Neurological complications of coronavirus disease (COVID-19): Encephalopathy. Cureus. 2020;12(3):e7352.

237. Espinosa PS, Rizvi Z, Sharma P, Hindi F, Filatov A. Neurological Complications of Coronavirus Disease (COVID-19): Encephalopathy, MRI Brain and Cerebrospinal Fluid Findings: Case 2. Cureus. 2020;12(5):e7930-e.

238. Pilotto A, Odolini S, Masciocchi S, et al. Steroid-Responsive Encephalitis in Coronavirus Disease 2019. Annals of Neurology. 2020;88(2):423-7.

239. Krett JD, Jewett GAE, Elton-Lacasse C, et al. Hemorrhagic encephalopathy associated with COVID-19. J Neuroimmunol. 2020;346:577326.

240. Shi B-C, Li J, Jiang J-W, Li M-X, Zhang J, Shang X-L. Mild encephalitis/encephalopathy with a reversible splenial lesion secondary to encephalitis complicated by hyponatremia: A case report and literature review. Medicine (Baltimore). 2019;98(47):e17982-e.

241. Kaya Y, Kara S, Akinci C, Kocaman AS. Transient cortical blindness in COVID-19 pneumonia; a PRES-like syndrome: Case report. J Neurol Sci. 2020;413:116858.

242. Djellaoui A, Seddik L, Cleret De Langavant L, Cattan S, Bachoud-Lévi AC, Hosseini H. Posterior reversible encephalopathy syndrome associated with SARS-CoV-2 infection. Journal of Neurology, Neurosurgery &amp; Psychiatry. 2020:jnnp-2020-323923.

243. Poyiadji N, Shahin G, Noujaim D, Stone M, Patel S, Griffith B. COVID-19-associated Acute Hemorrhagic Necrotizing Encephalopathy: CT and MRI Features. Radiology. 2020:201187.

244. Grimaldi S, Lagarde S, Harle JR, Boucraut J, Guedj E. Autoimmune encephalitis concomitant with SARS-CoV-2 infection: insight from (18)F-FDG PET imaging and neuronal autoantibodies. J Nucl Med. 2020.

245. Khoo A, McLoughlin B, Cheema S, et al. Postinfectious brainstem encephalitis associated with SARS-CoV-2. Journal of Neurology, Neurosurgery &amp; Psychiatry. 2020;91(9):1013-4.

246. Dixon L, Varley J, Gontsarova A, et al. COVID-19-related acute necrotizing encephalopathy with brain stem involvement in a patient with aplastic anemia. Neurol Neuroimmunol Neuroinflamm. 2020;7(5):e789.

247. Duong L, Xu P, Liu A. Meningoencephalitis without respiratory failure in a young female patient with COVID-19 infection in Downtown Los Angeles, early April 2020. Brain, behavior, and immunity. 2020;87:33-.

248. Abenza-Abildúa MJ, Novo-Aparicio S, Moreno-Zabaleta R, et al. Encephalopathy in severe SARS-CoV2 infection: Inflammatory or infectious? International journal of infectious diseases : IJID : official publication of the International Society for Infectious Diseases. 2020;98:398-400.

249. Radmanesh A, Derman A, Ishida K. COVID-19-associated delayed posthypoxic necrotizing leukoencephalopathy. Journal of the neurological sciences. 2020;415:116945-.

250. Babar A, Lewandowski U, Capin I, et al. SARS-CoV-2 Encephalitis in a 20-year Old Healthy Female. Pediatr Infect Dis J. 2020.

251. Delamarre L, Gollion C, Grouteau G, et al. COVID-19–associated acute necrotising encephalopathy successfully treated with steroids and polyvalent immunoglobulin with unusual IgG targeting the cerebral fibre network. Journal of Neurology, Neurosurgery &amp; Psychiatry. 2020;91(9):1004-6.

252. Al-Olama M, Rashid A, Garozzo D. COVID-19-associated meningoencephalitis complicated with intracranial hemorrhage: a case report. Acta Neurochir (Wien). 2020;162(7):1495-9.

253. Zambreanu L, Lightbody S, Bhandari M, et al. A case of limbic encephalitis associated with asymptomatic COVID-19 infection. Journal of Neurology, Neurosurgery &amp; Psychiatry. 2020:jnnp-2020-323839.

254. Virhammar J, Kumlien E, Fällmar D, et al. Acute necrotizing encephalopathy with SARS-CoV-2 RNA confirmed in cerebrospinal fluid. Neurology. 2020:10.1212/WNL.0000000000010250.

255. Princiotta Cariddi L, Tabaee Damavandi P, Carimati F, et al. Reversible Encephalopathy Syndrome (PRES) in a COVID-19 patient. Journal of neurology. 2020:1-4.

256. Efe IE, Aydin OU, Alabulut A, Celik O, Aydin K. COVID-19-Associated Encephalitis Mimicking Glial Tumor. World Neurosurg. 2020;140:46-8.

257. Ye M, Ren Y, Lv T. Encephalitis as a clinical manifestation of COVID-19. Brain Behav Immun. 2020.

258. Hayashi M, Sahashi Y, Baba Y, Okura H, Shimohata T. COVID-19-associated mild encephalitis/encephalopathy with a reversible splenial lesion. Journal of the neurological sciences. 2020;415:116941-.

259. Gouse BM, Spears WE, Nieves Archibald A, Montalvo C. Catatonia in a hospitalized patient with COVID-19 and proposed immune-mediated mechanism. Brain, behavior, and immunity. 2020:S0889-1591(20)31528-2.

260. Méndez-Guerrero A, Laespada-García MI, Gómez-Grande A, et al. Acute hypokinetic-rigid syndrome following SARS-CoV-2 infection. Neurology. 2020:10.1212/WNL.0000000000010282.

261. Farhadian S, Glick LR, Vogels CBF, et al. Acute encephalopathy with elevated CSF inflammatory markers as the initial presentation of COVID-19. BMC Neurol. 2020;20(1):248.

262. Cebrian J, Gonzalez-Martinez A, Garcia-Blanco MJ, et al. Headache and impaired consciousness level associated with SARS-CoV-2 in CSF: A case report. Neurology. 2020;95(6):266-8.

263. Lim ST, Janaway B, Costello H, Trip A, Price G. Persistent psychotic symptoms following COVID-19 infection. BJPsych Open. 2020;6(5):e105.

264. De Stefano P, Nencha U, De Stefano L, Megevand P, Seeck M. Focal EEG changes indicating critical illness associated cerebral microbleeds in a Covid-19 patient. Clin Neurophysiol Pract. 2020;5:125-9.

265. Cannac O, Martinez-Almoyna L, Hraiech S. Critical illness-associated cerebral microbleeds in COVID-19 acute respiratory distress syndrome. Neurology. 2020;95(11):498-9.

266. Afshar H, Yassin Z, Kalantari S, et al. Evolution and resolution of brain involvement associated with SARS- CoV2 infection: A close Clinical - Paraclinical follow up study of a case. Mult Scler Relat Disord. 2020;43:102216.

267. Nepal P, Batchala PP, Songmen S, Parashar K, Sapire J. An unresponsive COVID-19 patient. Emerg Radiol. 2020;27(6):755-9.

268. Kulick-Soper CV, McKee JL, Wolf RL, et al. Pearls & Oy-sters: Bilateral globus pallidus lesions in a patient with COVID-19. Neurology. 2020;95(10):454-7.

269. Rasmussen C, Niculescu I, Patel S, Krishnan A. COVID-19 and Involvement of the Corpus Callosum: Potential Effect of the Cytokine Storm? AJNR Am J Neuroradiol. 2020;41(9):1625-8.

270. van den Enden AJM, van Gils L, Labout JAM, van der Jagt M, Moudrous W. Fulminant cerebral edema as a lethal manifestation of COVID-19. Radiol Case Rep. 2020;15(9):1705-8.

271. Fischer D, Threlkeld ZD, Bodien YG, et al. Intact Brain Network Function in an Unresponsive Patient with COVID-19. Ann Neurol. 2020;88(4):851-4.

272. Yong MH, Chan YFZ, Liu J, et al. A Rare Case of Acute Hemorrhagic Leukoencephalitis in a COVID-19 Patient. J Neurol Sci. 2020;416:117035.

273. Mawhinney JA, Wilcock C, Haboubi H, Roshanzamir S. Neurotropism of SARS-CoV-2: COVID-19 presenting with an acute manic episode. BMJ Case Rep. 2020;13(6).

274. Casez O, Willaume G, Grand S, et al. Teaching NeuroImages: SARS-CoV-2-Related Encephalitis: MRI Pattern of Olfactory Tract Involvement. Neurology. 2021;96(4):e645-e6.

275. Ayatollahi P, Tarazi A, Wennberg R. Possible Autoimmune Encephalitis with Claustrum Sign in case of Acute SARS-CoV-2 Infection. Can J Neurol Sci. 2021;48(3):430-2.

276. Reichard RR, Kashani KB, Boire NA, Constantopoulos E, Guo Y, Lucchinetti CF. Neuropathology of COVID-19: a spectrum of vascular and acute disseminated encephalomyelitis (ADEM)-like pathology. Acta Neuropathol. 2020;140(1):1-6.

277. Novi G, Rossi T, Pedemonte E, et al. Acute disseminated encephalomyelitis after SARS-CoV-2 infection. Neurol Neuroimmunol Neuroinflamm. 2020;7(5):e797.

278. Abdi S, Ghorbani A, Fatehi F. The association of SARS-CoV-2 infection and acute disseminated encephalomyelitis without prominent clinical pulmonary symptoms. Journal of the neurological sciences. 2020;416:117001-.

279. Brun G, Hak JF, Coze S, et al. COVID-19-White matter and globus pallidum lesions: Demyelination or small-vessel vasculitis? Neurol Neuroimmunol Neuroinflamm. 2020;7(4).

280. Parsons T, Banks S, Bae C, Gelber J, Alahmadi H, Tichauer M. COVID-19-associated acute disseminated encephalomyelitis (ADEM). Journal of neurology. 2020:1-4.

281. Zoghi A, Ramezani M, Roozbeh M, Darazam IA, Sahraian MA. A case of possible atypical demyelinating event of the central nervous system following COVID-19. Mult Scler Relat Disord. 2020;44:102324.

282. Zanin L, Saraceno G, Panciani PP, et al. SARS-CoV-2 can induce brain and spine demyelinating lesions. Acta Neurochir (Wien). 2020;162(7):1491-4.

283. AlKetbi R, AlNuaimi D, AlMulla M, et al. Acute myelitis as a neurological complication of Covid-19: A case report and MRI findings. Radiol Case Rep. 2020;15(9):1591-5.

284. Munz M, Wessendorf S, Koretsis G, et al. Acute transverse myelitis after COVID-19 pneumonia. Journal of neurology. 2020;267(8):2196-7.

285. Zhou S, Jones-Lopez EC, Soneji DJ, Azevedo CJ, Patel VR. Myelin Oligodendrocyte Glycoprotein Antibody-Associated Optic Neuritis and Myelitis in COVID-19. J Neuroophthalmol. 2020;40(3):398-402.

286. Maideniuc C, Memon AB. Acute necrotizing myelitis and acute motor axonal neuropathy in a COVID-19 patient. J Neurol. 2020:1-3.

287. Utukuri PS, Bautista A, Lignelli A, Moonis G. Possible Acute Disseminated Encephalomyelitis Related to Severe Acute Respiratory Syndrome Coronavirus 2 Infection. American Journal of Neuroradiology. 2020.

288. Zachariadis A, Tulbu A, Strambo D, Dumoulin A, Di Virgilio G. Transverse myelitis related to COVID-19 infection. Journal of Neurology. 2020.

289. Chow CCN, Magnussen J, Ip J, Su Y. Acute transverse myelitis in COVID-19 infection. BMJ Case Rep. 2020;13(8):e236720.

290. Giorgianni A, Vinacci G, Agosti E, et al. Transient acute-onset tetraparesis in a COVID-19 patient. Spinal Cord. 2020.

291. Sotoca J, Rodríguez-Álvarez Y. COVID-19-associated acute necrotizing myelitis. Neurol Neuroimmunol Neuroinflamm. 2020;7(5):e803.

292. Domingues RB, Mendes-Correa MC, de Moura Leite FBV, et al. First case of SARS-COV-2 sequencing in cerebrospinal fluid of a patient with suspected demyelinating disease. Journal of neurology. 2020:1-3.

293. Anand P, Al-Faraj A, Sader E, et al. Seizure as the presenting symptom of COVID-19: A retrospective case series. Epilepsy Behav. 2020;112:107335-.

294. Hepburn M, Mullaguri N, George P, et al. Acute Symptomatic Seizures in Critically Ill Patients with COVID-19: Is There an Association? Neurocrit Care. 2020:1-5.

295. Somani S, Pati S, Gaston T, Chitlangia A, Agnihotri S. De Novo Status Epilepticus in patients with COVID-19. Ann Clin Transl Neurol. 2020;7(7):1240-4.

296. Lyons S, O'Kelly B, Woods S, et al. Seizure with CSF lymphocytosis as a presenting feature of COVID-19 in an otherwise healthy young man. Seizure. 2020;80:113-4.

297. Fasano A, Cavallieri F, Canali E, Valzania F. First motor seizure as presenting symptom of SARS-CoV-2 infection. Neurological sciences : official journal of the Italian Neurological Society and of the Italian Society of Clinical Neurophysiology. 2020;41(7):1651-3.

298. Kadono Y, Nakamura Y, Ogawa Y, et al. A case of COVID-19 infection presenting with a seizure following severe brain edema. Seizure. 2020;80:53-5.

299. Logmin K, Karam M, Schichel T, Harmel J, Wojtecki L. Non-epileptic seizures in autonomic dysfunction as the initial symptom of COVID-19. Journal of Neurology. 2020;267(9):2490-1.

300. Sohal S, Mansur M. COVID-19 Presenting with Seizures. IDCases. 2020;20:e00782-e.

301. Balloy G, Leclair-Visonneau L, Pereon Y, et al. Non-lesional status epilepticus in a patient with coronavirus disease 2019. Clin Neurophysiol. 2020;131(8):2059-61.

302. Gomez-Enjuto S, Hernando-Requejo V, Lapena-Motilva J, et al. Verapamil as treatment for refractory status epilepticus secondary to PRES syndrome on a SARS-Cov-2 infected patient. Seizure. 2020;80:157-8.

303. Le Guennec L, Devianne J, Jalin L, et al. Orbitofrontal involvement in a neuroCOVID-19 patient. Epilepsia. 2020;61(8):e90-e4.

304. Cunha P, Herlin B, Vassilev K, et al. Movement disorders as a new neurological clinical picture in severe SARS-CoV-2 infection. Eur J Neurol.n/a(n/a).

305. Rábano-Suárez P, Bermejo-Guerrero L, Méndez-Guerrero A, et al. Generalized myoclonus in COVID-19. Neurology. 2020;95(6):e767-e72.

306. Fadakar N, Ghaemmaghami S, Masoompour SM, et al. A First Case of Acute Cerebellitis Associated with Coronavirus Disease (COVID-19): a Case Report and Literature Review. Cerebellum. 2020;19(6):911-4.

307. Shah PB, Desai SD. Opsoclonus myoclonus ataxia syndrome (OMAS) in the setting of COVID-19 infection. Neurology. 2020.

308. Fragiel M, Miro O, Llorens P, et al. Incidence, clinical, risk factors and outcomes of Guillain-Barre in Covid-19. Ann Neurol. 2021;89(3):598-603.

309. Keddie S, Pakpoor J, Mousele C, et al. Epidemiological and cohort study finds no association between COVID-19 and Guillain-Barre syndrome. Brain. 2021;144(2):682-93.

310. Filosto M, Cotti Piccinelli S, Gazzina S, et al. Guillain-Barre syndrome and COVID-19: an observational multicentre study from two Italian hotspot regions. J Neurol Neurosurg Psychiatry. 2020.

311. Tatu L, Nono S, Grácio S, Koçer S. Guillain-Barré syndrome in the COVID-19 era: another occasional cluster? Journal of neurology. 2020:1-3.

312. Manganotti P, Bellavita G, D'Acunto L, et al. Clinical neurophysiology and cerebrospinal liquor analysis to detect Guillain-Barré syndrome and polyneuritis cranialis in COVID-19 patients: A case series. Journal of Medical Virology.n/a(n/a).

313. Lascano AM, Epiney J-B, Coen M, et al. SARS-CoV-2 and Guillain–Barré syndrome: AIDP variant with a favourable outcome. Eur J Neurol. 2020;27(9):1751-3.

314. Ebrahimzadeh SA, Ghoreishi A, Rahimian N. Guillain-Barré Syndrome associated with the Coronavirus Disease 2019 (COVID-19). Neurol Clin Pract. 2020.

315. Assini A, Benedetti L, Di Maio S, Schirinzi E, Del Sette M. New clinical manifestation of COVID-19 related Guillain-Barrè syndrome highly responsive to intravenous immunoglobulins: two Italian cases. Neurological Sciences. 2020;41(7):1657-8.

316. Dinkin M, Gao V, Kahan J, et al. COVID-19 presenting with ophthalmoparesis from cranial nerve palsy. Neurology. 2020.

317. Elkhouly A, Kaplan AC. Noteworthy Neurological Manifestations Associated With COVID-19 Infection. Cureus. 2020;12(7):e8992-e.

318. Paybast S, Gorji R, Mavandadi S. Guillain-Barré Syndrome as a Neurological Complication of Novel COVID-19 Infection: A Case Report and Review of the Literature. Neurologist. 2020;25(4):101-3.

319. Oguz-Akarsu E, Ozpar R, Mirzayev H, et al. Guillain-Barré Syndrome in a Patient With Minimal Symptoms of COVID-19 Infection. Muscle & Nerve. 2020;62(3):E54-E7.

320. Helbok R, Beer R, Löscher W, et al. Guillain-Barré syndrome in a patient with antibodies against SARS-COV-2. Eur J Neurol. 2020;27(9):1754-6.

321. Farzi MA, Ayromlou H, Jahanbakhsh N, Bavil PH, Janzadeh A, Shayan FK. Guillain-Barré syndrome in a patient infected with SARS-CoV-2, a case report. J Neuroimmunol. 2020;346:577294-.

322. Alberti P, Beretta S, Piatti M, et al. Guillain-Barré syndrome related to COVID-19 infection. Neurol Neuroimmunol Neuroinflamm. 2020;7(4):e741.

323. Padroni M, Mastrangelo V, Asioli GM, et al. Guillain-Barré syndrome following COVID-19: new infection, old complication? Journal of neurology. 2020;267(7):1877-9.

324. Agosti E, Giorgianni A, D'Amore F, Vinacci G, Balbi S, Locatelli D. Is Guillain-Barrè syndrome triggered by SARS-CoV-2? Case report and literature review. Neurological sciences : official journal of the Italian Neurological Society and of the Italian Society of Clinical Neurophysiology. 2020:1-6.

325. Ottaviani D, Boso F, Tranquillini E, et al. Early Guillain-Barré syndrome in coronavirus disease 2019 (COVID-19): a case report from an Italian COVID-hospital. Neurological sciences : official journal of the Italian Neurological Society and of the Italian Society of Clinical Neurophysiology. 2020;41(6):1351-4.

326. Riva N, Russo T, Falzone YM, et al. Post-infectious Guillain–Barré syndrome related to SARS-CoV-2 infection: a case report. Journal of Neurology. 2020;267(9):2492-4.

327. Zhao H, Shen D, Zhou H, Liu J, Chen S. Guillain-Barre syndrome associated with SARS-CoV-2 infection: causality or coincidence? Lancet Neurol. 2020.

328. Webb S, Wallace VC, Martin-Lopez D, Yogarajah M. Guillain-Barré syndrome following COVID-19: a newly emerging post-infectious complication. BMJ Case Rep. 2020;13(6):e236182.

329. Juliao Caamaño DS, Alonso Beato R. Facial diplegia, a possible atypical variant of Guillain-Barré Syndrome as a rare neurological complication of SARS-CoV-2. Journal of clinical neuroscience : official journal of the Neurosurgical Society of Australasia. 2020;77:230-2.

330. Alberti P, Beretta S, Piatti M, et al. Guillain-Barré syndrome related to COVID-19 infection. Neurology - Neuroimmunology Neuroinflammation. 2020;7(4):e741.

331. El Otmani H, El Moutawakil B, Rafai MA, et al. Covid-19 and Guillain-Barré syndrome: More than a coincidence! Rev Neurol (Paris). 2020;176(6):518-9.

332. Abrams RMC, Kim BD, Markantone DM, et al. Severe rapidly progressive Guillain-Barré syndrome in the setting of acute COVID-19 disease. J Neurovirol. 2020:1-3.

333. Su XW, Palka SV, Rao RR, Chen FS, Brackney CR, Cambi F. SARS-CoV-2-associated Guillain-Barré syndrome with dysautonomia. Muscle & nerve. 2020;62(2):E48-E9.

334. Rana S, Lima AA, Chandra R, et al. Novel Coronavirus (COVID-19)-Associated Guillain-Barré Syndrome: Case Report. J Clin Neuromuscul Dis. 2020;21(4):240-2.

335. Camdessanche JP, Morel J, Pozzetto B, Paul S, Tholance Y, Botelho-Nevers E. COVID-19 may induce Guillain-Barré syndrome. Rev Neurol (Paris). 2020;176(6):516-8.

336. Sedaghat Z, Karimi N. Guillain Barre syndrome associated with COVID-19 infection: A case report. J Clin Neurosci. 2020.

337. Scheidl E, Canseco DD, Hadji-Naumov A, Bereznai B. Guillain-Barré syndrome during SARS-CoV-2 pandemic: A case report and review of recent literature. J Peripher Nerv Syst. 2020;25(2):204-7.

338. Pelea T, Reuter U, Schmidt C, Laubinger R, Siegmund R, Walther BW. SARS-CoV-2 associated Guillain–Barré syndrome. Journal of Neurology. 2020.

339. Kilinc D, van de Pasch S, Doets AY, Jacobs BC, van Vliet J, Garssen MPJ. Guillain-Barré syndrome after SARS-CoV-2 infection. Eur J Neurol. 2020:10.1111/ene.14398.

340. Tiet MY, AlShaikh N. Guillain-Barré syndrome associated with COVID-19 infection: a case from the UK. BMJ Case Rep. 2020;13(7):e236536.

341. Naddaf E, Laughlin RS, Klein CJ, et al. Guillain-Barr&#xe9; Syndrome in a Patient With Evidence of Recent SARS-CoV-2 Infection. Mayo Clinic Proceedings. 2020;95(8):1799-801.

342. Hutchins KL, Jansen JH, Comer AD, et al. COVID-19–Associated Bifacial Weakness with Paresthesia Subtype of Guillain-Barré Syndrome. American Journal of Neuroradiology. 2020.

343. Sancho-Saldaña A, Lambea-Gil Á, Liesa JLC, et al. Guillain-Barré syndrome associated with leptomeningeal enhancement following SARS-CoV-2 infection. Clin Med (Lond). 2020;20(4):e93-e4.

344. Bracaglia M, Naldi I, Govoni A, Brillanti Ventura D, De Massis P. Acute inflammatory demyelinating polyneuritis in association with an asymptomatic infection by SARS-CoV-2. J Neurol. 2020;267(11):3166-8.

345. Pfefferkorn T, Dabitz R, von Wernitz-Keibel T, Aufenanger J, Nowak-Machen M, Janssen H. Acute polyradiculoneuritis with locked-in syndrome in a patient with Covid-19. J Neurol. 2020;267(7):1883-4.

346. Wada S, Nagasaki Y, Arimizu Y, et al. Neurological Disorders Identified during Treatment of a SARS-CoV-2 Infection. Intern Med. 2020;59(17):2187-9.

347. Chan JL, Ebadi H, Sarna JR. Guillain-Barre Syndrome with Facial Diplegia Related to SARS-CoV-2 Infection. Can J Neurol Sci. 2020;47(6):852-4.

348. Toscano G, Palmerini F, Ravaglia S, et al. Guillain–Barré Syndrome Associated with SARS-CoV-2. N Engl J Med. 2020.

349. Gutierrez-Ortiz C, Mendez A, Rodrigo-Rey S, et al. Miller Fisher Syndrome and polyneuritis cranialis in COVID-19. Neurology. 2020.

350. Reyes-Bueno JA, García-Trujillo L, Urbaneja P, et al. Miller-Fisher syndrome after SARS-CoV-2 infection. Eur J Neurol. 2020;27(9):1759-61.

351. Manganotti P, Pesavento V, Buoite Stella A, et al. Miller Fisher syndrome diagnosis and treatment in a patient with SARS-CoV-2. J Neurovirol. 2020;26(4):605-6.

352. Ray A. Miller Fisher syndrome and COVID-19: is there a link? BMJ Case Rep. 2020;13(8):e236419.

353. Todisco M, Alfonsi E, Arceri S, et al. Isolated bulbar palsy after SARS-CoV-2 infection. Lancet Neurol. 2021;20(3):169-70.

354. Decavel P, Petit C, Tatu L. Tapia syndrome at the time of the COVID-19 pandemic: Lower cranial neuropathy following prolonged intubation. Neurology. 2020;95(7):312-3.

355. Diprose WK, Bainbridge L, Frith RW, Anderson NE. Bilateral upper limb neuropathies following prone ventilation for COVID-19 pneumonia. Neurology: Clinical Practice. 2020:10.1212/CPJ.0000000000000944.

356. Abdelnour L, Eltahir Abdalla M, Babiker S. COVID 19 infection presenting as motor peripheral neuropathy. Journal of the Formosan Medical Association = Taiwan yi zhi. 2020;119(6):1119-20.

357. Homma Y, Watanabe M, Inoue K, Moritaka T. Coronavirus Disease-19 Pneumonia with Facial Nerve Palsy and Olfactory Disturbance. Intern Med. 2020;59(14):1773-5.

358. Aoyagi Y, Ohashi M, Funahashi R, Otaka Y, Saitoh E. Oropharyngeal Dysphagia and Aspiration Pneumonia Following Coronavirus Disease 2019: A Case Report. Dysphagia. 2020;35(4):545-8.

359. Siepmann T, Kitzler HH, Lueck C, Platzek I, Reichmann H, Barlinn K. Neuralgic amyotrophy following infection with SARS-CoV-2. Muscle Nerve. 2020;62(4):E68-E70.

360. Goh Y, Beh DLL, Makmur A, Somani J, Chan ACY. Pearls & Oy-sters: Facial nerve palsy in COVID-19 infection. Neurology. 2020;95(8):364-7.

361. Figueiredo R, Falcao V, Pinto MJ, Ramalho C. Peripheral facial paralysis as presenting symptom of COVID-19 in a pregnant woman. BMJ Case Rep. 2020;13(8).

362. Escalada Pellitero S, Garriga Ferrer-Bergua L. Report of a patient with neurological symptoms as the sole manifestation of SARS-CoV-2 infection. Neurología 2020;35(4):271-2.

363. Falcone MM, Rong AJ, Salazar H, Redick DW, Falcone S, Cavuoto KM. Acute abducens nerve palsy in a patient with the novel coronavirus disease (COVID-19). J AAPOS. 2020;24(4):216-7.

364. Restivo DA, Centonze D, Alesina A, Marchese-Ragona R. Myasthenia Gravis Associated With SARS-CoV-2 Infection. Annals of internal medicine. 2020:L20-0845.

365. Madia F, Merico B, Primiano G, Cutuli SL, De Pascale G, Servidei S. Acute myopathic quadriplegia in patients with COVID-19 in the intensive care unit. Neurology. 2020;95(11):492-4.

366. Tankisi H, Tankisi A, Harbo T, Markvardsen LK, Andersen H, Pedersen TH. Critical illness myopathy as a consequence of Covid-19 infection. Clinical Neurophysiology. 2020;131(8):1931-2.

367. Zhang H, Charmchi Z, Seidman RJ, Anziska Y, Velayudhan V, Perk J. COVID-19-associated myositis with severe proximal and bulbar weakness. Muscle Nerve. 2020;62(3):E57-E60.

368. Chaumont H, San-Galli A, Martino F, et al. Mixed central and peripheral nervous system disorders in severe SARS-CoV-2 infection. J Neurol. 2020;267(11):3121-7.

369. Krajewski PK, Szepietowski JC, Maj J. Cutaneous hyperesthesia: A novel manifestation of COVID-19. Brain Behav Immun. 2020;87:188.

370. Yin R, Feng W, Wang T, et al. Concomitant neurological symptoms observed in a patient diagnosed with coronavirus disease 2019. J Med Virol. 2020;92(10):1782-4.

371. Kong Z, Wang J, Li T, Zhang Z, Jian J. 2019 novel coronavirus pneumonia with onset of dizziness: a case report. Ann Transl Med. 2020;8(7):506.

372. Janocha-Litwin J, Jachman-Kapulka J, Czarnecki M, Krause K, Simon K. Neurologic complications of SARS-CoV-2 infection in a 66-year-old man. Pol Arch Intern Med. 2020;130(11):1000-2.

373. LaRovere KL, Riggs BJ, Poussaint TY, et al. Neurologic Involvement in Children and Adolescents Hospitalized in the United States for COVID-19 or Multisystem Inflammatory Syndrome. JAMA Neurol. 2021.

374. Feldstein LR, Tenforde MW, Friedman KG, et al. Characteristics and Outcomes of US Children and Adolescents With Multisystem Inflammatory Syndrome in Children (MIS-C) Compared With Severe Acute COVID-19. JAMA. 2021.

375. Ma N, Li P, Wang X, et al. Ocular Manifestations and Clinical Characteristics of Children With Laboratory-Confirmed COVID-19 in Wuhan, China. JAMA Ophthalmology. 2020.

376. Abdel-Mannan O, Eyre M, Löbel U, et al. Neurologic and Radiographic Findings Associated With COVID-19 Infection in Children. JAMA Neurology. 2020.

377. Schupper AJ, Yaeger KA, Morgenstern PF. Neurological manifestations of pediatric multi-system inflammatory syndrome potentially associated with COVID-19. Childs Nerv Syst. 2020;36(8):1579-80.

378. Turbin RE, Wawrzusin PJ, Sakla NM, et al. Orbital cellulitis, sinusitis and intracranial abnormalities in two adolescents with COVID-19. Orbit. 2020;39(4):305-10.

379. Bastidas HI, Márquez-Pérez T, García-Salido A, et al. Cerebral venous sinus thrombosis in a pediatric patient with COVID-19. Neurol Clin Pract. 2020.

380. Frank CHM, Almeida TVR, Marques EA, et al. Guillain–Barré Syndrome Associated with SARS-CoV-2 Infection in a Pediatric Patient. Journal of Tropical Pediatrics. 2020.

381. Abel D, Shen MY, Abid Z, et al. Encephalopathy and bilateral thalamic lesions in a child with MIS-C associated with COVID-19. Neurology. 2020:10.1212/WNL.0000000000010652.

382. Mirzaee SMM, Gonçalves FG, Mohammadifard M, Tavakoli SM, Vossough A. Focal Cerebral Arteriopathy in a COVID-19 Pediatric Patient. Radiology. 2020:202197.

383. Chacón-Aguilar R, Osorio-Cámara JM, Sanjurjo-Jimenez I, González-González C, López-Carnero J, Pérez-Moneo B. COVID-19: Fever syndrome and neurological symptoms in a neonate. An Pediatr (Engl Ed). 2020;92(6):373-4.

384. Dugue R, Cay-Martinez KC, Thakur K, et al. Neurologic manifestations in an infant with COVID-19. Neurology. 2020.

385. Farley M, Zuberi J. COVID-19 Precipitating Status Epilepticus in a Pediatric Patient. Am J Case Rep. 2020;21:e925776.

386. Enner S, Hormozdyaran S, Varughese R, et al. Central Apnea in an Adolescent With COVID-19. Pediatr Neurol. 2020;110:87-8.

387. McAbee GN, Brosgol Y, Pavlakis S, Agha R, Gaffoor M. Encephalitis Associated with COVID-19 Infection in an 11-Year-Old Child. Pediatr Neurol. 2020;109:94-.

388. Bhatta S, Sayed A, Ranabhat B, Bhatta RK, Acharya Y. New-Onset Seizure as the Only Presentation in a Child With COVID-19. Cureus. 2020;12(6):e8820-e.

389. Khalifa M, Zakaria F, Ragab Y, et al. Guillain-Barré Syndrome Associated With Severe Acute Respiratory Syndrome Coronavirus 2 Detection and Coronavirus Disease 2019 in a Child. Journal of the Pediatric Infectious Diseases Society. 2020.
